# Pancreatic β cell microRNA-26a alleviates type 2 diabetes by improving peripheral insulin sensitivity and preserving β cell function

**DOI:** 10.1371/journal.pbio.3000603

**Published:** 2020-02-24

**Authors:** Haixia Xu, Xiao Du, Jia Xu, Yu Zhang, Yan Tian, Geng Liu, Xiuxuan Wang, Meilin Ma, Wenya Du, Yu Liu, Lunzhi Dai, Wendong Huang, Nanwei Tong, Yuquan Wei, Xianghui Fu

**Affiliations:** 1 Division of Endocrinology and Metabolism, National Clinical Research Center for Geriatrics, State Key Laboratory of Biotherapy and Cancer Center, West China Hospital, Sichuan University and Collaborative Innovation Center of Biotherapy, Chengdu, China; 2 Department of Gastrointestinal Surgery, West China Hospital, Sichuan University, Chengdu, China; 3 Department of General Surgery, Yaan People's Hospital, Yaan, Sichuan, China; 4 State Key Laboratory of Biotherapy and Cancer Center, West China Hospital, Sichuan University and Collaborative Innovation Center of Biotherapy, Chengdu, China; 5 Department of General Practice and Lab of PTM, State Key Laboratory of Biotherapy, West China Hospital, Sichuan University and Collaborative Innovation Center of Biotherapy, Chengdu, China; 6 Department of Diabetes Complications and Metabolism, Beckman Research Institute of City of Hope, Duarte, California, United States of America; 7 Division of Endocrinology and Metabolism, West China Hospital, Sichuan University, Chengdu, China; Peking University, CHINA

## Abstract

Type 2 diabetes (T2D) is characterized by insulin resistance along with pancreatic β cell failure. β cell factors are traditionally thought to control glucose homeostasis by modulating insulin levels, not insulin sensitivity. Exosomes are emerging as new regulators of intercellular communication. However, the role of β-cell–derived exosomes in metabolic homeostasis is poorly understood. Here, we report that microRNA-26a (miR-26a) in β cells not only modulates insulin secretion and β cell replication in an autocrine manner but also regulates peripheral insulin sensitivity in a paracrine manner through circulating exosomes. MiR-26a is reduced in serum exosomes of overweight humans and is inversely correlated with clinical features of T2D. Moreover, miR-26a is down-regulated in serum exosomes and islets of obese mice. Using miR-26a knockin and knockout mouse models, we showed that miR-26a in β cells alleviates obesity-induced insulin resistance and hyperinsulinemia. Mechanistically, miR-26a in β cells enhances peripheral insulin sensitivity via exosomes. Meanwhile, miR-26a prevents hyperinsulinemia through targeting several critical regulators of insulin secretion and β cell proliferation. These findings provide a new paradigm for the far-reaching systemic functions of β cells and offer opportunities for the treatment of T2D.

## Abstract 2

A study using mouse models and human samples reveals a previously unknown role for pancreatic β-cell regulators in glucose homeostasis, in which β cell miR-26a not only modulates insulin secretion and β cell replication in an autocrine manner but also regulates peripheral insulin sensitivity in a paracrine manner through circulating exosomes.

## Introduction

Type 2 diabetes (T2D) is a rapidly rising epidemic worldwide owing to a multifactorial combination of genetic and environmental factors [1,2]. T2D increases the risk of multiple diseases such as cardiovascular complications and certain cancers, leading to a consequent increase in mortality, morbidity, and economic burden [3]. On the therapeutic side, the results from lifestyle-oriented and pharmacologic interventions for T2D are generally disappointing. Therefore, an understanding of the precise mechanisms underlying the pathogenesis of T2D, which will help uncover novel therapeutic targets, aid in the development of new drugs, and ultimately combat the looming T2D epidemic and diabetic complications, is urgently needed.

The etiology of T2D involves insulin resistance along with pancreatic β cell failure. Insulin resistance, also known as low insulin sensitivity, is a common feature of many human diseases [4,5]. Restoring insulin sensitivity has thus become increasingly important in the development of effective approaches to treat these disorders [6]. Notably, pancreatic β cells secrete insulin, which plays a fundamental role in systemic glucose homeostasis and diabetes [7]. A number of β cell factors have been identified to modulate glucose homeostasis by controlling the levels of insulin [8]. However, it remains unknown whether β cell regulators could contribute to metabolic homeostasis independent of insulin levels.

Under prediabetic, insulin-resistant conditions, pancreatic islets respond to increased metabolic overload with increased β cell mass and elevated insulin secretion, which generates compensatory hyperinsulinemia to maintain blood glucose levels within the normal range. Compensatory β cell hyperplasia, the main source of increased β cell mass, is associated with an increase in β cell replication and is governed by a variety of regulators such as insulin receptor substrate 2 (*IRS-2*), forkhead box O transcription factor 1 (*Foxo1*), and AKT serine/threonine kinase (*Akt*) [9,10]. Glucose-stimulated insulin secretion (GSIS) is biphasic, characterized by a rapid, transient first phase and a gradually developing sustained second phase. Specifically, cortical filamentous actin (F-actin) remodeling oversees insulin granule access to the release site of the plasma membrane and is required for the second phase of GSIS [11]. Although hyperinsulinemia is traditionally viewed as compensation for insulin resistance, increasing evidence suggests that sustained obesity-induced hyperinsulinemia can further exacerbate insulin resistance [12]. Therefore, mild suppression of hyperinsulinemia may be a better approach to treat obesity and insulin resistance [13,14]. In this regard, a therapeutic target that simultaneously increases insulin sensitivity and suppresses hyperinsulinemia could be more effective and beneficial for the treatment of T2D.

MicroRNAs (miRNAs) play important roles in virtually all aspects of eukaryotic biological processes and contribute to many human diseases, including T2D [15–17]. MiR-26a is highly expressed in multiple human tissues and plays important roles in various biological and physiological processes [15,18–20]. Specifically, miR-26a has been indicated to increase insulin synthesis in vitro [21]. We recently demonstrated that miR-26a promotes pancreatic cell differentiation [20], enhances insulin sensitivity, and prevents obesity-induced metabolic abnormalities in the liver [22]. However, the role of pancreatic β-cell miR-26a in human diseases, including T2D, has not yet been investigated.

MiRNAs were traditionally thought to act within the cells in which they are generated. However, recent data have shown that many miRNAs are found in secreted exosomes, allowing them to act locally or at distal sites via the circulation [23,24]. Exosomes, which are small extracellular vesicles (EVs) 20–140 nM in size, carry diverse bioactive molecules that can mediate the crosstalk between organs. Exosomal miRNAs are now viewed as an additional mechanism for intercellular communication and are involved in various pathophysiological processes [25–27]. The significance of exosomal miRNAs in metabolic disease, especially T2D, has recently emerged [28]. For example, exosomal miRNAs from adipose and adipose tissue macrophages can modulate gene expression and insulin resistance in distant tissues [29,30]. However, the role of exosomal miRNAs in β cells, one of the most important endocrine cells, in systemic metabolic homeostasis and T2D remains unknown.

Here, we report that miR-26a in β cells prevents obesity-induced hyperinsulinemia and insulin resistance by functioning both locally and distally. MiR-26a is significantly reduced in serum exosomes from obese mice and humans. β-cell–derived exosomal miR-26a markedly enhances insulin sensitivity and metabolic homeostasis in distal tissues. Meanwhile, miR-26a inhibits obesity-induced β cell hyperplasia and GSIS by decreasing β cell replication and impeding actin remodeling, thereby reducing hyperinsulinemia. These results thus reveal a new, to our knowledge, mechanism of β cell factors in interorgan crosstalk and suggest that miR-26a is a potential target with broad applicability for the treatment of insulin resistance and T2D.

## Results

### Serum exosomal miR-26a is reduced in obese humans and mice

Given the significance of insulin resistance and the increasing importance of exosomes in intracellular communication, we explored the potential correlation between exosomal miRNAs and insulin resistance. We purified serum exosomes from a cohort of humans. These vesicles were 40–80 nM in diameter and enriched in exosomal markers tumor susceptibility gene 101 protein (TSG101) and CD63 molecule (CD63), as revealed by transmission electron microscopy, NanoSight, and western blotting (S1A–S1C Fig).

**Fig 1:**
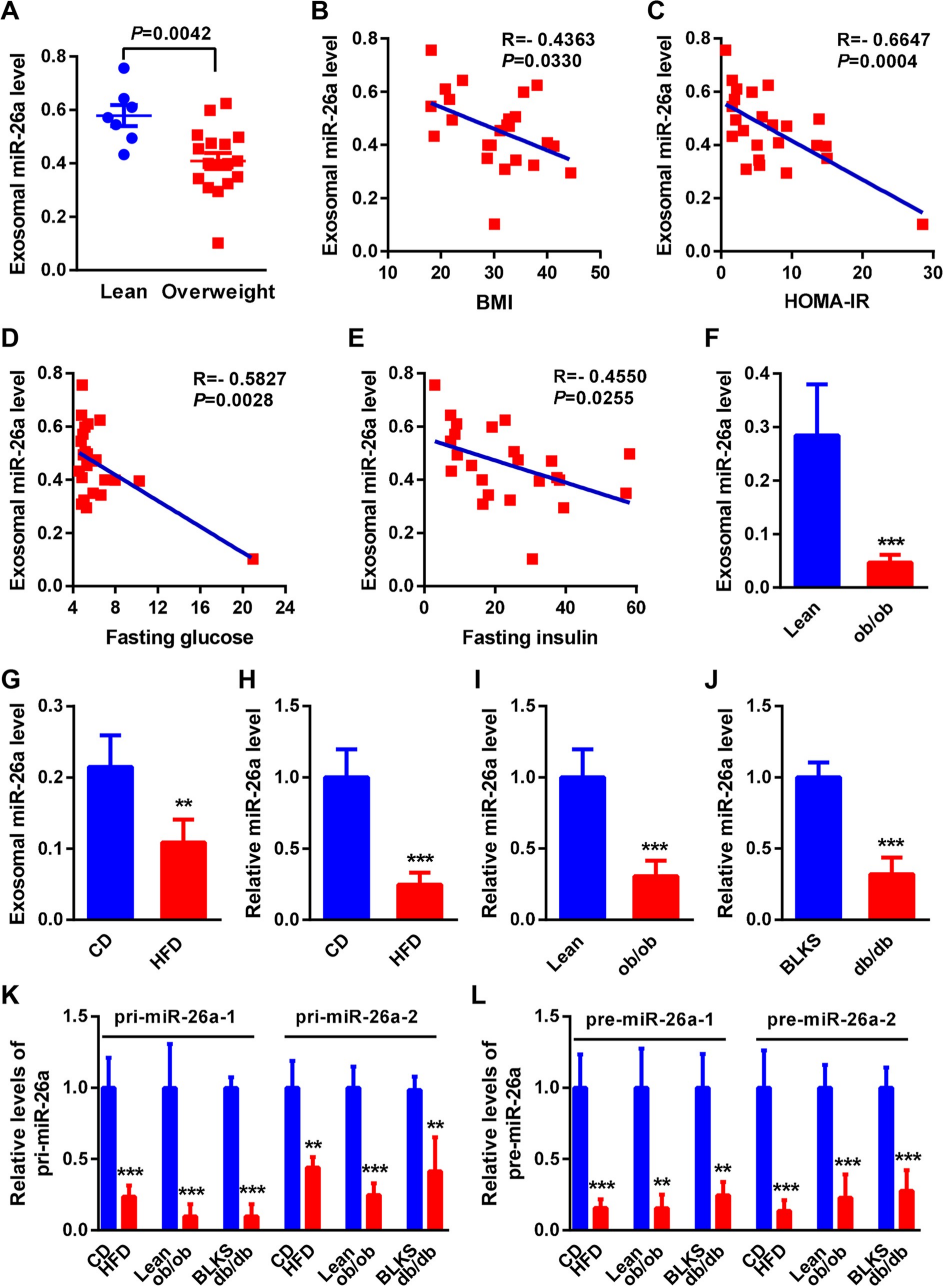
MiR-26a is reduced in serum exosomes and islets during obesity. (A) QRT-PCR analysis of human miR-26a expression in serum exosomes of lean (*n* = 7) and obese (*n* = 17, BMI > 25) individuals. (B–E) Correlation between serum exosomal miR-26a levels and BMI (B), HOMA-IR (C), blood fasting glucose (D), or blood fasting insulin (E) in a human cohort (*n* = 24). (F and G) Levels of mouse miR-26a in serum exosomes of ob/ob mice (*n* = 4) (F) or WT DIO mice (*n* = 4) (G). (H–J) Expression of miR-26a in islets of WT DIO mice (*n* = 4) (H), ob/ob mice (*n* = 4) (I), or db/db mice (*n* = 4) (J). (K and L) Expression of pre-miR-26a (K) or pri-miR-26a (L) in islets of WT DIO mice (*n* = 4), ob/ob mice (*n* = 3–4), or db/db mice (*n* = 3–4). The data underlying this figure may be found in S1 Data. Data are shown as mean ± SD. **P < 0.01, ***P < 0.005, Student *t* test. BLKS, C57BLKS/J, also known as Black Kaliss J; BMI, body mass index; CD, chow diet; db/db mice, leptin-receptor–deficient mice; DIO, diet-induced obese; HFD, high-fat diet; HOMA-IR, homeostatic model assessment index of insulin resistance; ob/ob mice, leptin-deficient mice; pre-miR-26a, precursor miR-26a; pri-miR-26a, primary miR-26a; QRT-PCR, quantitative reverse transcriptase PCR; WT, wild type.

Quantitative reverse transcriptase PCR (QRT-PCR) profiling of serum exosomes for 12 human miRNAs (S1 Table) that have been shown to regulate insulin sensitivity [15] revealed two differentially expressed miRNAs: miR-26a and miR-221-3p. MiR-26a was significantly reduced in overweight individuals (body mass index [BMI] > 25) compared with lean individuals (Fig 1A), consistent with the enhancing effect of miR-26a on insulin sensitivity (S1 Table). Notably, the levels of miR-26a were inversely correlated with BMI, the subjects’ homeostatic model assessment index of insulin resistance (HOMA-IR), fasting blood glucose, and fasting insulin levels (Fig 1B–1E). Furthermore, the levels of miR-26a had significant inverse correlations with HbA1c and triglycerides in overweight individuals (S1D and S1E Fig). In contrast, miR-221-3p was remarkably increased in overweight individuals (S1F Fig), consistent with its suppressive effect on insulin sensitivity. However, the levels of miR-221-3p had no significant correlations with BMI, HOMA-IR, fasting blood glucose, or fasting insulin levels (S1G Fig). Therefore, we chose miR-26a for further analysis.

We observed a similar reduction in exosomal miR-26a levels in genetically obese leptin-deficient (ob/ob) mice (Fig 1F), and diet-induced obese (DIO) mice (Fig 1G), developed by feeding wild-type (WT) mice a high-fat diet (HFD) for 16 weeks. Taken together, these results reveal a decrease in miR-26a in serum exosomes in obese humans and mice that is closely associated with clinical features of obesity and T2D.

### MiR-26a is reduced in islets of obese mice

Insulin resistance occurs in peripheral tissues, including liver, adipose, and muscle tissue. Given that exosomes primarily mediate organ–organ crosstalk, we hypothesized that other obesity-associated organs such as the kidney, heart, and pancreas might account for the reduction in miR-26a in circulating exosomes. MiR-26a expression is unchanged in the kidney and the heart during obesity, as we reported previously [22]. Intriguingly, miR-26a was significantly reduced in isolated islets from DIO, ob/ob, and leptin-receptor–deficient (db/db) mice (Fig 1H–1J), and no differences were seen in the brains of these mice (S2A–S2C Fig).

MiRNA biogenesis involves the transcription of primary miRNA (pri-miRNA), the formation of precursor miRNA (pre-miRNA) in the nucleus, and the generation of mature miRNA in the cytoplasm. QRT-PCR analyses revealed that both pri- and pre-miR-26a were significantly down-regulated in islets from HFD, ob/ob, and db/db mice compared with those from control mice (Fig 1K and 1L). These results suggest that islet miR-26a is transcriptionally down-regulated during obesity, providing a potential target site for the obesity-induced reduction of miR-26a in circulating exosomes.

### Increased miR-26a in β cells improves glucose homeostasis and insulin sensitivity

**Fig 2:**
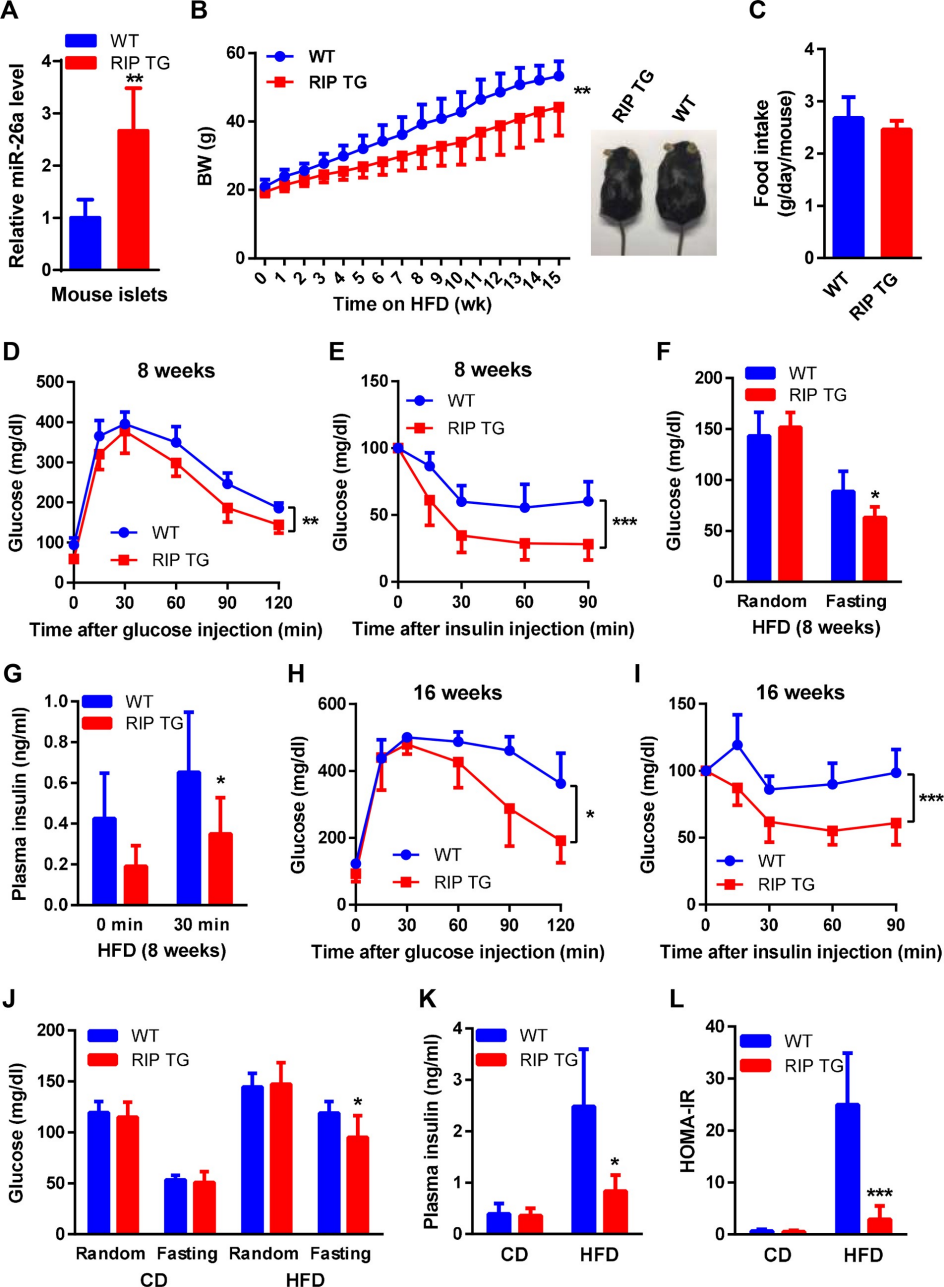
β-cell–specific overexpression of miR-26a prevents obesity-induced hyperinsulinemia and insulin resistance. (A) QRT-PCR analysis of miR-26a expression in islets of RIP TG mice and WT littermate controls (*n* = 4). (B–L) Mice were fed an HFD beginning at 6–8 weeks of age. The following measurements were performed during the course of the HFD. (B) Total BW (*n* = 8–10). Representative picture of RIP TG and WT littermate controls (right panel). (C) Food intake (*n* = 5–6). (D–G) GTT (*n* = 7–8) (D), ITT (*n* = 8–10) (E), blood glucose (*n* = 9–12) (F), and insulin levels during GTT (*n* = 7) (G), performed after 8 weeks HFD. (H and I) GTT (*n* = 5) (H) and ITT (*n* = 7–10) (I) performed after 16 weeks HFD. (J and K) Blood glucose (*n* = 8–12) (J) and insulin (*n* = 5–7) (K) levels of mice that were fed with either CD or HFD for 16 weeks. Random or fasting conditions are noted. (L) HOMA-IR (*n* = 5–9). The data underlying this figure may be found in S1 Data. Data are shown as mean ± SD. *P < 0.05, **P < 0.01, ***P < 0.005, 2-tailed ANOVA (B, D, E, H, I) and Student *t* test (C, F, G, J–L). BW, body weight; CD, chow diet; GTT, glucose tolerance test; HFD, high-fat diet; HOMA-IR, homeostatic model assessment index of insulin resistance; ITT, insulin tolerance test; QRT-PCR, quantitative reverse transcriptase PCR; RIP, rat insulin promoter; TG, transgenic; WT, wild type.

To directly study the consequence of miR-26a reduction, we generated transgenic (TG) mice expressing miR-26a under the control of the rat insulin promoter (RIP) to selectively enhance miR-26a expression in β cells. The results showed that the miR-26a levels were approximately 3-fold higher in the islets of RIP TG mice than in those of WT littermates (Fig 2A) but remained unchanged in other examined tissues (S3A Fig). The magnitude of the increase in miR-26a was comparable to that of reduced miR-26a observed in obese mice (Fig 1H–1J), suggesting that RIP TG mice are an excellent model to determine the effects of miR-26a restoration on obesity-associated metabolic changes.

RIP TG mice were born at mendelian frequencies and were morphologically indistinguishable from their WT littermates. RIP TG and WT littermates fed a chow diet (CD) exhibited similar body weight (BW), glucose tolerance, insulin sensitivity, blood glucose, blood insulin, and tissue morphology (S3B–S3H Fig). However, when 6- to 8-week–old mice were fed an HFD, RIP TG mice had significantly lower BWs than their WT littermates (Fig 2B), although both genotypes had comparable food intake (Fig 2C). Of note, mice of both genotypes displayed similar levels of miR-26a in the brain and hypothalamus (S4 Fig). When the mice were fed an HFD for either 8 or 16 weeks, RIP TG mice had significantly better glucose tolerance and insulin sensitivity than their WT littermates, as evidenced by the glucose tolerance test (GTT) and the insulin tolerance test (ITT), respectively (Fig 2D, 2E, 2H and 2I). Accordingly, RIP TG mice exhibited significantly lower fasting blood glucose and insulin levels, while no differences were detected between age-matched RIP TG mice and WT controls fed a CD (Fig 2F, 2J and 2K). During GTT analysis, insulin levels in RIP TG mice were lower than in WT littermates (Fig 2G), suggesting that improved glucose regulation in RIP TG mice resulted from improved insulin sensitivity and not increased insulin secretion. Consistent with this, the HOMA-IR was dramatically lower in RIP TG mice (Fig 2L). These results demonstrated that miR-26a restoration was sufficient to prevent obesity-induced glucose dysregulation and insulin resistance and rescued features of obesity-associated metabolic changes.

### Exosomal miR-26a increases insulin sensitivity

β cell factors have been traditionally known to improve glucose homeostasis by increasing insulin levels. Strikingly, RIP TG mice exhibited beneficial improvements in systemic metabolic homeostasis but had reduced insulin levels. Therefore, we next focused on the effects of miR-26a on both insulin sensitivity and insulin levels.

It has been shown that inhibition of hepatic insulin resistance alone can recapitulate many aspects of T2D, highlighting the significance of hepatic insulin sensitivity [31,32]. Moreover, we previously demonstrated that miR-26a improves insulin sensitivity and liver metabolism both in vitro and in vivo [22]. We therefore examined the effects of β cell miR-26a on hepatocytes.

**Fig 3:**
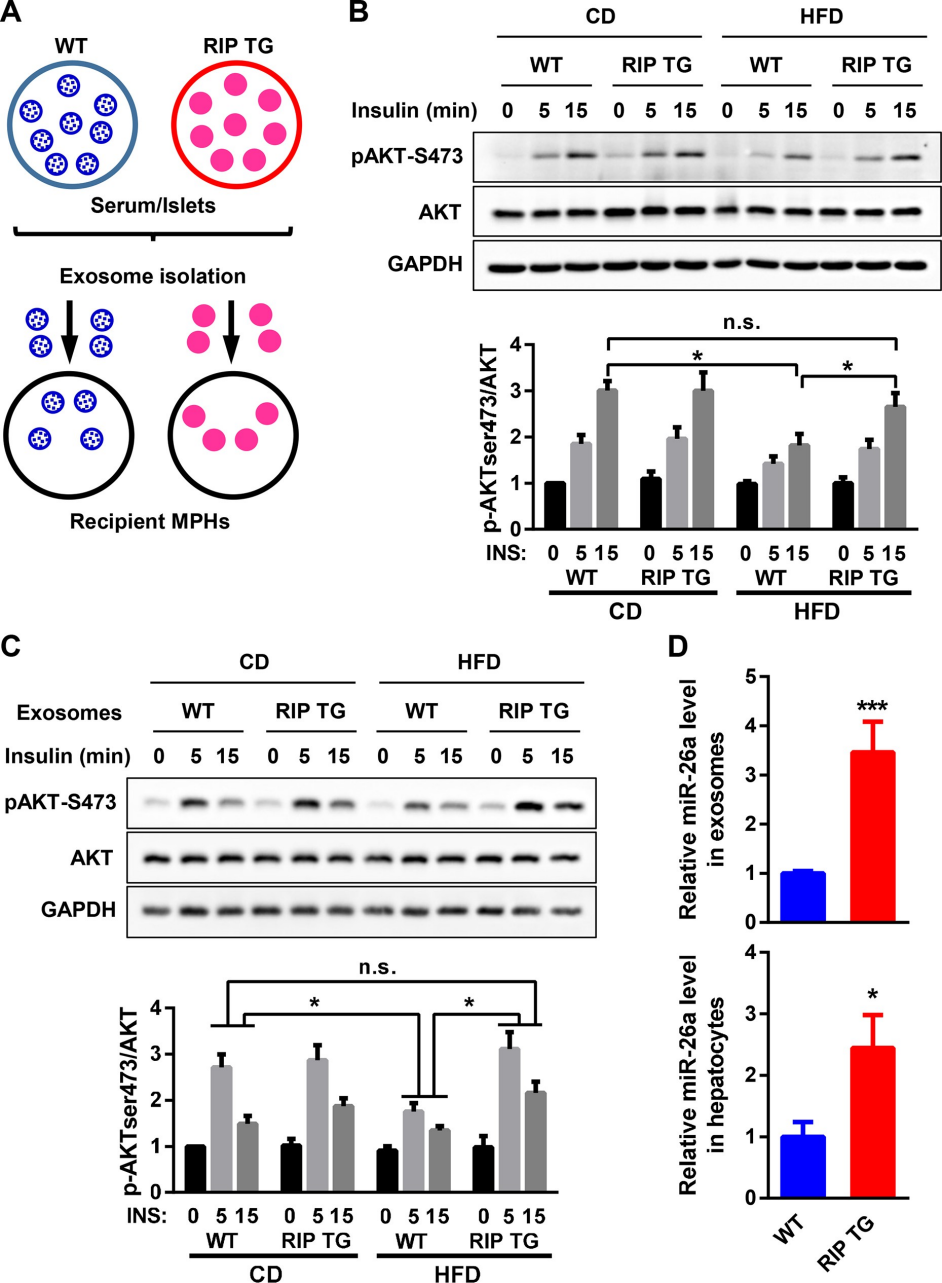
Serum exosomes from RIP TG mice enhance insulin signaling. (A) Schematic of the exosome transfer or coculture experiment. (B) AKT phosphorylation in MPHs cultured with serum from RIP TG and WT littermates fed a CD or an HFD and stimulated with insulin (10 nM) for the indicated times. (C and D) Exosomes were isolated from the islets of RIP TG or WT littermates fed a CD or an HFD and then transferred to MPHs. (C) AKT phosphorylation in MPHs given either WT or RIP TG serum exosomes and stimulated with insulin. (D) Levels of miR-26a in serum exosomes (upper panel) and MPHs (lower panel). Results are representative of 3 replicated independent experiments (B-D) and ImageJ quantification of the pAKT/AKT ratio is shown (B and C). The data underlying this figure may be found in S1 Data and S1 Raw Images. Data are shown as mean ± SD. *P < 0.05, ***P < 0.005, Student *t* test. AKT, AKT serine/threonine kinase; CD, chow diet; GAPDH, glyceraldehyde-3-phosphate dehydrogenase; HFD, high-fat diet; INS, insulin; MPHs, murine primary hepatocytes; pAKT, phosphorylated AKT; RIP, rat insulin promoter; TG, transgenic; WT, wild type.

Murine primary hepatocytes (MPHs) were cultured with serum from RIP TG mice and their WT littermates fed either a CD or an HFD (Fig 3A). Insulin responsiveness was then measured by western blotting. Insulin-stimulated AKT activation was clearly decreased in MPHs cultured with the serum of WT mice fed an HFD compared with MPHs cultured with the serum of mice fed a CD (Fig 3B). However, this decrease was blocked in MPHs cultured with the serum of RIP TG mice fed an HFD (Fig 3B). These results indicate that certain components in serum can modulate insulin sensitivity.

To clarify the putative serum component, exosomes were isolated from mouse serum and then transferred to MPHs. Insulin-stimulated AKT activation was markedly increased in MPHs after treatment with exosomes from RIP TG mice fed an HFD compared with obese WT controls, although no obvious differences were detected in either RIP TG mice or WT controls fed a CD (Fig 3C). Consistent with this, miR-26a was significantly increased in exosomes from RIP TG mice and in MPHs after treatment with exosomes from RIP TG mice (Fig 3D). These results suggest that increased levels of miR-26a in β cells lead to increased exosomal miR-26a, which might antagonize insulin resistance.

**Fig 4:**
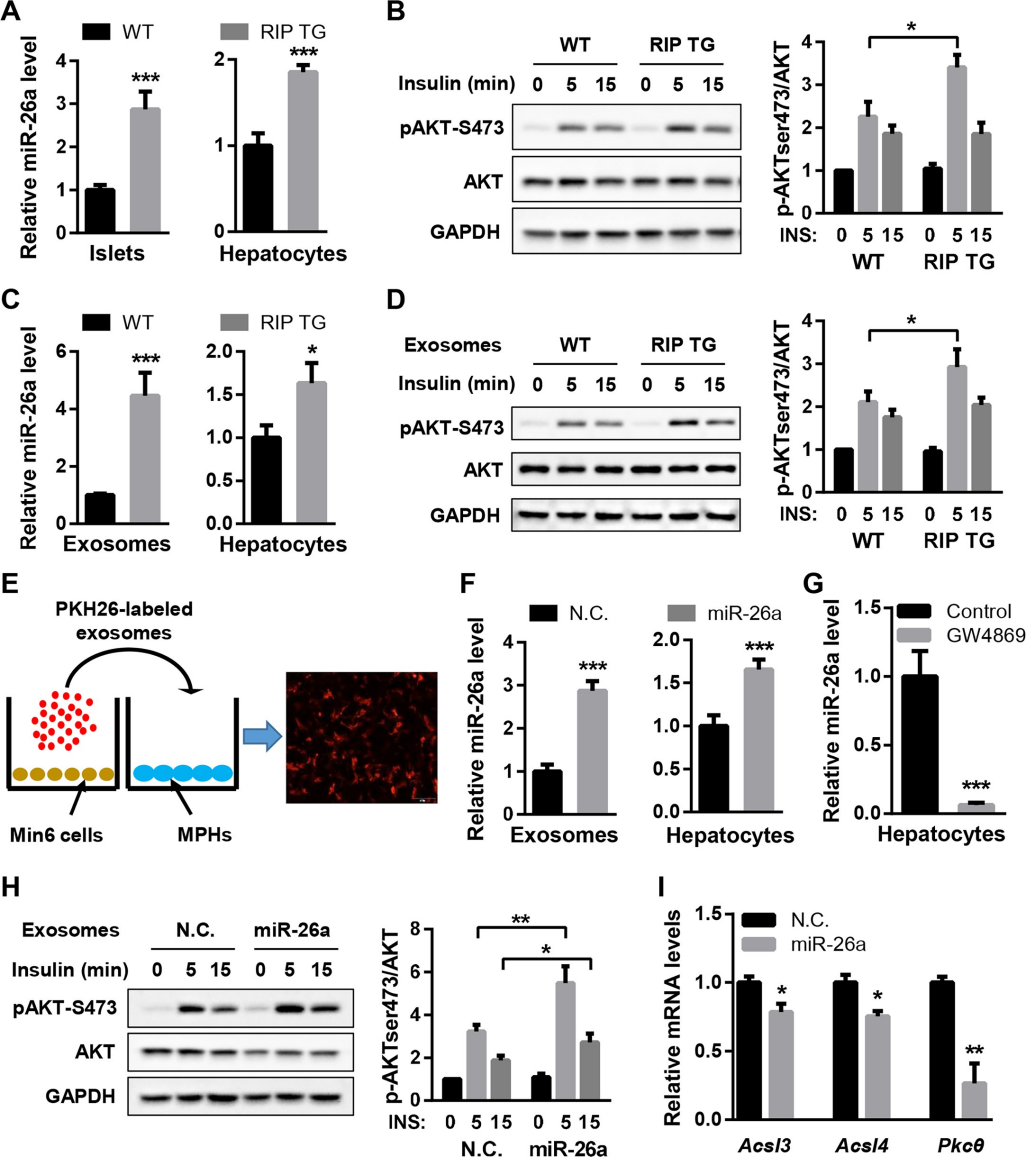
Exosomal miR-26a derived from β cells regulates insulin signaling. (A and B) Islets were isolated from either RIP TG or WT littermates fed an HFD for 16 weeks and cocultured with MPHs. (A) Levels of miR-26a in islets (left panel) and MPHs (right panel). (B) AKT phosphorylation in MPHs stimulated with insulin. (C and D) Exosomes were isolated from islets of RIP TG or WT littermates fed an HFD for 16 weeks and then transferred to MPHs. (C) Levels of miR-26a in islet exosomes (left panel) and MPHs (right panel). (D) AKT phosphorylation in MPHs stimulated with insulin. (E) Exosomes secreted from Min6 cells were labeled with PKH26 and then added to MPHs. (F–I) Exosomes were collected from media of Min6 cells transfected with miR-26a mimics (miR-26a) or NCs and then transferred to MPHs. (F) Levels of miR-26a in Min6-derived exosomes (left panel) and MPHs (right panel). (G) Levels of miR-26a in MPHs treated with exosomes from miR-26a–overexpressing Min6 cells that were treated with the exosome inhibitor GW4869 or controls. (H) AKT phosphorylation in MPHs stimulated with insulin. (I) Levels of miR-26a targets in MPHs. Results are representative of 3 replicated independent experiments (A–I), and ImageJ quantification of the pAKT/AKT ratio is shown (B, D, and H). The data underlying this figure may be found in S1 Data and S1 Raw Images. Data are shown as mean ± SD. *P < 0.05, **P < 0.01, ***P < 0.005, Student *t* test. *Acsl3/4*, acyl-CoA synthetase long chain family member 3/4; AKT, AKT serine/threonine kinase; GAPDH, glyceraldehyde-3-phosphate dehydrogenase; INS, insulin; Min6 cells, murine β cells; MPHs, murine primary hepatocytes; NC, negative control; pAKT, phosphorylated AKT; *Pkcθ*, protein kinase C theta; RIP, rat insulin promoter; TG, transgenic; WT, wild type.

We next investigated the impact of islet miR-26a on hepatic insulin sensitivity. MPHs were cocultured with islets isolated from DIO mice, followed by measurements of insulin responsiveness. MiR-26a was significantly increased in RIP TG islets and cocultured MPHs (Fig 4A), suggesting a transfer of islet miR-26a into recipient cells. Accordingly, insulin-stimulated AKT activation was markedly increased in MPHs cocultured with RIP TG islets (Fig 4B). Furthermore, we purified exosomes from the culture medium of isolated islets from DIO mice. MiR-26a levels were markedly higher in islet exosomes from RIP TG mice than in islet exosomes from their WT littermates (Fig 4C), recapitulating miR-26a levels in serum exosomes (Fig 3D). MPHs treated with islet exosomes from RIP TG mice had increased insulin-stimulated AKT activation (Fig 4D), in line with increased levels of miR-26a in recipient MPHs (Fig 4C). These results indicate that islets can secrete exosomes containing miR-26a, which was taken up by hepatocytes and subsequently modulated insulin sensitivity.

Next, we further confirmed the connection between exosomal miR-26a and insulin sensitivity by using pancreatic β cell lines. Culture medium was collected from Min6 cells (murine β cells) and purified by 0.4-μm filters, which allows for small molecules and vesicles such as exosomes to pass through. MiR-26a levels were much higher in culture medium from miR-26a–overexpressing Min6 cells than in culture medium from control cells (S5A Fig). These observations were recapitulated in INS-1 cells (rat β cells) (S5B Fig). Given that hyperglycemia is a hallmark of T2D, we determined the potential effect of glucose concentration on exosomal miR-26a release in β cells. QRT-PCR analysis found that Min6 cells cultured with high glucose concentration (16.7 mM) had reduced levels of exosomal miR-26a (S6 Fig), consistent with the observation that the levels of exosomal miR-26a were inversely correlated with fasting blood glucose levels (Fig 1D). Together, these results indicated that β cells could secrete miR-26a, whose levels were positively associated with its expression in the donor cells.

We also questioned whether β-cell–derived exosomes can be taken up by hepatocytes. To this end, Min6-derived exosomes were labeled with the fluorescent dye PKH26 and then added to the culture medium of MPHs. After 12 hours, MPHs exhibited efficient uptake of Min6-derived exosomes, as indicated by the presence of red fluorescence staining in these cells (Fig 4E). Then, we examined whether Min6-derived exosomes could modulate insulin sensitivity. The levels of miR-26a were significantly increased in exosomes derived from Min6 cells overexpressing miR-26a and recipient MPHs (Fig 4F). Prior addition (24 hours) of EV secretion inhibitor GW4869 to Min6 cells blocked exosome production and delivery of miR-26a from Min6 cells to MPHs (Fig 4G), indicating that β cells secreted extracellular miRNAs predominantly in an exosome-dependent manner. Notably, MPHs that were treated with exosomes derived from Min6 cells overexpressing miR-26a showed increased insulin-stimulated AKT activation (Fig 4H). In line with this observation, miR-26a targets acyl-CoA synthetase long chain family member (*Acsl*) *3*, *Acsl4*, and protein kinase C theta (*Pkcθ*) were down-regulated in recipient MPHs (Fig 4I). Taken together, these data demonstrate that exosomal miR-26a derived from β cells could effectively improve insulin sensitivity in recipient cells such as hepatocytes.

Certain adipose-derived exosomal miRNAs have been recently implicated in insulin resistance and metabolic diseases [29,30]. We found that the levels of serum exosomal miR-26a were comparable in adipose-specific miR-26a overexpression mice and their WT littermates fed an HFD for 16 weeks (S7 Fig), arguing against the adipose tissues as major sources for reduced circulating miR-26a in diabetic mice.

### Exosomal miR-26a regulates gene expression in peripheral tissues

**Fig 5:**
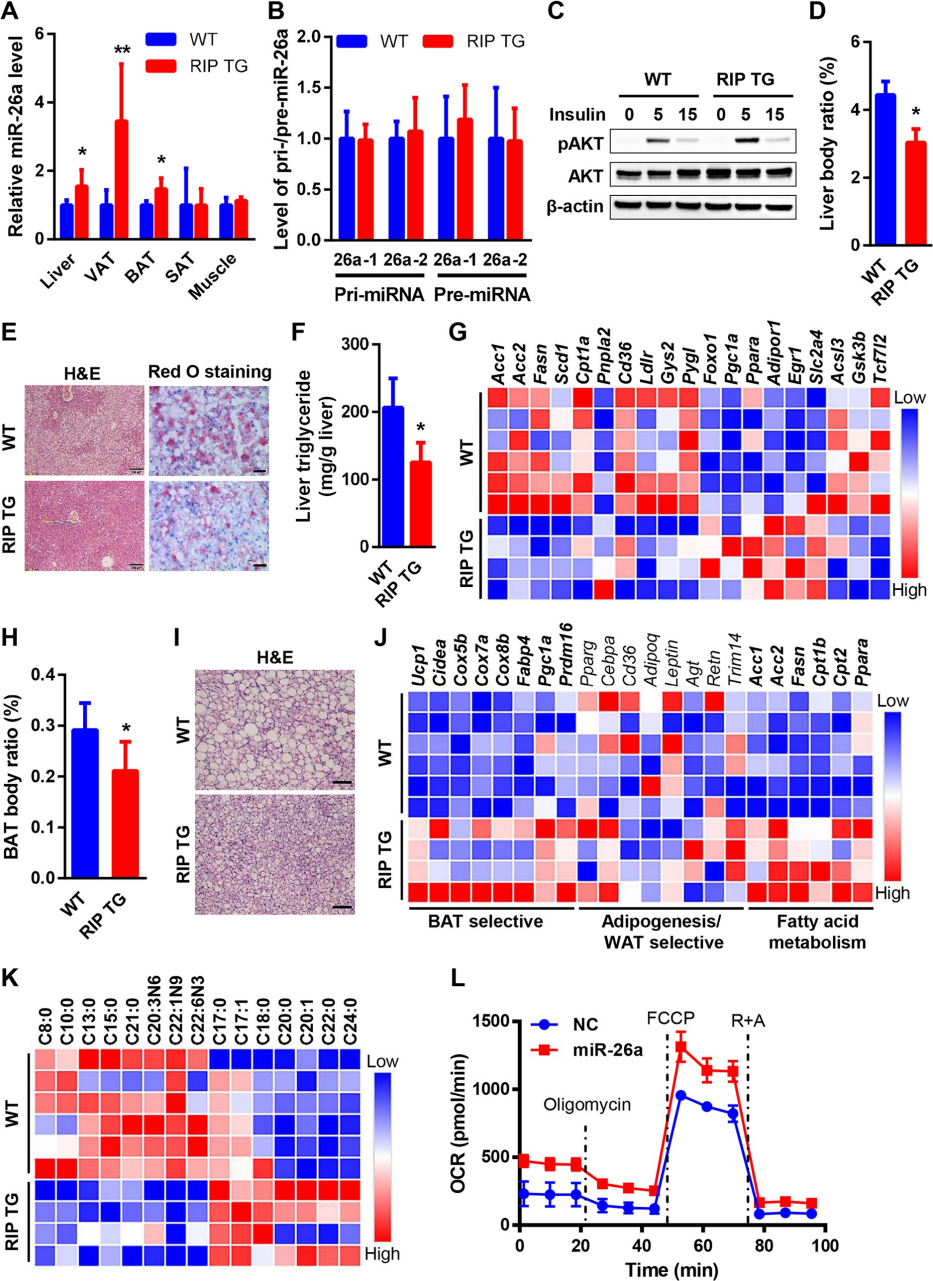
β cell miR-26a preserves the functions of peripheral tissues. (A–J) 6- to 8-week–old RIP TG and WT littermate controls were fed an HFD for 16 weeks. (A) Expression of miR-26a in obesity-associated tissues (*n* = 4–6). (B) Hepatic expression of pri- and pre-miR-26a (*n* = 4–6). (C) AKT phosphorylation in primary hepatocytes isolated from RIP TG mice and WT littermate controls and stimulated with insulin (10 nM) for the indicated times. Results are representative of 3 separate experiments. (D) Liver body ratio (*n* = 5–7). (E) Representative HE-stained and Oil-red O-stained liver (scale bar, 100 μm) (*n* = 4). (F) Hepatic triglyceride (*n* = 4–5). (G) Heat map of mRNA levels of hepatic genes involved in liver metabolism. Red and blue depict higher and lower gene expression, respectively. Color intensity indicates magnitude of expression differences. All listed genes were differentially expressed in two mouse groups with significance (*P* < 0.05) (*n* = 4–6). (H) BAT body ratio (*n* = 5–7). (I) Representative HE-stained BAT (scale bar, 50 μm) (*n* = 4). (J) Heat map of mRNA levels of adipogenesis, fatty acid metabolism, and BAT-selective and WAT-selective genes in BAT. Genes with differential expression in two mouse groups were highlighted in boldface (*P* < 0.05) (*n* = 4–6). (K) Metabolomic profiling of fatty acid metabolites in BATs with differential levels between two mouse groups (*n* = 4–6). (L) OCR for primary brown adipocytes isolated from WT and transfected with NCs or miR-26a mimics. Oligomycin, FCCP, and rotenone and antimycin were added at indicated time points. The data underlying this figure may be found in S1 Data and S1 Raw Images. Data are shown as mean ± SD. *P < 0.05, **P < 0.01, Student *t* test. *Acc*, acetyl-CoA carboxylase; *Acsl*, acyl-CoA synthetase long chain; *Adipoq*, adiponectin precursor; *Adipor1*, adiponectin receptor 1; *Agt*, angiotensinogen; AKT, AKT serine/threonine kinase; BAT, brown adipose tissue; *Cd36*, CD36 molecule; *Cebpa*, CCAAT/enhancer binding protein (C/EBP) alpha; *Cidea*, cell death inducing DFFA like effector A; *Cox*, cytochrome c oxidase; *Cpt1a*, carnitine palmitoyltransferase 1A; *Egr1*, early growth response protein 1; *Fabp4*, fatty acid binding protein 4, adipocyte; *Fasn*, fatty acid synthase; FCCP, carbonyl cyanide 4-(trifluoromethoxy) phenylhydrazone; *Gsk3b*, glycogen synthase kinase 3 beta; *Gys2*, glycogen synthase 2; HE, hematoxylin–eosin; HFD, high-fat diet; *Ldlr*, low-density lipoprotein receptor; NC, negative control; OCR, oxygen consumption rate; *Pgc1a*, peroxisome proliferator-activated receptor gamma coactivator 1-alpha; *Pnpla2*, patatin-like phospholipase domain-containing protein 2; *Ppara*, peroxisome proliferator activated receptor alpha; *Pparg*, peroxisome proliferator activated receptor gamma; *Prdm16*, PR domain containing 16; pre-miR-26a, precursor miR-26a; pri-miR-26a, primary miR-26a; *Pygl*, glycogen phosphorylase L; *Retn*, resistin; RIP, rat insulin promoter; SAT, subcutaneous adipose tissue; *Scd1*, stearoyl-CoA desaturase; *Tcf7l2*, transcription factor 7 like 2; TG, transgenic; *Trim14*, tripartite motif-containing 14; *Ucp1*, uncoupling protein 1; VAT, visceral adipose tissue; WAT, white adipose tissue; WT, wild type.

Given that circulating exosomal miR-26a is reduced during obesity and can modulate insulin sensitivity in vitro, we evaluated its pathophysiological implications in vivo. On the basis of increased circulating exosomal miR-26a in RIP TG mice, we anticipated that miR-26a would be increased in recipient tissues. Indeed, the levels of miR-26a were specifically increased in the liver tissue, visceral adipose tissue (VAT), and brown adipose tissue (BAT) of RIP TG mice (Fig 5A), whereas levels of pri- and pre-miR-26a were unchanged (Fig 5B, S8A and S8B Fig), suggesting that this increase did not result from endogenous miR-26a up-regulation. Together, these data indicate that circulating exosomal miRNAs from β cells could be taken up in vivo by certain peripheral tissues, including liver tissue, VAT, and BAT.

Next, we investigated the outcomes of exosomal miR-26a in recipient tissues. We have previously showed that hepatic miR-26a directly targets several key regulators involved in fatty acid synthesis (ACSL3, ACSL4), gluconeogenesis (transcription factor 7 like 2 [TCF7L2], phosphoenolpyruvate carboxykinase 1 [PCK1]), and insulin signaling (glycogen synthase kinase 3 beta [GSK3β], PKCδ, PKCθ), thereby modulating insulin sensitivity and metabolism of glucose and lipids [22]. Therefore, we initially determined the effects of exosomal miR-26a on hepatic insulin sensitivity and liver metabolism. In the liver, insulin-stimulated AKT activation was remarkably increased in RIP TG mice (Fig 5C), consistent with the effect of endogenous miR-26a on hepatic insulin sensitivity [22]. RIP TG mice had lower liver weight (Fig 5D), less hepatic steatosis and less lipid accumulation than WT littermates (Fig 5E). Hepatic triglyceride levels (Fig 5F), plasma cholesterol, and low-density lipoprotein (LDL) and high-density lipoprotein (HDL) levels were also lower in RIP TG mice than in WT littermates (S8C–S8E Fig). To explore the underlying mechanism, we examined the expression of 30 genes critical for liver metabolism and function (Fig 5G, S8F Fig). A number of genes that control fatty acid synthesis (acetyl-CoA carboxylase [*Acc*] *1*, *Acc2*, fatty acid synthase [*Fasn*], and stearoyl-CoA desaturase [*Scd1*]), cholesterol metabolism (*Cd36* and LDL receptor [*Ldlr*]), and glucogen metabolism (glycogen synthase 2 [*Gys2*] and glycogen phosphorylase L [*Pygl*]) were significantly down-regulated in RIP TG mice (Fig 5G). The metabolic regulators *Foxo1*, peroxisome proliferator-activated receptor gamma coactivator 1-alpha (*Pgc1a*), and peroxisome proliferator activated receptor alpha (*Ppara*) were also significantly up-regulated in RIP TG mice. Notably, miR-26a targets *Acsl3*, *Gsk3b*, and *Tcf7l2* were down-regulated in RIP TG mice, suggesting that exosomal miR-26a could modulate endogenous targets in recipient cells.

RIP TG mice had reduced BAT weight (Fig 5H), decreased adipocyte size, and reduced abundance of lipid droplets (Fig 5I). Expression analyses revealed that RIP TG mice expressed higher levels of BAT-specific genes, including uncoupling protein 1 (*Ucp1*), fatty acid binding protein 4 (*Fabp4*), cell death inducing DFFA like effector A (*Cidea*), cytocrhome c oxidase (*Cox*) *5b*, *Cox7a*, *Cox8b*, PR domain containing 16 (*Prdm16*), and *Pgc1α* (Fig 5J). However, the levels of the pan-adipocyte and white adipose tissue (WAT)-selective genes were indistinguishable between the two genotypes. We then determined the mechanism underlying the reduced lipid accumulation in RIP TG mice. Unexpectedly, the lipogenic markers *Acc1*, *Acc2*, and *Fasn* were increased in RIP TG mice (Fig 5J), indicative of up-regulated lipogenesis. Critical regulators promoting fatty acid oxidation, including carnitine palmitoyltransferase (*Cpt*) *1b*, *Cpt2*, and *Ppara*, were also significantly increased in RIP TG mice. Overall, these results suggest that a shift towards lipolysis might be responsible for the reduced lipid accumulation in RIP TG mice. Furthermore, BAT was analyzed using gas chromatography–mass spectrometry (GC-MS)-based lipidomic analysis. A total of 15 fatty acid metabolites were significantly changed in mice of both genotypes (Fig 5K, S2 Table), further supporting a role of miR-26a in fatty acid metabolism. We also examined the cell autonomous effects of miR-26a on BAT mitochondria respiration by measuring oxygen consumption rate (OCR). This analysis showed that OCR was increased in cultured primary brown adipocytes overexpressing miR-26a (Fig 5L, S8G Fig). Together, these data indicate that exosomal miR-26a might preserve BAT functions and alleviate obesity-induced metabolic dysregulation.

In VAT, hematoxylin–eosin (HE) analyses revealed that RIP TG mice had smaller adipocytes than their WT littermates (S8H Fig), suggesting reduced adipocyte hypertrophy and improved insulin sensitivity. To determine the underlying molecular mechanisms, we examined the expression of 20 key metabolic, inflammatory, and insulin target genes (S8I and S8J Fig). In line with the lean phenotype, we observed a gene expression pattern associated with energy expenditure and lipid mobilization in RIP TG mice, including increased expression of *Ucp3*, patatin-like phospholipase domain-containing 2 (*Pnpla2*), *Pgc1a*, and peroxisome proliferator activated receptor gamma (*Pparg*). Notably, the miR-26a target ADAM metallopeptidase domain 17 (*Adam17*) was significantly down-regulated in RIP TG mice (S8I Fig). Chronic inflammation in white fat is associated with obesity and insulin resistance [33]. However, no marked differences were detected in the expression of inflammation markers, including interleukin 6 (*Il6*), tumor necrosis factor alpha (*Tnfα*), and EGF-Like module receptor 1 (*Emr1*) (S8J Fig). Of note, these results suggest a role of miR-26a in lipid metabolism in both liver and adipose tissues. Although several miR-26a targets have been implicated in hepatic lipid metabolism [22], it remains unknown whether these targets or distinct molecules are responsible for the function of miR-26a in adipose lipid metabolism, which will be interesting for future investigation.

Taken together, these results further suggest that circulating exosomal miR-26a from β cells could be taken up by peripheral tissues and subsequently modulate the metabolism and function of recipient cells in vivo.

### MiR-26a reduces GSIS

**Fig 6:**
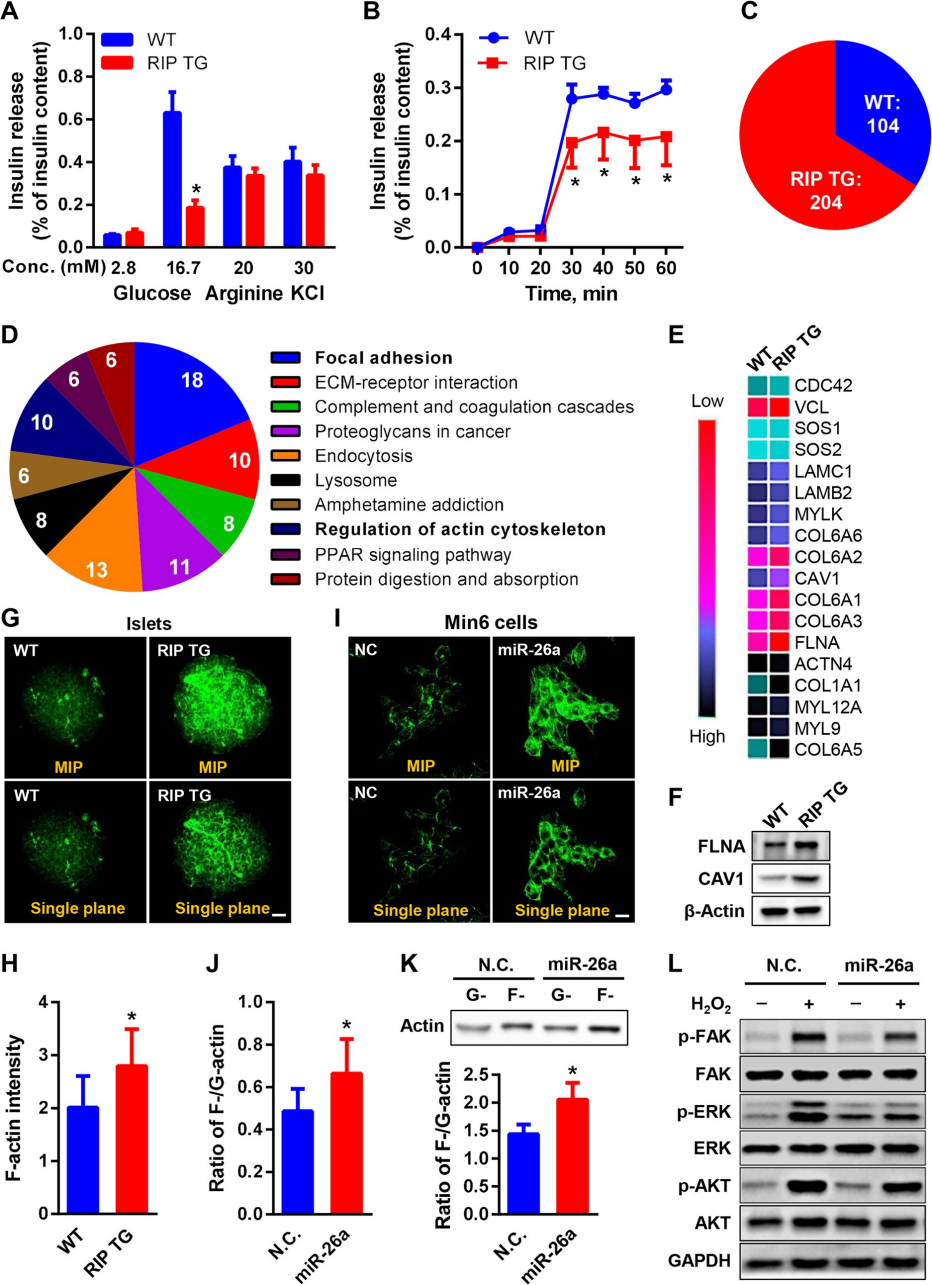
MiR-26a regulates insulin secretion through modulating actin remodeling. (A) Static insulin secretion performed with islets from mice fed an HFD for 12 weeks at indicated glucose, arginine, and KCl concentrations (*n* = 3). (B) Time course of GSIS in islets from mice fed an HFD for 14 weeks (*n* = 4). (C–E) Proteomic analysis on islets of either RIP TG or WT littermate controls fed an HFD for 2 days. (C) Venn diagram comparing islet proteins. The number of proteins that are differentially expressed are shown. RIP TG mice showed an increase of 204 proteins and a decrease of 104 proteins. (D) KEGG pathway analysis on islet proteins deregulated in RIP TG mice. (E) Heat map of protein levels of genes involved in focal adhesion. (F) The protein levels of FLNA and CAV1 in the islets were verified by western blotting. (G and H) Representative IF imaging of F-actin in isolated islets treated with glucose (scale bar, 20 μm) (G). Quantification of F-actin intensity are shown (H) (*n* = 9 islets from 3 independent pancreatic samples per genotype). (I–L) Min6 cells were transfected with either miR-26a mimics (miR-26a) or NCs, followed by H_2_O_2_ treatment (*n* = 3). (I) Representative IF imaging of F-actin (scale bar, 20 μm). (J) Ratio of G-actin and F-actin fluorescence intensity. (K) Protein levels of F-actin and G-actin. (L) The activation kinetics of focal adhesion. The data underlying this figure may be found in S1 Data and S1 Raw Images. Data are shown as mean ± SD. *P < 0.05, Student *t* test. ACTN4, actinin alpha 4; AKT, AKT serine/threonine kinase; CAV1, caveolin 1; CDC42, cell division cycle 42; COL6A, collagen type VI alpha; ECM, extracellular matrix; ERK, extracellular signal-regulated kinase; FAK, focal adhesion kinase; FLNA, filamin alpha; F-actin, filamentous actin; GAPDH, glyceraldehyde-3-phosphate dehydrogenase; GSIS, glucose-stimulated insulin secretion; G-actin, globular actin; HFD, high-fat diet; IF, immunofluorescence; KEGG, Kyoto Encyclopedia of Genes and Genomes; LAMB2, laminin subunit beta 2; LAMC1, laminin subunit gamma 1; Min6 cells, murine β cells; MIP, maximum intensity projection; MYLK, myosin light chain kinase; NC, negative control; pAKT, phosphorylated AKT; p-ERK, phosphorylated ERK; p-FAK, phosphorylated FAK; PPAR, peroxisome proliferator activated receptor; RIP, rat insulin promoter; TG, transgenic; SOS, SOS Ras/Rac guanine nucleotide exchange factor; VCL, vinculin; WT, wild type.

Serum insulin levels are controlled by a combination of the number of β cells and the insulin secretory capacity of individual β cells. We first determined the effect of miR-26a restoration on insulin secretion. To this end, mouse islets were isolated and treated with different insulin secretagogues. Basal insulin secretion was not affected, but when evoked by 16.7 mM glucose, insulin secretion decreased approximately 3-fold in the islets of RIP TG mice (Fig 6A). Insulin content was increased in the islets of RIP TG mice compared with WT controls (S9A Fig), consistent with a promotive role of miR-26a in insulin transcription as described previously [21]. In contrast, insulin secretion induced by either arginine or KCl was similar in both genotypes. Glucose is used to evoke both first- and second-phase insulin release, while KCl is regarded to induce the first phase of insulin secretion. Our results thus suggested that miR-26a might specifically regulate the second phase of GSIS. To confirm this, we compared the kinetics of insulin secretion in glucose-stimulated mouse islets. Indeed, extensive decreases were detected in the islets of RIP TG mice in the second phase of GSIS, although there was no discernible difference in first-phase insulin secretion (<20 min) between genotypes (Fig 6B). Recent evidence suggest insulin signaling in β cells as an emerging regulator of insulin secretion [34–36]. To test whether this pathway participants in the function of miR-26a on insulin secretion, we determined the effect of miR-26a in insulin signaling of β cells and islets. Overexpression of miR-26a in INS-1 cells had minor effect on insulin-stimulated AKT activation (S9B Fig). Furthermore, the levels of insulin-stimulated AKT activation were comparable in the islets from both RIP TG mice and their WT littermates fed an HFD (S9C Fig), indicating that miR-26a might not modulate β cell insulin sensitivity.

To elucidate the underlying mechanism, we performed a large-scale proteomic analysis using quantitative MS. Total proteins were extracted from the islets of WT and RIP TG mice fed an HFD for 2 days; the proteins were cleaved into peptides with trypsin and analyzed by ultraperformance liquid chromatography–electrospray tandem MS (UPLC-ESI-MS/MS). These experiments identified 4,104 proteins, of which 308 were differentially expressed between WT and RIP TG mice (>1.3-fold) (S3 Table). A total of 204 proteins were up-regulated, whereas 104 proteins were down-regulated in the islets of RIP TG mice compared with the islets of their WT littermates (Fig 6C). Gene ontology analyses of the 308 differentially expressed proteins suggested that β cell miR-26a was associated with decreased GSIS, extracellular exosomes, and focal adhesion (S9D and S9E Fig). Kyoto Encyclopedia of Genes and Genomes (KEGG) pathway analyses further showed that focal adhesion was ranked as the most significant dysregulated pathway (Fig 6D), with 18 proteins exhibiting dysregulated expression (Fig 6E). These effects on focal adhesion proteins were further verified by QRT-PCR (S9F Fig) and western blotting (Fig 6F). The pathway “regulation of actin cytoskeleton” was also significantly dysregulated (Fig 6D), with 10 proteins exhibited dysregulated expression in RIP TG mice.

Focal adhesion and actin cytoskeleton remodeling are closely associated and play principal regulatory roles in the second phase of GSIS [37,38]. We thus investigated the role of miR-26a in actin remodeling. Immunofluorescence (IF) staining of whole islets treated with glucose showed marked differences in F-actin cytoskeleton organization: the islets of WT littermates exhibited a thin cortical actin network and fine-mesh structures, while islets of RIP TG mice had higher levels of F-actin fluorescence intensity and increased density of F-actin meshwork (Fig 6G and 6H).

For further confirmation, Min6 cells were transfected with miR-26a mimics or negative controls, followed by treatment with exogenous H_2_O_2_ (100 μM for 30 min), which mimics glucose signaling and increases insulin secretion. As expected, Min6 cells overexpressing miR-26a had increased F-actin intensity and dense F-actin meshwork (Fig 6I). Moreover, the ratio between F-actin and monomeric globular actin (G-actin) was markedly increased by miR-26a overexpression, as evidenced by co-IF (Fig 6J, S9G Fig) and western blotting (Fig 6K).

Disruption of the dynamic equilibrium state of actin polymerization can blunt the activation of focal adhesion signaling [38,39]. We thus examined the effect of miR-26a on H_2_O_2_-induced activation of focal adhesion kinase (FAK) and its downstream signaling effectors, extracellular signal-regulated kinase (ERK) and AKT. The results showed that phosphorylation of FAK and ERK1/2 were clearly reduced in miR-26a–overexpressing Min6 cells (Fig 6L). Together, these results indicate that miR-26a impairs GSIS by impeding actin cytoskeleton remodeling.

### MiR-26a prevents obesity-induced islet hyperplasia

**Fig 7:**
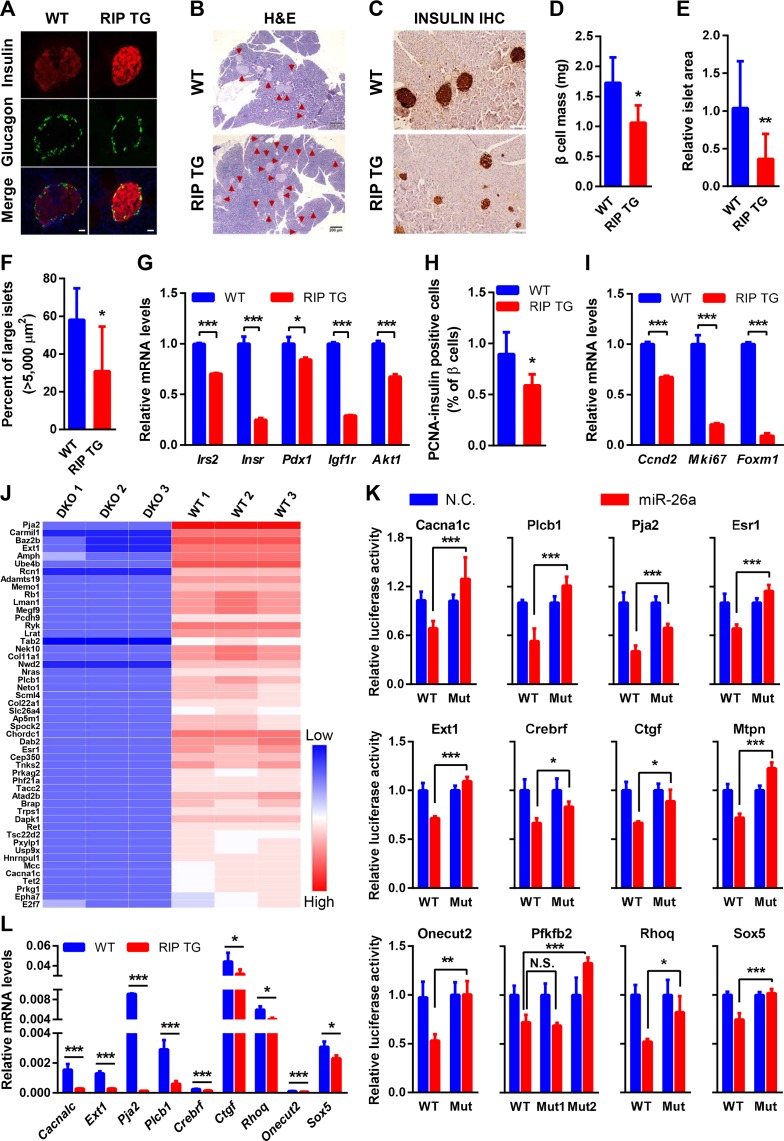
MiR-26a inhibits obesity-induced hyperplasia and targets several genes critical for β cell proliferation and insulin secretion. (A) Representative IF staining for insulin and glucagon in pancreatic islets (scale bar, 200 μm) (*n* = 4). (B) Representative HE-stained pancreas (scale bar, 200 μm) (*n* = 4). (C) Representative IHC staining for insulin in pancreatic islets (scale bar, 100 μm) (*n* = 4). (D) β cell mass (*n* = 4). (E) Islet area relative to pancreas area (*n* = 8–10). (F) Percentage of large islets (*n* = 5–8). (G) mRNA levels of the markers for β cell hyperplasia (*n* = 3). (H) Quantification of IF staining for insulin and PCNA in pancreas (*n* = 4). (I) mRNA levels of the markers for β cell replication (*n* = 3). (J) The top 50 enriched genes in the islet of WT mouse identified by Ago2 RNA immunoprecipitation sequencing are presented in a heat map (*n* = 3). Red and blue depict higher and lower gene enrichment, respectively. Color intensity indicates magnitude of enrichment differences. (K) Relative luciferase activity in 293T cells transfected with reporter constructs containing the 3′ UTR of target genes and co-transfected with either miR-26a mimics (miR-26a) or NCs. (L) Expression of miR-26a target genes in islets of RIP TG and WT littermates fed an HFD for 16 weeks (*n* = 3–4). The data underlying this figure may be found in S1 Data. Data are shown as mean ± SD. *P < 0.05, **P < 0.01, ***P < 0.005, Student *t* test. Adamts19, ADAM metallopeptidase with thrombospondin type 1 motif 19; Ago2, argonaute RISC catalytic component 2; AKT, AKT serine/threonine kinase; Amph, amphiphysin; Ap5m1, adaptor related protein complex 5 subunit Mu 1; Atad2b, ATPase family AAA domain containing 2B; Baz2b, bromodomain adjacent to zinc finger domain 2B; Brap, BRCA1 associated protein; Cacna1c, calcium voltage-gated channel subunit alpha1 C; Carmil1, capping protein regulator and myosin 1 linker 1; *Ccnd2*, cyclin D2; Cep350, centrosomal protein 350; Chordc1, cysteine and histidine rich domain containing 1; Col11a1, collagen type XI alpha 1 chain; Col22a1, collagen type XXII alpha 1 chain; Crebrf, CREB3 regulatory factor; Ctgf, connective tissue growth factor; Dab2, DAB adaptor protein 2; Dapk1, death associated protein kinase 1; DKO, double knockout; E2f7, E2F transcription factor 7; Epha7, EPH receptor A7; Esr1, estrogen receptor 1; Ext1, exostosin glycosyltransferase 1; *Foxm1*, forkhead box M 1; HE, hematoxylin–eosin; HFD, high-fat diet; Hnrnpul1, heterogeneous nuclear ribonucleoprotein U like 1; IF, immunofluorescence; *Igf1r*, insulin like growth factor 1 receptor; IHC, immunohistochemistry; *Insr*, insulin receptor; *Irs2*, insulin receptor substrate 2; Lman1, lecti mannose binding 1; Lrat, lecithin retinol acyltransferase; Mcc, MCC regulator of WNT signaling pathway; Megf9, multiple EGF like domains 9; Memo1, mediator of cell motility 1; *Mki67*, marker of proliferation Ki-67; Mtpn, myotrophin; Mut, mutant; NC, negative control; Nek10, NIMA related kinase 10; Neto1, neuropilin and tolloid like 1; Nras, neuroblastoma RAS viral oncogene; Nwd2, NACHT and WD repeat domain containing 2; Onecut2, one cut homeobox 2; Pcdh9, protocadherin 9; PCNA, proliferative cell nuclear antigen; *Pdx1*, pancreatic and duodenal homeobox 1; Pfkfb2, 6-phosphofructo-2-kinase/fructose-2,6-biphosphatase 2; Phf21a, PHD finger protein 21A; Pja2, praja ring finger ubiquitin ligase 2; Plcb1, phospholipase C beta 1; Prkag2, protein kinase AMP-activated non-catalytic subunit gamma 2; Prkg1, protein kinase CGMP-dependent 1; Pxylp1, 2-phosphoxylose phosphatase 1; Rb1, RB transcriptional corepressor 1; Rcn1, reticulocalbin 1; Ret, Ret proto-oncogene; Rhoq, Ras homolog family member Q; RIP, rat insulin promoter; Ryk, receptor like tyrosine kinase; Scml4, scm polycomb group protein like 4; Slc26a4, solute carrier family 26 member 4; Sox5, SRY-box transcription factor 5; Spock2, SPARC/Osteonectin, CWCV and Kazal like domains proteoglycan 2; Tab2, TGF-beta activated kinase 1 binding protein 2; Tacc2, transforming acidic coiled-coil containing protein 2; Tet2, tet methylcytosine dioxygenase 2; TG, transgenic; Tnks2, tankyrase 2; Trps1, transcriptional repressor GATA binding 1; Tsc22d2, TSC22 domain family member 2; Ube4b, ubiquitination factor E4B; Usp9x, ubiquitin specific peptidase 9 X-linked; WT, wild type.

We next analyzed the effect of miR-26a restoration on β cell mass. Inspection of the islet architecture of RIP TG mice revealed intact endocrine cell organization and similar β and α cell distributions as in their WT littermates (Fig 7A). HE and immunohistochemistry (IHC) for insulin showed that HFD-induced islet hyperplasia was markedly reduced in RIP TG mice (Fig 7B and 7C). β cell mass (Fig 7D), islet area (Fig 7E), and the number of large islets (Fig 7F) were significantly reduced in RIP TG mice. β cell hyperplasia activators, including *Irs-2*, insulin receptor (*Insr*), pancreatic and duodenal homeobox 1 (*Pdx1*), insulin like growth factor 1 receptor (*Igf1r*), and *Akt1*, were reduced in islets of RIP TG mice (Fig 7G), further confirming the inhibition of miR-26a on islet hyperplasia.

Obesity-induced hyperplasia is mainly due to increased β cell replication. We thus determined β cell proliferation on the basis of proliferative cell nuclear antigen (PCNA) staining. We found fewer insulin and PCNA double-positive cells in RIP TG mice than in WT controls (Fig 7H, S10 Fig). In line with reduced β cell replication, the islets of RIP TG mice exhibited decreased levels of cyclin D2 (*Ccnd2*), marker of proliferation Ki-67 (*Mki67*), and forkhead box M 1 (*Foxm1*) (Fig 7I), which are critical cell cycle regulators for β cell proliferation [40]. Taken together, these results suggest that miR-26a restoration could prevent obesity-induced hyperplasia by inhibiting the compensatory proliferation of β cells.

### MiR-26a targets genes involved in insulin secretion and β cell replication

We next identified the endogenous targets of miR-26a in β cells. The mouse and human genomes harbor two distinct miR-26a loci, miR-26a-1 and miR-26a-2. Mature miR-26-1 and miR-26-2 are identical, and this redundancy may impede a full understanding of their function in vivo. To this end, we generated mice lacking either miR-26a-1 or miR-26a-2 (S11A Fig). By intercrossing these two separate mouse lines, we obtained miR-26a double knockout (26a DKO) mice. As expected, the expression of miR-26a was completely abolished in DKO mice (S11B Fig). We performed a comprehensive Ago2 RNA immunoprecipitation sequencing in the islets from RIP TG mice and their littermates fed an HFD for 3 days, followed by unbiased miR-26a targeting seed enrichment analysis (Fig 7J, S4 Table). Additionally, we performed gene ontology and biological association analyses using DAVID Bioinformatics Resources to select specific genes associated with insulin secretion and β cell proliferation. We used miRNA-target prediction algorithms (TargetScan, PicTar and StarBase) to find subsets of genes possessing miR-26a binding sites within their 3′-UTR. These two integrative analyses collectively identified 12 genes as potential targets of miR-26a. Among them were critical regulators of insulin secretion and β cell proliferation/survival, including calcium voltage-gated channel subunit alpha1 C (*Cacna1c*), CREB3 regulatory factor (*Crebrf*), connective tissue growth factor (*Ctgf*), estrogen receptor 1 (*Esr1*), exostosin glycosyltransferase 1 (*Ext1*), myotrophin (*Mtpn*), one cut homeobox 2 (*Onecut2*), 6-phosphofructo-2-kinase/fructose-2,6-biphosphatase 2 (*Pfkfb2*), praja ring finger ubiquitin ligase 2 (*Pja2*), phospholipase C beta 1 (*Plcb1*), Ras homolog family member Q (*Rhoq*), and SRY-box transcription factor 5 (*Sox5*) (S12 Fig).

We undertook experimental validations to confirm the regulation of miR-26a on these genes. We generated luciferase constructs harboring WT or mutated miR-26a binding sites and found that miR-26a significantly repressed the luciferase activity of all 12 WT constructs (Fig 7K). Mutation of miR-26a binding sites in selected targets abolished the miR-26a–mediated repression in luciferase activity (Fig 7K), indicating a direct interaction of miR-26a with these sites. We then determined the effect of miR-26a on endogenous expression of these targets. QRT-PCR analysis revealed that the expression of miR-26a targets was significantly lower in RIP TG mice than in their WT littermates (Fig 7L). These results demonstrated that miR-26a directly regulated several regulators involved in insulin secretion and β cell proliferation.

### Deficiency of miR-26a aggravates insulin resistance and β cell dysfunction

**Fig 8:**
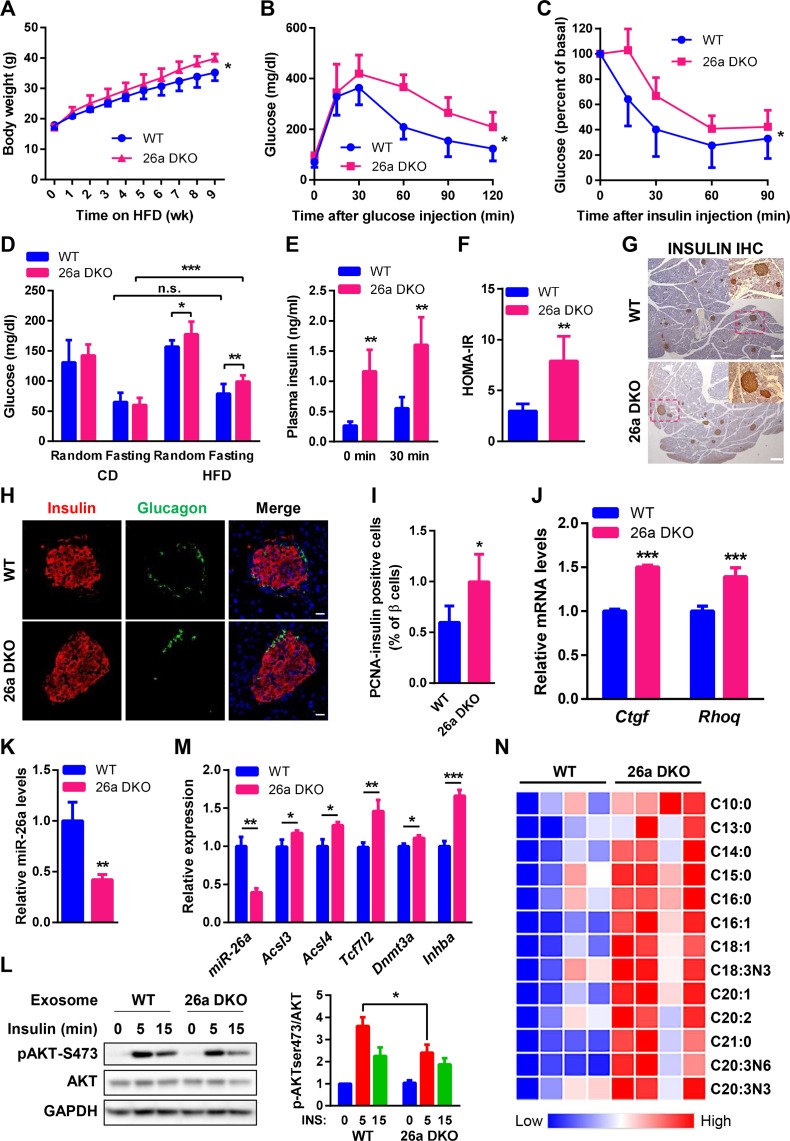
Deficiency of miR-26a aggravates obesity-induced glucose intolerance and insulin resistance. (A–J) Mice were fed an HFD beginning at 6–8 weeks of age. The following measurements were performed during the course of the HFD. (A) Total BW (*n* = 7). (B) GTT performed after 8 weeks of HFD (*n* = 78). (C) ITT performed after 9 weeks of HFD (*n* = 8). (D and E) Blood glucose (*n* = 8–12) (D) and insulin (*n* = 4–6) (E) levels of mice that were fed with either CD or HFD for 8 weeks. Random or fasting conditions are noted. (F) HOMA-IR (*n* = 5–6). (G) Representative IHC for insulin in pancreatic islets (scale bar, 200 μm) (*n* = 3). (H) Representative IF staining for insulin and glucagon in pancreatic islets (scale bar, 20 μm) (*n* = 3). (I) Quantification of IF staining for insulin and PCNA in pancreas (*n* = 4). (J) Expression of miR-26a target genes in islets (*n* = 3). (K–M) Exosomes were isolated from islets of 26a DKO mice or WT controls and then transferred into MPHs. (K) Levels of miR-26a in islet exosomes (*n* = 3). (L) AKT phosphorylation in hepatocytes stimulated with insulin. Results are representative of 3 replicated independent experiments and ImageJ quantification of the pAKT/AKT ratio is shown. (M) Levels of miR-26a and its target genes in recipient hepatocytes. (N) Metabolomic profiling of fatty acids in hepatocytes isolated from 26a DKO and WT mice fed an HFD for 3 days (*n* = 4). Red and blue depict higher and lower metabolites enrichment, respectively. Color intensity indicates magnitude of enrichment differences. The data underlying this figure may be found in S1 Data and S1 Raw Images. Data are shown as mean ± SD. *P < 0.05, **P < 0.01, ***P < 0.005, 2-tailed ANOVA (B–D) and Student *t* test (E–G, J–N). *Acsl*, acyl-CoA synthetase long chain; AKT, AKT serine/threonine kinase; BW, body weight; CD, chow diet; *Ctgf*, connective tissue growth factor; *Dnmt3a*, DNA methyltransferase 3 alpha; GAPDH, glyceraldehyde-3-phosphate dehydrogenase; GTT, glucose tolerance test; HFD, high-fat diet; HOMA-IR, homeostatic model assessment index of insulin resistance; IF, immunofluorescence; IHC, immunohistochemistry; *Inhba*, inhibin subunit beta A; ITT, insulin tolerance test; MPHs, murine primary hepatocytes; n.s., not significant; pAkt, phosphorylated AKT; PCNA, proliferative cell nuclear antigen; *Rhoq*, Ras homolog family member Q; *Tcf7l2*, transcription factor 7 like 2; WT, wild type; 26a DKO mice, miR-26a double knockout mice.

We finally confirmed our findings using a new miR-26a–deficient mouse line. When 6- to 8-week–old mice were fed an HFD, 26a DKO mice exhibited higher BWs than WT controls (Fig 8A). After 8 weeks of HFD feeding, 26a DKO mice had reduced glucose tolerance and insulin sensitivity, as indicated by GTT and ITT analysis, respectively (Fig 8B and 8C). 26a DKO mice fed an HFD had increased random and fasting blood glucose levels compared with WT controls, although no differences were detected in mice with either genotype fed a CD (Fig 8D). Moreover, 26a DKO mice had higher fasting insulin levels and increased insulin levels during GTT (Fig 8E), indicating that miR-26a deficiency may increase insulin secretion. Accordingly, HOMA-IR was remarkably increased in 26a DKO mice (Fig 8F).

IHC for insulin showed that 26a DKO mice had fewer but larger islets than WT controls (Fig 8G), indicative of increased islet hyperplasia. The distribution of α cells appeared abnormal in 26a DKO mice, as indicated by the observation that glucagon-positive cells did not mantle the insulin-positive cells in the core (Fig 8H). Moreover, PCNA immunostaining revealed more insulin and PCNA double-positive cells in 26a DKO mice than in WT controls, suggesting an increase in β cell proliferation (Fig 8I, S13 Fig). In addition, miR-26a target genes *Ctgf* and *Rhoq* were increased in 26a DKO mice compared with WT controls (Fig 8J).

Islet exosomes from 26a DKO mice had markedly reduced levels of miR-26a (Fig 8K). MPHs after treatment with islet exosomes from 26a DKO mice exhibited decreased insulin-stimulated AKT activation (Fig 8L). As we shown previously, several regulators of liver metabolism and insulin signaling are direct targets of miR-26a [22]. Here, we further performed Ago2 immunoprecipitation sequencing analysis in the liver from 26a DKO mice and their WT littermates to systemically explore miR-26a targets. Based on the immunoprecipitation sequencing data (S14A Fig, S5 Table), together with target prediction (S14B Fig), luciferase reporter assay (S14C Fig), and QRT-PCR analysis (S14D Fig), we found that inhibin subunit beta A (Inhba) and DNA methyltransferase 3 alpha (Dnmt3a) act as direct targets of miR-26a. In line with decreased exosomal miR-26a, MPHs treated with islet exosomes from 26a DKO mice had reduced levels of miR-26a and increased levels of miR-26a targets (Fig 8M). Moreover, we determined the effect of miR-26a deficiency on hepatic fatty acid metabolites using GC-MS analysis. It found that 13 lipids were elevated in the primary hepatocytes from 26a DKO mice compared with that of WT mice (Fig 8N, S6 Table), supporting a role of miR-26a on fatty acid metabolism.

Together, these results suggested that miR-26a deficiency in vivo contributed to the reduced function of β cells and β-cell–derived exosomes, which promoted obesity-induced glucose intolerance and insulin resistance.

## Discussion

In this study, we show that miR-26a in β cells regulates insulin levels and insulin action in two distinct ways. In a conventional way, miR-26a prevents obesity-induced hyperinsulinemia in endogenous β cells through two mechanisms: miR-26a reduces compensatory β cell hyperplasia by decreasing β cell replication stimulated by excess nutrition and inhibits excess insulin secretion in response to hyperglycemia by modulating actin remodeling. In an unconventional way, by acting as a mediator of organ–organ crosstalk, exosomal miR-26a secreted from β cells improves insulin sensitivity and metabolic homeostasis in target cells of distal peripheral tissues. With the convergence of these local and distal regulatory modes, β cell miR-26a prevents obesity-induced hyperinsulinemia and insulin resistance. These data not only delineate for the first time a role for pancreatic miR-26a in the pathophysiology of diabetes but also exemplify a novel, to our knowledge, mechanism by which regulators within β cells modulate gene expression and metabolic homeostasis in distal tissues.

A number of miRNAs have been implicated in β cell mass and function, but only a few have been investigated in vivo [15]. In this study, we uncovered the in vivo function of β cell miR-26a using two new genetic mouse models. β-cell–specific overexpression of miR-26a in mice alleviated obesity-induced hyperinsulinemia and insulin resistance, while genetic deficiency of miR-26a in mice exerted the opposite effect. We demonstrate that β cell miR-26a markedly prevents HFD-induced hyperinsulinemia resulting from a decrease in β cell hyperplasia and insulin secretion. Mechanistically, miR-26a directly targets several important regulators involved in β cell replication and insulin secretion, including *Cacna1c*, *Ctgf*, *Crebrf*, *Esr1*, *Ext1*, *Mtpn*, *Onecut2*, *Pja2*, *Plcb1*, *Pfkfb2*, *Rhoq*, and *Sox5*. CTGF is necessary for β cell replication by inducing positive cell cycle regulators, including hepatocyte growth factor (HGF) and integrin subunit beta 1 (ITGB1) [41], and CTGF haploinsufficiency in mice prevents pregnancy-induced β cell hyperplasia [42]. The CREBRF variant is associated with increased BMI and increases obesity risk [43]. Meanwhile, CREBRF can function as an oncogene and promote gastric cancer cell proliferation [44]. Of note, miR-26a can induce cell cycle arrest and inhibit cancer cell proliferation by targeting multiple oncogenes such as enhancer of zeste 2 polycomb repressive complex 2 subunit (EZH2), Lin-28 homolog B (Lin28B), CCND2 and cyclin E2 (CCNE2) [19,45,46], which might also contribute to the inhibition of miR-26a on β cell hyperplasia. MTPN, an important component of the machinery controlling late stages of insulin secretion, is required for GSIS [47]. ONECUT2 promotes insulin secretion by suppressing granuphilin, which is an Rab GTPase effector that functions as an inhibitor of insulin exocytosis [48]. CACNA1C, PLCB1, and PJA2 are positive regulators of GSIS [49–51]. ESR1 has been shown to preserve insulin secretion and promote β cell survival [52]. EXT1 positively regulates insulin secretion and β cell proliferation [53]. These newly identified miR-26a targets, together with certain previously known targets, may act coordinately to maintain β cell mass and function. Of note, we observed that miR-26a can modulate actin remodeling and focal adhesion, as evidenced by its effect on FAK and its downstream signaling effectors. In tumor progression, miR-26a has been implicated in the regulation of focal adhesion and actin cytoskeleton [54,55]. Certain miR-26a targets such as PAK2 [55], GSK3β [56], and ITGA5 [57] are regulators of focal adhesion. These observations provide a reasonable explanation for miR-26a’s function in FAK signaling pathway during insulin secretion, although the exact underlying mechanism(s) await further investigation. Interestingly, elegant studies suggest that insulin may act via insulin receptors to regulate the compensatory expansion of β cells under obese conditions. Therefore, the dual effects of miR-26a on β cell mass and insulin secretion may interact and ensure appropriate levels of insulin to maintain systemic metabolic homeostasis, providing the first evidence that pancreatic miR-26a plays an important role in the pathogenesis of T2D. In addition, recent studies suggest a role of miR-26a in T cells and vasculature [58–60]. It will be interesting for future studies to investigate the potential involvement of these miR-26a–mediated processes in β cell mass and function under diabetic conditions.

Pancreatic regulators have traditionally been known to modulate glucose homeostasis by controlling insulin levels, not by affecting insulin sensitivity [7]. Here, we provide several lines of evidence that exosomal miR-26a derived from β cells can enhance insulin sensitivity. First, miRNA profiling on serum exosomes revealed that miR-26a was significantly down-regulated in overweight humans. This reduction was recapitulated in serum exosomes from obese/diabetic mice. Notably, the exosomal miR-26a level was negatively associated with BMI, HOMA-IR, fasting blood glucose, and other obesity/T2D features, suggesting a strong association of exosomal miR-26a with insulin sensitivity. Second, β cells can secrete miR-26a–containing exosomes that are then efficiently transported into recipient cells such as hepatocytes. Third, exosomes derived from β cells can modulate insulin sensitivity in vitro, which is positively correlated with the levels of exosomal miR-26a. Fourth, miR-26a in β cells can improve insulin sensitivity and metabolic homeostasis in vivo. RIP TG mice had improved insulin sensitivity and physiological functions in the liver and adipose tissue, along with increased miR-26a levels in these recipient tissues. Together, these findings suggest that β-cell–derived exosomal miRNAs are previously unappreciated circulating molecules that can function as modulators of gene expression and metabolic homeostasis in distant tissues, revealing a new mechanism of β cell regulators in organ–organ crosstalk. In this study, we identified Inhba and Dnmt3a as direct targets of miR-26a in the liver. Inhba can act as a promoter of various chronic liver diseases such as liver fibrosis and cirrhosis [61,62]. Dnmt3a has recently been shown to be an epigenetic regulator of insulin resistance [63]. However, these two targets appear insufficient to account for the potent beneficial phenotypes of RIP TG mice. Of note, we and others have demonstrated that miR-26a can directly target several regulators critical for liver and adipose metabolism [22,64,65], which play important roles in obesity and diabetes. It is likely that these known miR-26a targets, together with certain undescribed targets, cooperate to mediate the functions of exosomal miR-26a in peripheral tissues.

It is widely accepted that insulin resistance is a central component in the etiology of T2D. Hyperinsulinemia, an excess of blood insulin levels in relation to circulating glucose levels, is traditionally viewed as compensation for systemic insulin resistance. However, numerous experimental and clinical studies provide evidence that sustained obesity-induced hyperinsulinemia can further exacerbate insulin resistance, leading to a vicious cycle between hyperinsulinemia and insulin resistance, thereby contributing to the onset of T2D [13,14]. It has been proposed that mild suppression of hyperinsulinemia is important for treating insulin resistance and obesity [14]. In this study, we demonstrate that miR-26a simultaneously prevents hyperinsulinemia and insulin resistance through conventional and unconventional regulatory modes, respectively. These dual impacts of β cell miR-26a strongly suggest it as an ideal therapeutic target for T2D. Moreover, our previous studies identified protective roles of hepatic miR-26a in liver metabolism, insulin sensitivity, and liver injury [22,66,67]. In addition, miR-26a generally acts as a tumor suppressor [19,45] and has been proposed as a potential therapeutic target for cancer treatment [68]. Therefore, our findings strengthen the significance of miR-26a in human diseases and highlight miR-26a as an attractive target for the treatment of metabolic diseases, such as T2D.

In summary, this study reveals the significance of pancreatic miR-26a in insulin sensitivity. Reducing miR-26a in β cells contributes to obesity-induced metabolic abnormalities. Conversely, restoring miR-26a in β cells prevents obesity-induced metabolic damage. On one hand, it inhibits hyperinsulinemia endogenously by decreasing β cell hyperplasia and insulin secretion. On the other hand, it improves insulin sensitivity and metabolic homeostasis exogenously in peripheral tissue through circulating exosomes. These findings reveal for the first time, to our knowledge, a novel regulatory mechanism by which pancreatic regulators act at distal sites and may pave the way for developing treatments for obesity-associated metabolic syndrome.

## Materials and methods

### Ethics statement

All human studies were conducted according to the principles of the Declaration of Helsinki and approved by Ethics Committee of West China Hospital, Sichuan University (no. 2016590). Written informed consent was obtained from all subjects. All animal studies were approved by the Medical Ethics Committee of Sichuan University (protocol 2016155A).

### Clinical specimens

Samples of fasting venous blood were obtained from 24 individuals, and the entire cohort was subdivided into subgroups of healthy lean (BMI ≤ 25 kg/m^2^; *n* = 7) and overweight individuals (BMI > 25 kg/m^2^; *n* = 17). Human sera were centrifuged at 3,000 rpm for 15 minutes at 4°C to remove whole cells, cell debris, and aggregates. The metabolic parameters of bloods, including fasting glucose, fasting insulin, HbA1c, triglycerides, total cholesterol, HDL, and LDL were tested by the Department of Laboratory Medicine of West China Hospital. The study was approved by the Ethics Committee of Sichuan University. All subjects gave written informed consent before blood collection.

### Animals

Generation of TG (CAG-Neo-STOP^fl^-Mir26a1; The Jackson Laboratory, Bar Harbor, ME, USA) mice has been described previously [20]. Pancreatic β-cell–specific miR-26a TG mice (RIP TG) were generated by crossing TG (CAG-Neo-STOP^fl^-Mir26a1) mice with RIP-Cre mice (expressing Cre under the rat insulin-2 promoter; The Jackson Laboratory). Adipose-specific miR-26a TG mice (AP2 TG) were generated by crossing TG with Fabp4-Cre mice. Genotyping of RIP TG mice was performed by PCR using the primers listed in S7 Table. RIP TG and littermate TG (CAG-Neo-STOP^fl^-Mir26a1) mice were used for experiments.

The miR-26a-1–deficient mice and miR-26a-2–deficient mice were generated by using CRISPR/Cas9 technology. The genotyping of the miR-26a-1–knockout mice and miR-26a-2–knockout mice were verified by sequencing of the PCR fragments on genomic DNA isolated from tail tips. Then, miR-26a-1– and miR-26a-2–knockout mice were intercrossed to generate 26a DKO mice. Genotyping of 26a DKO mice was performed by PCR using primers listed in S7 Table.

All animal models were on a C57BL/6 background unless otherwise stated. For *db*/*db* mice, the respective WT control mice were on a BLKS background. ob/ob, db/db, and their controls were purchased from Shanghai Research Center for Model Organisms or Nanjing Biomedical Research Institute of Nanjing University, China. Mice were housed in a specific-pathogen–free (SPF) animal facility at the Institute of State Key Laboratory of Biotherapy and Cancer Center at the West China Hospital, Sichuan University. Mice were fed a standard laboratory CD or an HFD (60% Kcal fat; Research Diets, D12492; New Brunswick, NJ, USA), water ad libitum under a 12-hour light–dark cycle (lights on from 8 AM to 8 PM) at constant temperature (22°C). Male mice were used for all indicated studies.

### Cell culture and primary hepatocytes

The β cell lines Min6 and INS-1 were kindly provided by Professor Chunbo Teng (Northeast Forestry University, Harbin, China). Min6 cells were maintained in growth medium (RPMI 1640 with 2 mM glutamine, 50 μM β-mercaptoethanol [Sigma-Aldrich, St. Louis, MO, USA], 2% B27 [Gibco, Gaithersburg, MD, USA], 10% FBS, 1% penicillin/streptomycin [P/S]). INS-1 cells were cultured in growth medium (RPMI 1640 with 2 mM glutamine, 1 mM sodium pyruvate, 10 mM HEPES, 50 μM β-mercaptoethanol, 10% FBS, 1% P/S). The human embryonic kidney cell line 293T (ATCC, CRL3216; Manassas, VA, USA) were cultivated in growth medium (DMEM with 4.5 g/L glucose, 10% FBS, 1% P/S).

MPHs were isolated by the two-step collagenase perfusion method described previously [22]. Briefly, the mouse was anesthetized and infused with 50 ml solution I (calcium and magnesium-free EBSS containing 0.5 mM EGTA) via the vena cava, followed by 60 ml collagenase-containing solution II (1× HBSS [pH 7.2] with 0.3 mg/ml collagenase II and 40 μg/ml trypsin inhibitor). Cells from digested livers were teased out, suspended in DMEM medium, filtered through 70 μm filter, and centrifuged at 600 rpm for 2 minutes. The pellet was resuspended with DMEM, mixed with Percoll, and centrifuged at 600 rpm for 10 minutes. The cell pellet was resuspended with complete DMEM medium with 10% FBS after two washes and seeded into cell culture plates. For conditional culture, MPHs were washed with 1× PBS and cultured in DMEM medium supplemented with 10% mouse serum isolated from CD- or HFD-fed mice for 24 hours after being cultured overnight for adhesion, followed by insulin (10 nM) treatment for 0, 5, and 15 minutes to test insulin signaling. All cells were cultured in a humidified incubator at 37°C, 5% CO_2_.

### Primary brown preadipocytes isolation and differentiation

Primary brown preadipocytes were isolated from 4-week–old WT mice as described previously [69]. Adipocyte differentiation was induced by DMEM medium containing 10% FBS, isobutylmethylxanthine (0.5 mM), indomethacin (125 nM), dexamethasone (0.5 μM), insulin (850 nM), T3 (1 nM), and rosiglitazone (0.5 μM). Two days after induction, cells were switched to maintenance DMEM medium containing 10% FBS, insulin (850 nM), T3 (1 nM), and rosiglitazone (0.5 μM). The adipocytes were fully differentiated after 6–7 days of induction.

### Islet isolation and culture

Mouse islets were isolated by collagenase XI (Sigma-Aldrich) perfusion and digestion as described previously with minor modifications [70]. Individual islets were hand-picked and cultured in RPMI 1640 medium with 10% FBS before further experiments.

### Exosome isolation and characterization

All in vitro experiments were carried out using exosome-depleted FBS (System Biosciences, Palo Alto, CA, USA). Mouse and human sera or cell culture were centrifuged at 12,000 × *g* for 10 minutes to remove cell debris and aggregates. Exosomes from the supernatant were isolated by utilizing the commercial kit (System Biosciences) according to manufacturer’s instructions. Western blot analysis was performed using antibodies for exosomal markers (anti-TSG101 and anti-CD63) to confirm the characterization of exosomes. The size of purified exosomes was determined using a NanoSight analysis (Malvern Instruments, Malvern, UK). Transmission electron microscopy was used to further verify purified exosomes. Briefly, exosomes were loaded on formvar/carbon-coated copper grids for 10 minutes and then fixed with 2% phosphotungstic acid solution (pH 7.0) for 1 minute. Grids were air-dried and visualized using a Tecnai G2 Spirit (FEI, Hillsboro, OR, USA) transmission electron microscope at 80 kV.

### In vitro exosome treatment

MPHs were isolated and cultured overnight with 80% density in 12 well plates. Exosomes isolated from mouse sera or culture media of Min6 cells or purified islets were transferred to the recipient MPHs. 24–48 hours post-treatment, MPHs were collected or used for the following applications.

### Coculture assay

Size-matched islets from RIP TG or WT littermates were selected and cocultured with MPHs using a transchamber (0.4-μm polycarbonate filter; Millipore, Burlington, MA, USA), with MPHs placed in the lower chamber and islets in the upper chamber. Alternatively, Min6 cells transfected with miR-26a mimics or negative controls were cocultured with MPHs.

For insulin signaling testing, MPHs were serum-starved for 6 hours, treated with insulin (10 nM) for 0, 5, or 15 minutes, and then collected for western blotting. For gene expression measurement, cells in both chambers were collected for total RNA extraction and QRT-PCR analysis.

### Transfection

Min6 cells were transfected with miR-26a mimics or negative control (GenePharma, Shanghai, China) at 40 nM using Hiperfect Transreagent (Qiagen, Hilden, Germany), according to the manufacturer’s protocol. 24 hours post-transfection, Min6 cells were placed in medium containing exosome-depleted FBS for 48 hours, and then the media were collected for exosome isolation.

### Oxygen consumption assay

Primary brown preadipocytes were seeded (10,000 cells/well) and differentiated in XF cell culture microplates (Seahorse Bioscience, Billerica, MA, USA) as described above. Cells were transfected with miR-26a mimics or NCs for 48 h before performing the assay. Adipocytes were washed twice and maintained in XF assay medium (25 mM glucose, 2 mM glutamine, 1 mM sodium pyruvate) for 1 h at 37°C without CO_2_. OCR was measured by XF Extracellular Flux Analyzer (Seahorse Biosciences). During measurement, cells were treated with oligomycin (2.5 μM), carbonyl cyanide 4-(trifluoromethoxy) phenylhydrazone (FCCP, 2 μM), and antimycin A (0.5 μM) and rotenone (0.5 μM) in succession.

### RNA extraction and QRT-PCR

Total RNA was isolated using TRIzol reagent (Thermo Fisher Scientific, Waltham, MA, USA) according to the manufacturer’s protocol. The pancreas tissue was flash frozen in RNAlater (Qiagen) overnight before RNA isolation. Total RNA was subjected to reverse transcription into cDNA by using M-MLV Reverse Transcriptase (Invitrogen, Carlsbad, CA, USA). QRT-PCR was performed in 10-μl reaction volumes containing cDNA along with specific primers and SYBR Green PCR master mix (Qiagen). Primer sequences for QRT-PCR are described in S8 Table.

### Western blotting

Cells and tissues were homogenized in whole-cell lysates (RIPA buffer, Thermo Fisher Scientifc) with protease inhibitors and phosphatase inhibitors (Sigma-Aldrich, P8340, P5726, P0044), sonicated, and centrifugated to remove insoluble material. 30–40 μg of total proteins was separated by SDS/PAGE (10%), and electrically transferred onto PVDF membranes. Membranes were incubated with primary antibodies for overnight at 4°C after blocking in 5% milk in TBST for 1 hour and then incubated with secondary antibodies for 2 hours at room temperature. The immune complexes were detected using Chemiluminescent HRP Substrate (Millipore). Antibodies used in western blotting were listed in S9 Table.

### Metabolic measurements

Unless otherwise specified, random blood glucose was measured in the free-feeding condition, while fasting blood glucose was measured after starvation for 16 hours. Blood glucose was determined through tail vein bleeding with the use of a portable glucometer (Abbott Laboratories, Chicago, IL, USA). For insulin measurement, blood samples were collected from the tail vein and centrifuged at 3,000 rpm, 4°C for 10 minutes. Serum insulin levels were determined using the Ultra-Sensitive Mouse Insulin ELISA Kit (Crystal Chem, Elk Grove Village, IL, USA) according to the manufacturer’s protocol. HOMA-IR was calculated as the following formula: fasting glucose (mg dl^−1^) × fasting insulin (μU ml^−1^)/405. Plasma concentrations of triglycerides, total cholesterol, and HDL and LDL cholesterol were measured at the State Key Laboratory of Biotherapy and Cancer Center, Sichuan University. Hepatic concentrations of triglycerides were measured using a commercial kit (BioVision, Milpitas, CA, USA) according to the manufacturer’s instructions.

### GTTs and ITTs

GTT and ITT measurements were performed as previously described [71]. Blood glucose levels were measured using a glucometer. For GTTs, mice were fasted for 16 hours and then injected intraperitoneally with D-glucose (2 g/kg BW; Sigma-Aldrich) in sterile water. For ITTs, mice were fasted for 6 hours and then injected with human insulin (1 U/kg BW; Eli Lilly, Indianapolis, IN, USA) in saline solution.

### In vitro insulin release from islets

Mouse islets were isolated, hand-picked, and cultured overnight in RPMI 1640 medium. Islets were preincubated in Krebs buffer containing 2.8 mM glucose at 37°C for 30 minutes and then stimulated with 2.8 mM glucose in the presence or absence of insulin secretagogues (16.7 mM glucose, 20 mM arginine, or 30 mM KCl) for 1 hour. Insulin concentrations were determined, and values are normalized to total insulin content [72].

### Ago2 RNA immunoprecipitation

The livers and islets were isolated from 6-week–old WT and 26a DKO mice fed an HFD for 3 days and then used for Ago2 RIP as described previously [73], with minor modifications. Briefly, the liver tissues and islets were washed and homogenized in lysis buffer (50 mM Tris-HCl [pH 7.5], 100 mM NaCl, 0.1 mM CaCl_2_, 1% NP-40, 0.5% sodium deoxycholate, 0.1% SDS, and 1 mM DTT) supplemented with protease inhibitors and RNase inhibitors. The supernatant lysates were collected by centrifuging with 15,000 × *g* for 15 min at 4°C, and an aliquot of lysate (10% of total) of each sample was taken as input. Protein G/A magnetic beads were washed with antibody buffer (1× PBS, 0.02% Tween 20) and incubated with 5 μg anti-Ago2 or anti-IgG antibodies for 4 h at room temperature with gentle rotation to form protein G/A–antibody complex. The complexes were washed and then incubated with precleared lysates at 4°C overnight with gentle rotation. After binding, the beads were collected and washed with low salt buffer (50 mM Tris-HCl [pH 7.5], 150 mM NaCl, 1 mM MgCl_2,_ 0.5% NP-40, and 0.5 mM DTT) 3 times and high salt buffer (50 mM Tris-HCl [pH 7.5], 600 mM NaCl, 1 mM MgCl_2,_ 0.5% NP-40, and 0.5 mM DTT) another 3 times. The precipitated RNA was isolated by adding TRIzol reagent directly to the washed beads following the manufacturer’s instructions. The isolated RNA was subjected to immunoprecipitation sequencing by Novogene technology company limited (Beijing, China).

### Fatty acid metabolites analysis

Interscapular BATs were collected from WT and RIP TG mice fed an HFD for 16 weeks and frozen in liquid nitrogen immediately. 50- to 100-mg tissues were used for fatty acid determination by Novogene technology company limited using GC-MS (Agilent 6890N/5975B; Santa Clara, CA, USA).

For fatty acid metabolite determination in hepatocytes, primary hepatocytes were isolated from 6-week–old WT and 26a DKO mice fed an HFD for 3 days, as described above. 1 × 10^7^ hepatocytes were needed for each sample.

### Proteomics analysis

Proteomics analysis were performed as described previously [74]. Briefly, total proteins from the islets of WT and RIP TG mice fed an HFD for 2 days were extracted, followed by trichloroacetic acid precipitation and trypsin digestion. Samples were loaded on Q Exactive Plus system (Thermo Fisher Scientific), and data were processed with Proteome Discoverer 2.0 software and searched in the associated mouse protein database.

### HE staining and Oil-red O staining

Fresh tissues were fixed in 10% neutral-buffered formalin and then embedded in paraffin. Paraffin-embedded tissues were cut into 4-μm–thick sections and stained with HE. For Oil-red O staining, dissected livers were immediately frozen and subsequently embedded with OCT compound in optimal cutting temperature. Ten-μm–thick cryostat sections were prepared and stained for lipid visualization with Oil-red O. Staining images were collected using a light microscope (Olympus, Tokyo, Japan).

### IF and IHC

Paraffinized sections were deparaffinized and rehydrated, and antigen was retrieved with 10 mM Tris/EDTA (pH 9.0). The sections were permeabilized and blocked in PBS containing 1% BSA and 5% goat serum. Primary antibodies were incubated overnight at 4°C, followed by secondary antibodies incubation at 37°C for 1 hour. For immunofluorescence staining, anti-insulin antibody (Abcam, Cambridge, UK) and anti-glucagon antibody (Abcam) were used to detect β and α cells, respectively. Pancreatic sections were stained with anti-insulin antibody (HUABIO, Hangzhou, China) and anti-PCNA antibody (HUABIO), followed by tyramine amplification with fluorophores 488 and 594 and DAPI counterstaining to determine the proliferation of β cells. Insulin-positive (insulin+) cells showing nuclear colocalized staining for DAPI+ and PCNA+ were considered as proliferating β cells. Sections were visualized using a confocal microscope (LSM 880; Carl Zeiss, Oberkochen, Germany).

For immunohistochemistry staining, pancreatic sections were incubated with anti-insulin antibody (Abcam), followed by binding with horseradish peroxidase (HRP)-conjugated secondary antibodies, and detected with a DAB Kit and counterstained with hematoxylin. Quantification was performed using ImageJ.

### β cell mass

β cell mass was measured as described previously [75]. Pancreas were dissected, weighted, fixed, embedded in paraffin, and consecutively cut into 4-μm–thick sections. The insulin-stained area and total section area were determined in all islets in 9 sections from 3 parts of the pancreas. β cell mass was calculated by multiplying the area of insulin-positive cells/total pancreatic area with pancreatic weight (mg). Islet area and islet size were also calculated on insulin-stained sections. Quantification was performed by ImageJ.

### Imaging analysis of actin on isolated islets and β cells

Mouse islets were picked by hand selection under a dissecting microscope with pipette and stimulated afterwards with 2.8 mM or 16.7 mM glucose, where indicated. Min6 cells were grown on a glass bottom cell culture dish (NEST, Wuxi, China) and stimulated afterwards with 0 μM or 100 μM H_2_O_2_. Subsequently, islets and Min6 cells were fixed in 4% paraformaldehyde for 30 minutes at room temperature and subjected to immunostaining analyses. To characterize the cytoskeleton changes, fluorescent probes (Deoxyribonuclease I, Alexa Fluor 594 Conjugate, and Phalloidin-iFluor 488 Reagent) were used for simultaneous detection of monomeric G-actin and F-actin in islets and Min6 cells. Z-stack acquisition was performed on a Zeiss LSM 880 confocal microscope using a 40× objective. Images were processed using Zen software (Carl Zeiss).

### F-actin/G-actin ratio measurement

Isolation of F-actin to G-actin was performed as described previously [76]. Then samples from supernatant (G-actin) and pellet (F-actin) fractions were treated with TCA-acetone. After drying out overnight at 4°C, samples were dissolved in SDS-loading buffer, boiled for 10 minutes, and tested by western blot analysis. The F-actin/G-actin ratio was quantified by ImageJ.

### Luciferase assay

3′-UTR sequences of miR-26a targets were PCR-amplified with specific primers, followed by purification and restriction enzyme digestion. Sequences were cloned into the psiCHECK2.2 luciferase vector. This vector contains a renilla luciferase open reading frame and a constitutively expressed firefly luciferase gene, which is used to normalize transfection. The seed sequence of miR-26a was mutated using Q5 Site-Directed Mutagenesis Kit (New England Biolabs, Ipswich, MA, USA). 293T cells were cultured in 96-well plates and co-transfected with the indicated 3′-UTR reporter construct and miR-26a mimics or negative control (40 nM) using Attractene reagent (Qiagen). Cells were collected 24 hours after transfection and assayed using the Dual-Luciferase Reporter Assay System (Promega, Madison, WI, USA) according to the manufacturer’s protocol. Renilla luciferase activity was normalized to the corresponding firefly luciferase activity and plotted as a percentage of the control. Primer sequences for plasmid construction are described in S10 Table.

### Statistical analysis

All data represent at least 3 independent experiments and are shown as mean ± SD. If not mentioned otherwise in the figure legend, statistical significance (*P ≤ 0.05, **P ≤ 0.01, ***P ≤ 0.005; NS, *P* > 0.05) was determined by Student *t* test or two-tailed ANOVA.

## Supporting information

S1 Fig miRNA expression in exosomes and their correlation with clinical features. (A) Electron microscopy analysis of human serum exosomes. Scale bar, 50 μm. (B) NanoSight analysis of particle size of human serum exosomes. (C) Western blot analysis of exosome-specific markers TSG101 and CD63 on total proteins extracted from human serum exosomes. (D and E) Correlation between serum exosomal miR-26a levels and HbA1c (D) or triglycerides (E) in an overweight human cohort (*n* = 16–17). (F) Expression of miR-221-3p in serum exosomes of lean (*n* = 7) and obese (*n* = 17, BMI > 25) individuals. (G) Correlation between serum exosomal miR-221-3p levels and BMI, HOMA-IR, fasting glucose levels, or fasting insulin levels (*n* = 23–24). The data underlying this figure may be found in S2 Data and S1 Raw Images. Data are shown as mean ± SD. Student *t* test. BMI, body mass index; CD63, CD63 molecule; HOMA-IR, homeostatic model assessment index of insulin resistance; miRNA, microRNA; TSG101, tumor susceptibility gene 101 protein
(TIF)

S2 Fig MiR-26a expression remains unchanged in the brain of diabetic mice. (A–C) Expressions of miR-26a in the brain of db/db mice (*n* = 5–6) (A), ob/ob mice (*n* = 4–8) (B), and WT DIO mice (*n* = 6) (C). The data underlying this figure may be found in S2 Data. Data are shown as mean ± SD. Student *t* test. db/db mice, leptin-receptor–deficient mice; DIO, diet-induced obese; ob/ob, leptin-deficient mice
(TIF)

S3 Fig Effects of β-cell–specific overexpression of miR-26a on mice fed a CD. (A) Expression of miR-26a in muscle and liver tissues of RIP TG mice and WT littermate controls (*n* = 4). (B–H) The effects of miR-26a on mice fed a CD. (B) Total BW (*n* = 7–8). (C) GTT (*n* = 7). (D) ITT (*n* = 7). (E) Blood glucose levels of mice that were fed with a CD for 8 or 15 weeks. Random or fasting conditions are noted (*n* = 8–12). (F) Blood insulin levels during GTT (*n* = 7). (G) Representative IHC staining of insulin in pancreatic islets (scale bar, 50 μm) (*n* = 3). (H) Representative HE-stained liver and eWAT (scale bar, 50 μm) (*n* = 3). The data underlying this figure may be found in S2 Data. Data are shown as mean ± SD. 2-tailed ANOVA (B–D) and Student *t* test (A, E, and F). BW, body weight; CD, chow diet; eWAT, epididymal white adipose tissue; GTT, glucose tolerance test; HE, hematoxylin–eosin; IHC, immunohistochemistry; ITT, insulin tolerance test; RIP, rat insulin promoter; TG, transgenic; WT, wild type
(TIF)

S4 Fig MiR-26a remains unchanged in the brain and hypothalamus of RIP TG mice fed an HFD. (A and B) Expressions of miR-26a in the brain (A) and hypothalamus (B) of RIP TG mice and WT littermates fed an HFD for 16 weeks (*n* = 4–5). The data underlying this figure may be found in S2 Data. Data are shown as mean ± SD. Student *t* test. HFD, high-fat diet; RIP, rat insulin promoter; TG, transgenic; WT, wild type.
(TIF)

S5 Fig Exosomal miR-26a regulates insulin sensitivity. (A and B) Min6 (A) or INS-1 (B) cells were transfected with miR-26a mimics (miR-26a) or NCs. Culture medium was collected and purified by 0.4-μm filters, which allows for small molecules and vesicles such as exosomes to pass through. The expression of miR-26a was determined by QRT-PCR. (A) Levels of miR-26a in Min6 cells (left panel) or filtered culture medium (right panel) (*n* = 3). (B) Levels of miR-26a in INS-1 cells (left panel) or filtered culture medium (right panel) (*n* = 3). The data underlying this figure may be found in S2 Data. Data are shown as mean ± SD. **P < 0.01, ***P < 0.005, Student *t* test. INS-1 cells, rat β cells; Min6 cells, murine β cells; NC, negative control; QRT-PCR, quantitative reverse transcriptase PCR
(TIF)

S6 Fig High concentration of glucose reduces exosomal miR-26a secreted by Min6 cells. Exosomal miR-26a in Min6 cells treated with 2.8 mM or 16.7 mM glucose for 24 hours (*n* = 3). The data underlying this figure may be found in S2 Data. Data are shown as mean ± SD. * P < 0.05, Student *t* test. Glu, glucose; Min6 cells, murine β cells
(TIF)

S7 Fig Exosomal miR-26a in the serum of DIO adipo TG mice. Exosomal miR-26a in the serum of WT and AP2 TG mice fed an HFD (*n* = 6). The data underlying this figure may be found in S2 Data. Data are shown as mean ± SD. Student *t* test. AP2 TG, adipocyte-specific miR-26a overexpression mouse; AP2, adipocyte fatty acid binding protein; DIO, diet-induced obese; HFD, high-fat diet; TG, transgenic; WT, wild type
(TIF)

S8 Fig In vivo effects of β cell miR-26a overexpression on the functions of peripheral tissues. (A–E and H–J) 6- to 8-week–old RIP TG and WT littermate controls were fed an HFD for 16 weeks. (A and B) Expression of pri- and pre-miR-26a in the VAT (A) or BAT (B) (*n* = 4–6). (C–E) Plasma cholesterol (C), HDL (D), and LDL (E) levels in RIP TG and WT littermate controls fed a CD or an HFD (*n* = 5–6). (F) Heat map of mRNA levels of hepatic genes involved in liver metabolism and function. Red and blue depict higher and lower gene expression, respectively. Color intensity indicates magnitude of expression differences. Expression of all listed genes was comparable in two mouse groups (*n* = 4–6). (G) OCR for primary brown adipocytes isolated from WT and transfected with NCs or miR-26a mimics. Basal OCR, uncoupled OCR, and maximal OCR are presented (*n* = 6–9). (H) Representative HE-stained WAT. (I and J) Heat map of mRNA levels of genes involved in WAT function and metabolism. Differentially (I) or nondifferentially (J) expressed genes were shown separately (*n* = 4–6). The data underlying this figure may be found in S2 Data. Data are shown as mean ± SD. *P < 0.05, **P < 0.01, ***P < 0.005, Student *t* test. BAT, brown adipose tissue; CD, chow diet; HDL, high-density lipoprotein; HE, hematoxylin–eosin; HFD, high-fat diet; LDL, low-density lipoprotein; NC, negative control; OCR, oxygen consumption rate; pre-miR-26a, precursor miR-26a; pri-miR-26a, primary miR-26a RIP, rat insulin promoter; TG, transgenic; VAT, visceral adipose tissue; WAT, white adipose tissue; WT, wild type.
(TIF)

S9 Fig MiR-26a regulates insulin secretion. (A) Insulin contents extracted from islets (*n* = 3). (B) AKT phosphorylation in INS-1 cells that transfected with NCs or miR-26a mimics and treated with insulin (100 nM) for indicated times. (C) Insulin-stimulated AKT phosphorylation in islets isolated from WT and RIP TG mice fed an HFD for 15 weeks. Islets in each group were isolated from 4 mice and pooled together for western blot assay. (D and E) Proteomic analysis on islets of either RIP TG or WT littermate controls fed an HFD for 2 days. Gene ontology analysis of differentially expressed islet proteins between WT littermates and RIP TG mice. (D) Gene ontology (BP direct) analysis. (E) Gene ontology (CC direct) analysis. (F) Certain genes associated with focal adhesin in Fig 6E were verified by QRT-PCR. (G) Representative IF imaging of G-actin and F-actin in Min6 cells (scale bar, 50 μm) (*n* = 3). The data underlying this figure may be found in S2 Data and S1 Raw Images. Data are shown as mean ± SD. *P < 0.05, **P < 0.01, Student *t* test. BP, biological process; CC, cellular component; F-actin, filamentous actin; G-actin, globular actin; HFD, high-fat diet; IF, immunofluorescence; INS-1 cells, rat β cells; Min6 cells, murine β cells; NC, negative control; QRT-PCR, quantitative reverse transcriptase PCR; RIP, rat insulin promoter; TG, transgenic; WT, wild type.
(TIF)

S10 Fig MiR-26a inhibits β cell proliferation. Representative IF staining for insulin and PCNA in pancreas from RIP TG and WT controls fed an HFD for 16 weeks (scale bar, 20 μm) (*n* = 4). HFD, high-fat diet; IF, immunofluorescence; PCNA, proliferative cell nuclear antigen; RIP, rat insulin promoter; TG, transgenic; WT, wild type.
(TIF)

S11 Fig Generation of miR-26a knockout mice. (A) Scheme for generating 26a DKO mice. Knockout mouse lines for miR-26a-1 and miR-26a-2 were separately established by CRISPR technology, and then these two mouse lines were intercrossed to obtain 26a DKO mice. (B) Expression of miR-26a in islets of 26a DKO mice and WT controls (*n* = 3). The data underlying this figure may be found in S2 Data. Data are shown as mean ± SD. Student *t* test. WT, wild type; 26a DKO mice, miR-26a double knockout mice.
(TIF)

S12 Fig Identification of miR-26a targets in islets. (A–L) Predicted consequential pairing of target region and miR-26a is shown.
(TIF)

S13 Fig MiR-26a deficiency promotes β cell proliferation. Representative IF staining for insulin and PCNA in pancreas from 26a DKO and WT controls fed an HFD for 8 weeks (scale bar, 20 μm) (*n* = 4). HFD, high-fat diet; IF, immunofluorescence; PCNA, proliferative cell nuclear antigen; WT, wild type; 26a DKO, miR-26a double knockout
(TIF)

S14 Fig Identification of miR-26a targets in the livers. (A) The top 25 enriched hepatic genes in WT mouse identified by RNA immunoprecipitation sequencing are presented in a heat map (*n* = 3). Red and blue depict higher and lower gene enrichment, respectively. Color intensity indicates magnitude of enrichment differences. (B) Predicted consequential pairing of target region and miR-26a is shown. (C) Relative luciferase activity in 293T cells transfected with reporter constructs containing the 3′ UTR of target genes and co-transfected with either miR-26a mimics or NCs. (D) Expression of miR-26a target genes in livers of WT and 26a DKO mice fed an HFD (*n* = 5–8). The data underlying this figure may be found in S2 Data. Data are shown as mean ± SD. *P < 0.05, ***P < 0.005, Student *t* test. HFD, high-fat diet; NC, negative control; WT, wild type; 26a DKO mice, miR-26a double knockout mice.
(TIF)

S1 Table 12 miRNAs examined in this study. miRNA, microRNA.
(DOCX)

S2 Table Fatty acid analysis in BATs. Metabolomic profiling of fatty acids in BATs from RIP TG mice and WT littermates fed an HFD (*n* = 4–6). BAT, brown adipose tissue; ND, not detected; RIP, rat insulin promoter; TG, transgenic; WT, wild type.
(XLSX)

S3 Table Proteomics analysis on islets. The proteins with differential expressions (*P* < 0.05) in islets of RIP TG mice compared with their WT littermates are listed below. RIP, rat insulin promoter; TG, transgenic; WT, wild type.
(DOCX)

S4 Table Ago2 RNA immunoprecipitation sequencing in islets. The top 50 enriched genes in islets of WT mice identified by Ago2 RNA immunoprecipitation sequencing are shown. Ago2, argonaute RISC catalytic component 2; WT, wild type.
(XLSX)

S5 Table Ago2 RNA immunoprecipitation sequencing in the livers. The top 25 enriched genes in the livers of WT mice identified by Ago2 RNA immunoprecipitation sequencing are shown. Ago2, argonaute RISC catalytic component 2; WT, wild type.
(XLSX)

S6 Table Fatty acid analysis in hepatocytes. Metabolomic profiling of fatty acids in primary hepatocytes isolated from 26a DKO and WT mice fed an HFD for 3 days (*n* = 4). HFD, high-fat diet; ND, not detected; WT, wild type; 26a DKO mice, miR-26a double knockout mice.
(XLSX)

S7 Table Primers for genotyping. (DOCX)

S8 Table Primers for QRT-PCR. QRT-PCR, quantitative reverse transcriptase PCR.
(DOCX)

S9 Table Antibodies used in this study. (DOCX)

S10 Table Primers for plasmid construction. (DOCX)

S1 Data The underlying data in Figs 1–8. (XLSX)

S2 Data The underlying data in S1–S14 Figs. (XLSX)

S1 Raw Images All the original WB images. WB, western blotting.
(PDF)

## Abbreviations

Acc: acetyl-CoA carboxylase
Acsl3/4: acyl-CoA synthetase long chain family member 3/4
Adamts19: ADAM metallopeptidase with thrombospondin type 1 motif 19
Adam17: ADAM metallopeptidase domain 17
Ago2: argonaute RISC catalytic component 2
Akt: AKT serine/threonine kinase
Amph: amphiphysin
Ap5m1: adaptor related protein complex 5 subunit mu 1
Atad2b: ATPase family AAA domain containing 2B
BAT: brown adipose tissue
Baz2b: bromodomain adjacent to zinc finger domain 2B
BMI: body mass index
Brap: BRCA1 associated protein
BW: body weight
Cacna1c: calcium voltage-gated channel subunit alpha1 C
Carmil1: capping protein regulator and myosin 1 linker 1
CAV1: caveolin 1
Ccnd2: cyclin D2
CD: chow diet
CD63: CD63 molecule
Cep350: centrosomal protein 350
Chordc1: cysteine and histidine rich domain containing 1
Cidea: cell death inducing DFFA like effector A
Col11a1: collagen type XI alpha 1 chain
Col22a1: collagen type XXII alpha 1 chain
Cox: cytochrome c oxidase
Cpt: carnitine palmitoyltransferase
Crebrf: CREB3 regulatory factor
Ctgf: connective tissue growth factor
Dab2: DAB adaptor protein 2
Dapk1: death associated protein kinase 1
db/db mice: leptin-receptor–deficient mice
DIO: diet-induced obese
Dnmt3a: DNA methyltransferase 3 alpha
Emr1: EGF-Like module receptor 1
Epha7: EPH receptor A7
ERK: extracellular signal-regulated kinase
Esr1: estrogen receptor 1
EV: extracellular vesicle
Ext1: exostosin glycosyltransferase 1
EZH2: enhancer of zeste 2 polycomb repressive complex 2 subunit
E2f7: E2F transcription factor 7
Fabp4: fatty acid binding protein 4
FAK: focal adhesion kinase
Fasn: fatty acid synthase
FLNA: filamin alpha
Foxo1: forkhead box O transcription factor 1
F-actin: filamentous actin
GAPDH: glyceraldehyde-3-phosphate dehydrogenase
GC-MS: gas chromatography–mass spectrometry
GSK3β: glycogen synthase kinase 3 beta
GSIS: glucose-stimulated insulin secretion
GTT: glucose tolerance test
Gys2: glycogen synthase 2
G-actin: globular actin
HDL: high-density lipoprotein
HE: hematoxylin–eosin
HFD: high-fat diet
HGF: hepatocyte growth factor
Hnrnpul1: heterogeneous nuclear ribonucleoprotein U like 1
HOMA-IR: homeostatic model assessment index of insulin resistance
IF: immunofluorescence
Igf1r: insulin like growth factor 1 receptor
IHC: immunohistochemistry
Il6: interleukin 6
Inhba: inhibin subunit beta A
Insr: insulin receptor
INS-1 cells: rat β cells
IRS-2: insulin receptor substrate 2
ITGB1: integrin subunit beta 1
ITT: insulin tolerance test
KEGG: Kyoto Encyclopedia of Genes and Genomes
LDL: low-density lipoprotein
Ldlr: LDL receptor
Lin28B: Lin-28 homolog B
Lman1: lecti mannose binding 1
Lrat: lecithin retinol acyltransferase
Mcc: MCC regulator of WNT signaling pathway
Megf9: multiple EGF like domains 9
Memo1: mediator of cell motility 1
Min6 cells: murine β cells
MIP: maximum intensity projection
miRNA: microRNA
Mki67: marker of proliferation Ki-67
MPHs: murine primary hepatocytes
MS: mass spectrometry
Mtpn: myotrophin
Nek10: NIMA related kinase 10
Neto1: neuropilin and tolloid like 1
Nras: neuroblastoma RAS viral oncogene
Nwd2: NACHT and WD repeat domain containing 2
ob/ob mice: leptin-deficient mice
OCR: oxygen consumption rate
Onecut2: one cut homeobox 2
pAkt: phosphorylated AKT
Pcdh9: protocadherin 9
PCK1: phosphoenolpyruvate carboxykinase 1
PCNA: proliferative cell nuclear antigen
Pdx1: pancreatic and duodenal homeobox 1
Pfkfb2: 6-phosphofructo-2-kinase/fructose-2,6-biphosphatase 2
Pgc1a: peroxisome proliferator-activated receptor gamma coactivator 1-alpha
Phf21a: PHD finger protein 21A
Pja2: praja ring finger ubiquitin ligase 2
Pkcθ: protein kinase C theta
Plcb1: phospholipase C beta 1
Pnpla2: patatin-like phospholipase domain-containing protein 2
Ppara: peroxisome proliferator activated receptor alpha
Pparg: peroxisome proliferator activated receptor gamma
Prdm16: PR domain containing 16
pre-miRNA: precursor miRNA
pri-miRNA: primary miRNA
Prkag2: protein kinase AMP-activated noncatalytic subunit gamma 2
Prkg1: protein kinase CGMP-dependent 1
Pxylp1: 2-phosphoxylose phosphatase 1
Pygl: glycogen phosphorylase L
QRT-PCR: quantitative reverse transcriptase PCR
Rb1: RB transcriptional corepressor 1
Rcn1: reticulocalbin 1
Ret: Ret proto-oncogene
Rhoq: Ras homolog family member Q
RIP: rat insulin promoter
Ryk: receptor like tyrosine kinase
Scd1: stearoyl-CoA desaturase
Scml4: scm polycomb group protein like 4
Slc26a4: solute carrier family 26 member 4
Sox5: SRY-box transcription factor 5
SPF: specific-pathogen–free
Spock2: SPARC/Osteonectin, CWCV and Kazal like domains proteoglycan 2
Tab2: TGF-beta activated kinase 1 binding protein 2
Tacc2: transforming acidic coiled-coil containing protein 2;TCF7L2, transcription factor 7 like 2
Tet2: tet methylcytosine dioxygenase 2
TG: transgenic
Tnfα: tumor necrosis factor α
Tnks2: tankyrase 2
Trps1: transcriptional repressor GATA binding 1
Tsc22d2: TSC22 domain family member 2
TSG101: tumor susceptibility gene 101 protein
T2D: type 2 diabetes
Ube4b: ubiquitination factor E4B
UCP1: uncoupling protein 1
UPLC-ESI-MS/MS: ultraperformance liquid chromatography–electrospray tandem mass spectrometry/mass spectrometry
Usp9x: ubiquitin specific peptidase 9 X-linked
VAT: visceral adipose tissue
WAT: white adipose tissue
WT: wild type
26a DKO mice: miR-26a double knockout mice
