# Supplementary figures and images for: Pancreatic β cell microRNA-26a alleviates type 2 diabetes by improving peripheral insulin sensitivity and preserving β cell function
Source: PLoS Biol. 2020 Feb 24;18(2):e3000603. doi: 10.1371/journal.pbio.3000603 (PMC7058362; doi:10.1371/journal.pbio.3000603)

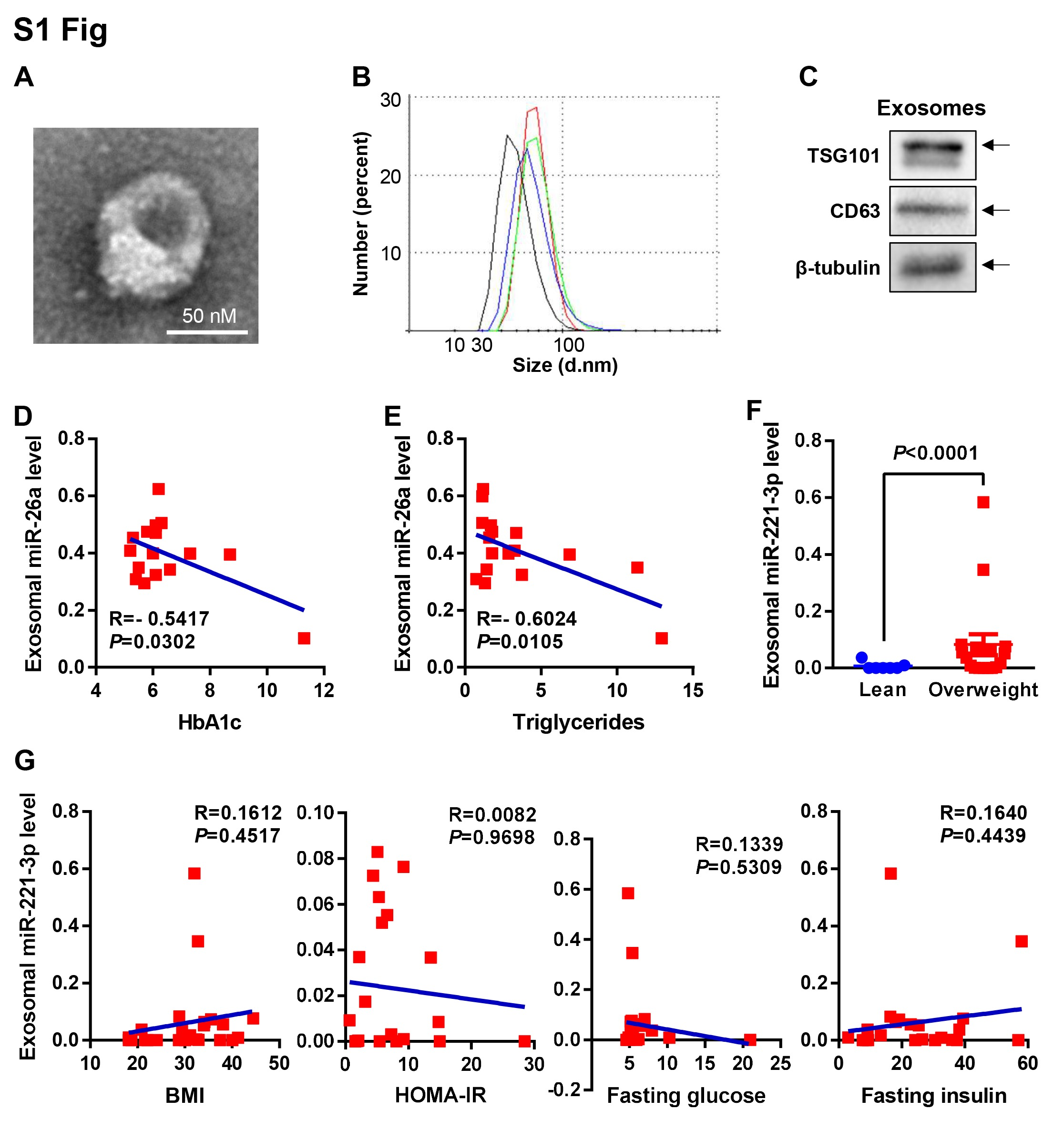

Supplement: S1 Fig — (A) Electron microscopy analysis of human serum exosomes. Scale bar, 50 μm. (B) NanoSight analysis of particle size of human serum exosomes. (C) Western blot analysis of exosome-specific markers TSG101 and CD63 on total proteins extracted from human serum exosomes. (D and E) Correlation between serum exosomal miR-26a levels and HbA1c (D) or triglycerides (E) in an overweight human cohort (n = 16–17). (F) Expression of miR-221-3p in serum exosomes of lean (n = 7) and obese (n = 17, BMI > 25) individuals. (G) Correlation between serum exosomal miR-221-3p levels and BMI, HOMA-IR, fasting glucose levels, or fasting insulin levels (n = 23–24). The data underlying this figure may be found in S2 Data and S1 Raw Images. Data are shown as mean ± SD. Student t test. BMI, body mass index; CD63, CD63 molecule; HOMA-IR, homeostatic model assessment index of insulin resistance; miRNA, microRNA; TSG101, tumor susceptibility gene 101 protein (TIF) [file pbio.3000603.s001.tif]

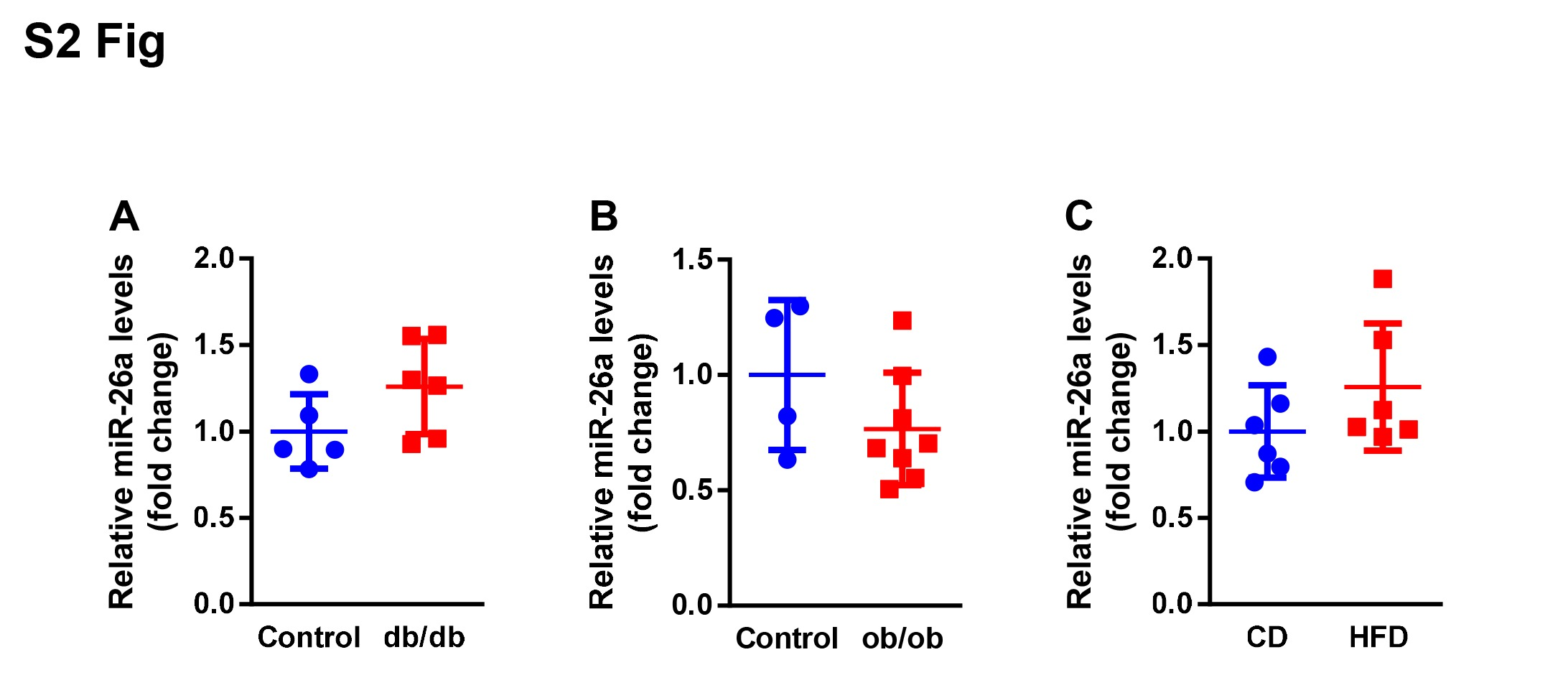

Supplement: S2 Fig — (A–C) Expressions of miR-26a in the brain of db/db mice (n = 5–6) (A), ob/ob mice (n = 4–8) (B), and WT DIO mice (n = 6) (C). The data underlying this figure may be found in S2 Data. Data are shown as mean ± SD. Student t test. db/db mice, leptin-receptor–deficient mice; DIO, diet-induced obese; ob/ob, leptin-deficient mice (TIF) [file pbio.3000603.s002.tif]

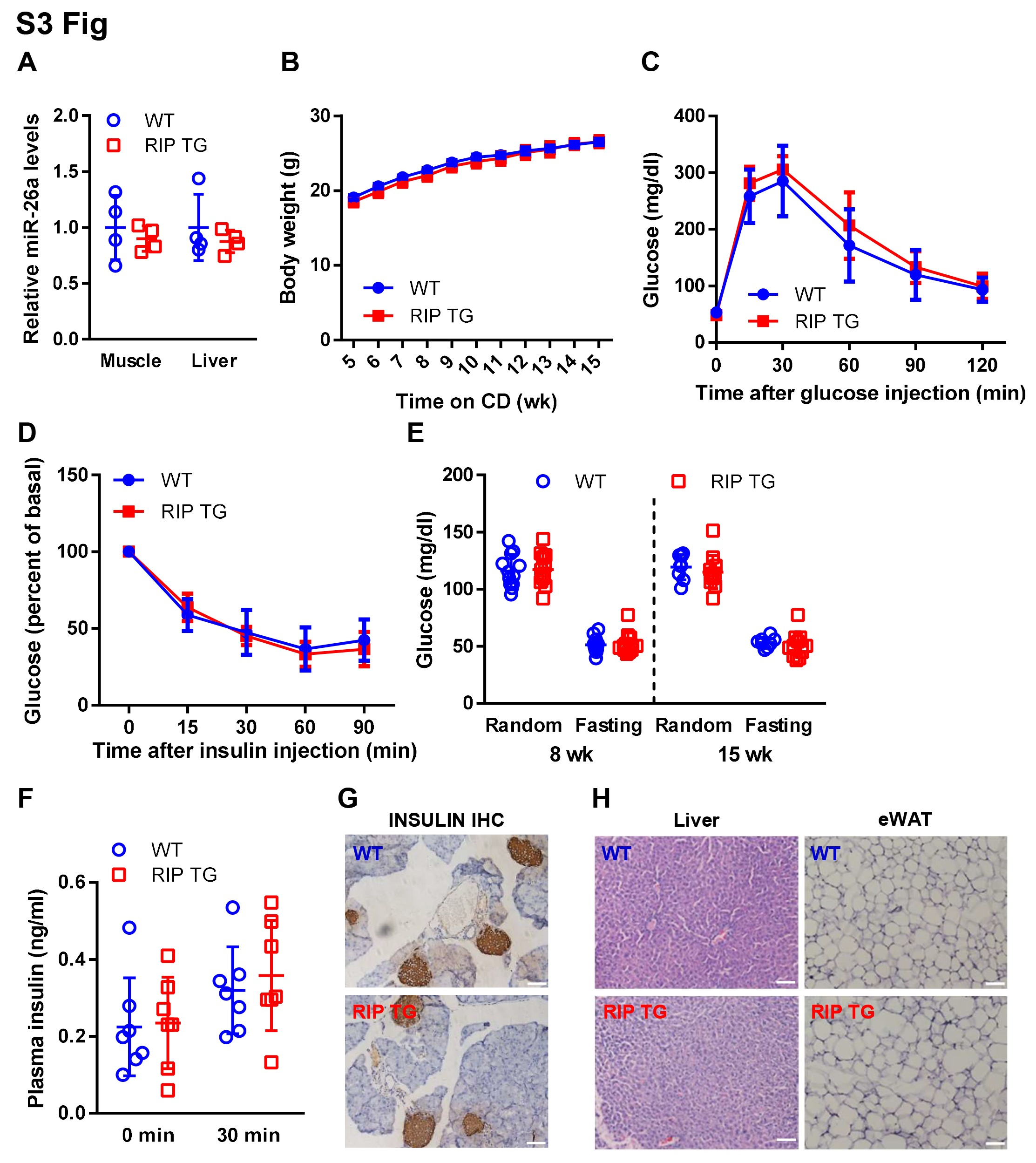

Supplement: S3 Fig — (A) Expression of miR-26a in muscle and liver tissues of RIP TG mice and WT littermate controls (n = 4). (B–H) The effects of miR-26a on mice fed a CD. (B) Total BW (n = 7–8). (C) GTT (n = 7). (D) ITT (n = 7). (E) Blood glucose levels of mice that were fed with a CD for 8 or 15 weeks. Random or fasting conditions are noted (n = 8–12). (F) Blood insulin levels during GTT (n = 7). (G) Representative IHC staining of insulin in pancreatic islets (scale bar, 50 μm) (n = 3). (H) Representative HE-stained liver and eWAT (scale bar, 50 μm) (n = 3). The data underlying this figure may be found in S2 Data. Data are shown as mean ± SD. 2-tailed ANOVA (B–D) and Student t test (A, E, and F). BW, body weight; CD, chow diet; eWAT, epididymal white adipose tissue; GTT, glucose tolerance test; HE, hematoxylin–eosin; IHC, immunohistochemistry; ITT, insulin tolerance test; RIP, rat insulin promoter; TG, transgenic; WT, wild type (TIF) [file pbio.3000603.s003.tif]

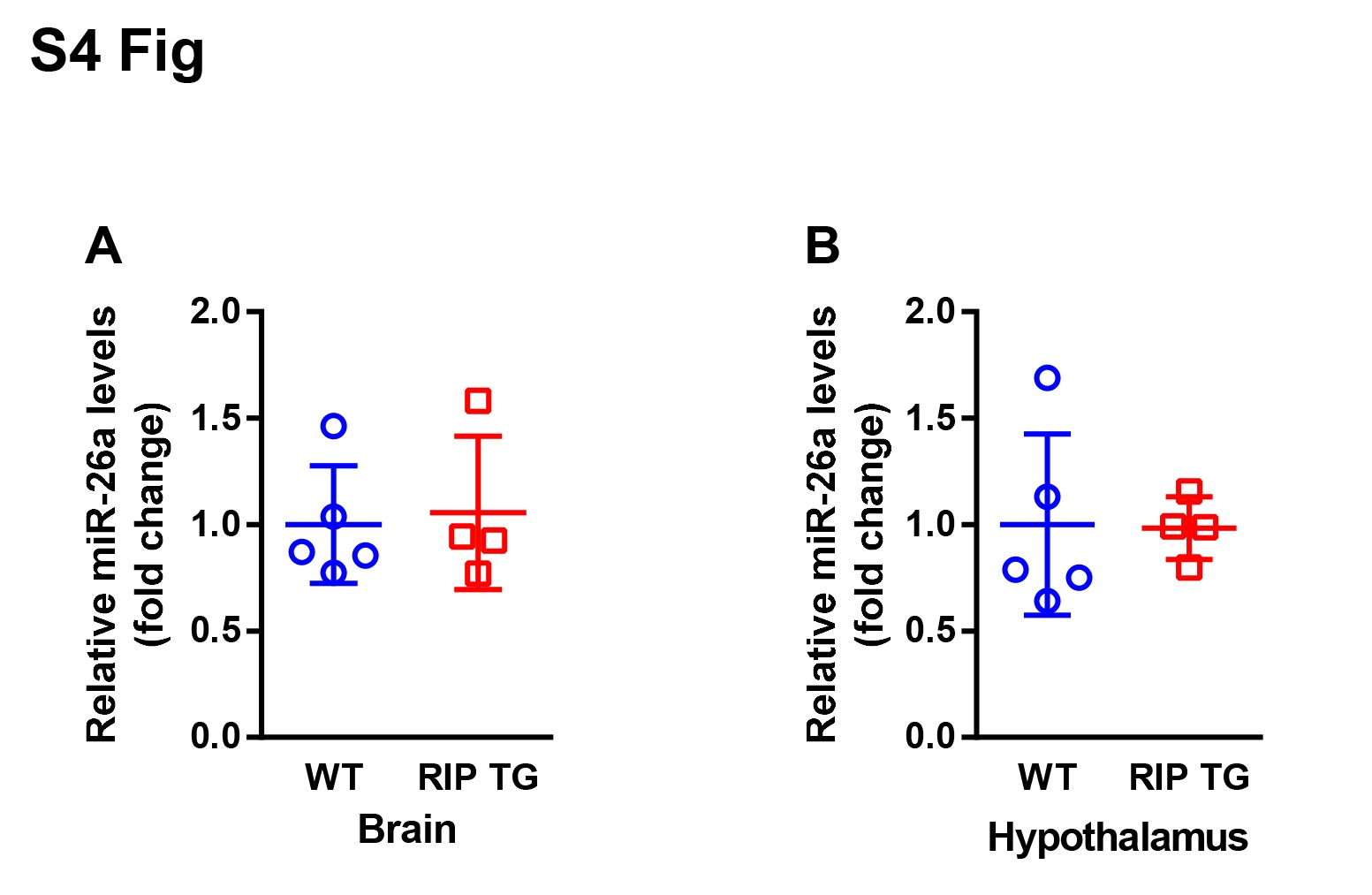

Supplement: S4 Fig — (A and B) Expressions of miR-26a in the brain (A) and hypothalamus (B) of RIP TG mice and WT littermates fed an HFD for 16 weeks (n = 4–5). The data underlying this figure may be found in S2 Data. Data are shown as mean ± SD. Student t test. HFD, high-fat diet; RIP, rat insulin promoter; TG, transgenic; WT, wild type. (TIF) [file pbio.3000603.s004.tif]

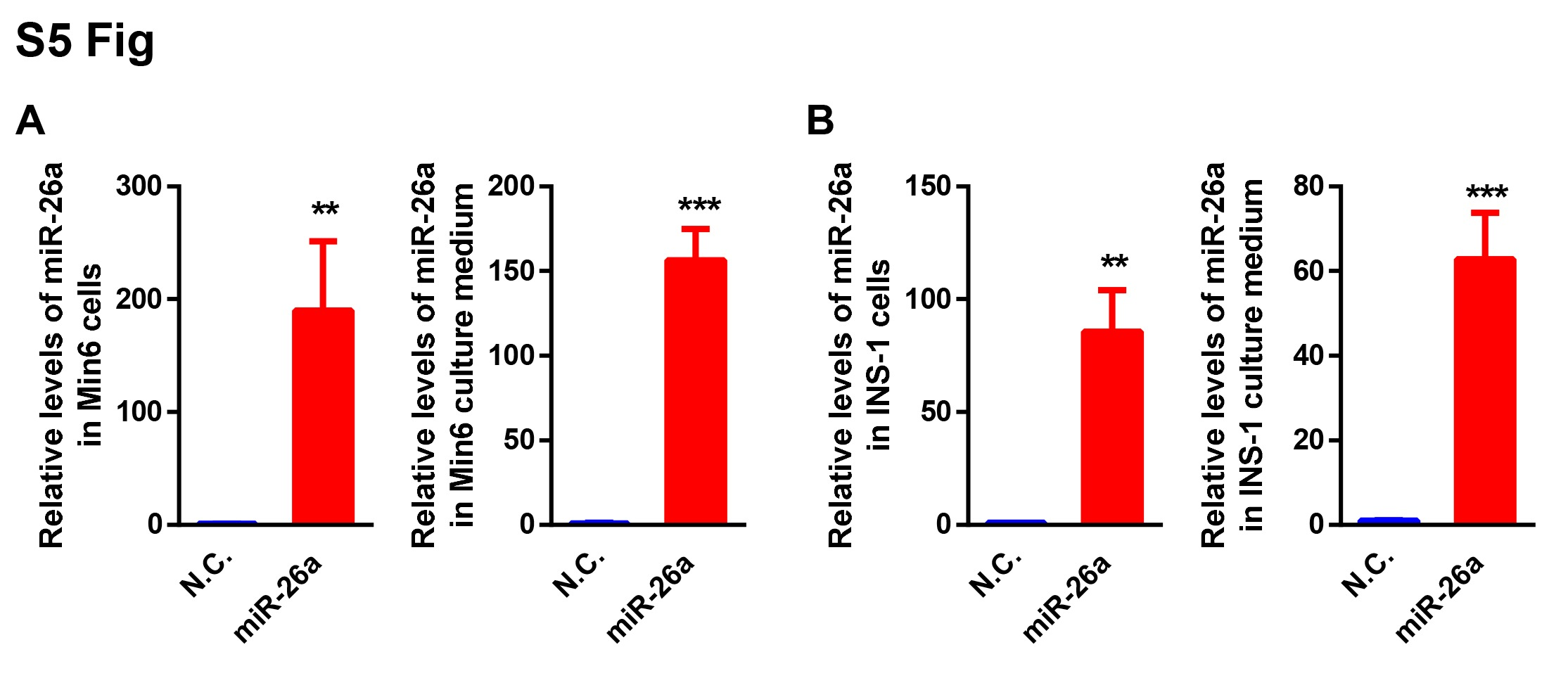

Supplement: S5 Fig — (A and B) Min6 (A) or INS-1 (B) cells were transfected with miR-26a mimics (miR-26a) or NCs. Culture medium was collected and purified by 0.4-μm filters, which allows for small molecules and vesicles such as exosomes to pass through. The expression of miR-26a was determined by QRT-PCR. (A) Levels of miR-26a in Min6 cells (left panel) or filtered culture medium (right panel) (n = 3). (B) Levels of miR-26a in INS-1 cells (left panel) or filtered culture medium (right panel) (n = 3). The data underlying this figure may be found in S2 Data. Data are shown as mean ± SD. **P < 0.01, ***P < 0.005, Student t test. INS-1 cells, rat β cells; Min6 cells, murine β cells; NC, negative control; QRT-PCR, quantitative reverse transcriptase PCR (TIF) [file pbio.3000603.s005.tif]

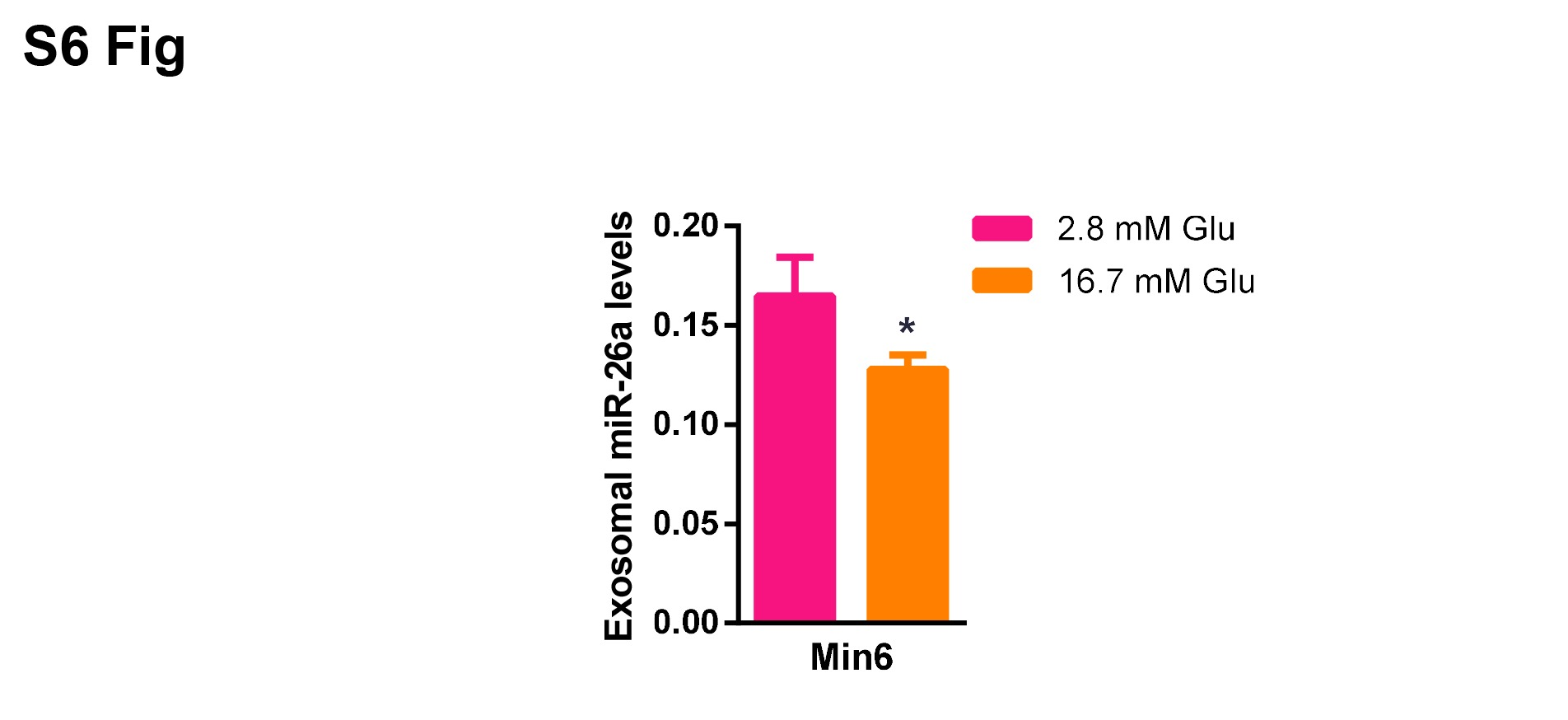

Supplement: S6 Fig — Exosomal miR-26a in Min6 cells treated with 2.8 mM or 16.7 mM glucose for 24 hours (n = 3). The data underlying this figure may be found in S2 Data. Data are shown as mean ± SD. * P < 0.05, Student t test. Glu, glucose; Min6 cells, murine β cells (TIF) [file pbio.3000603.s006.tif]

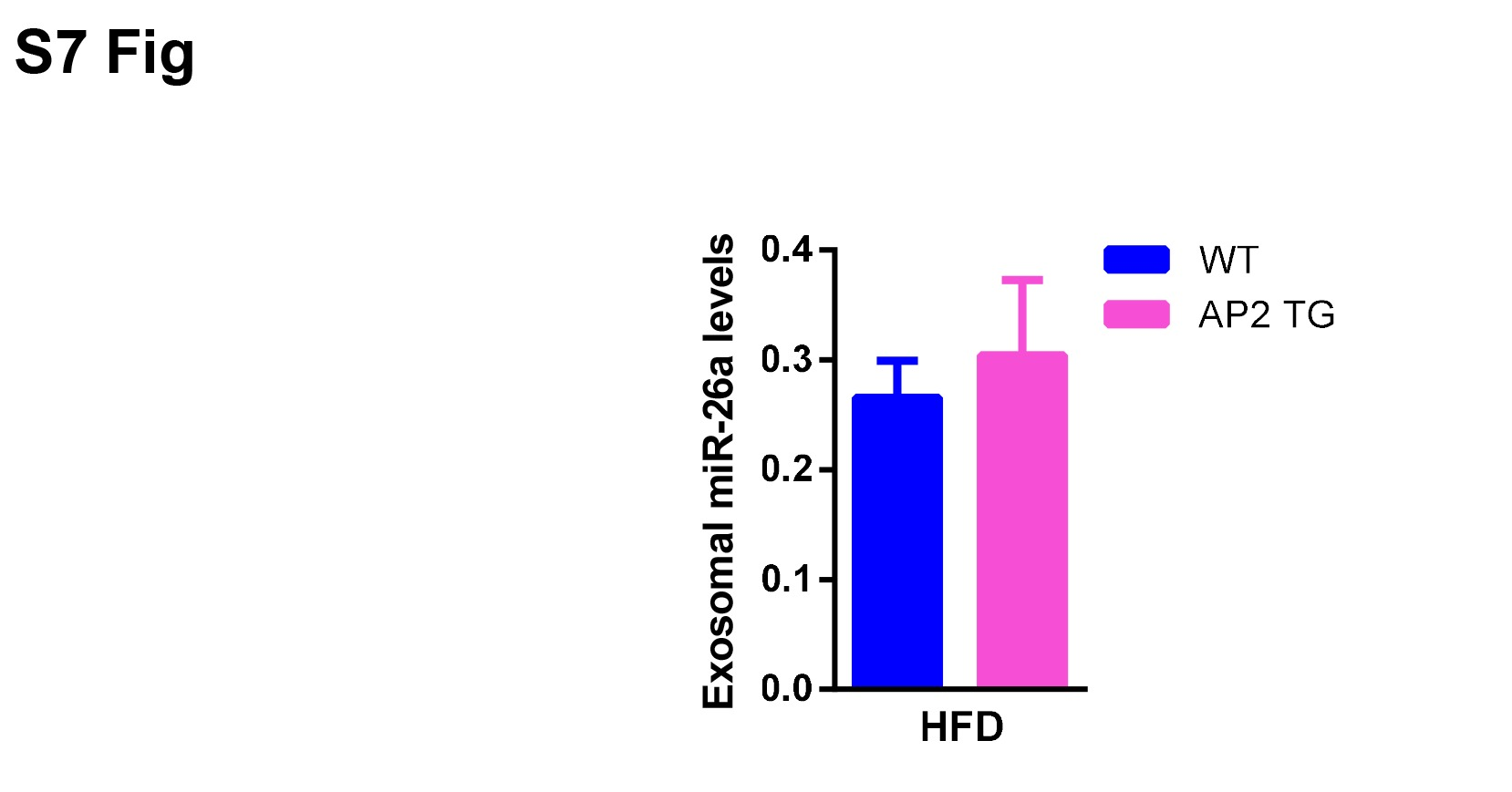

Supplement: S7 Fig — Exosomal miR-26a in the serum of WT and AP2 TG mice fed an HFD (n = 6). The data underlying this figure may be found in S2 Data. Data are shown as mean ± SD. Student t test. AP2 TG, adipocyte-specific miR-26a overexpression mouse; AP2, adipocyte fatty acid binding protein; DIO, diet-induced obese; HFD, high-fat diet; TG, transgenic; WT, wild type (TIF) [file pbio.3000603.s007.tif]

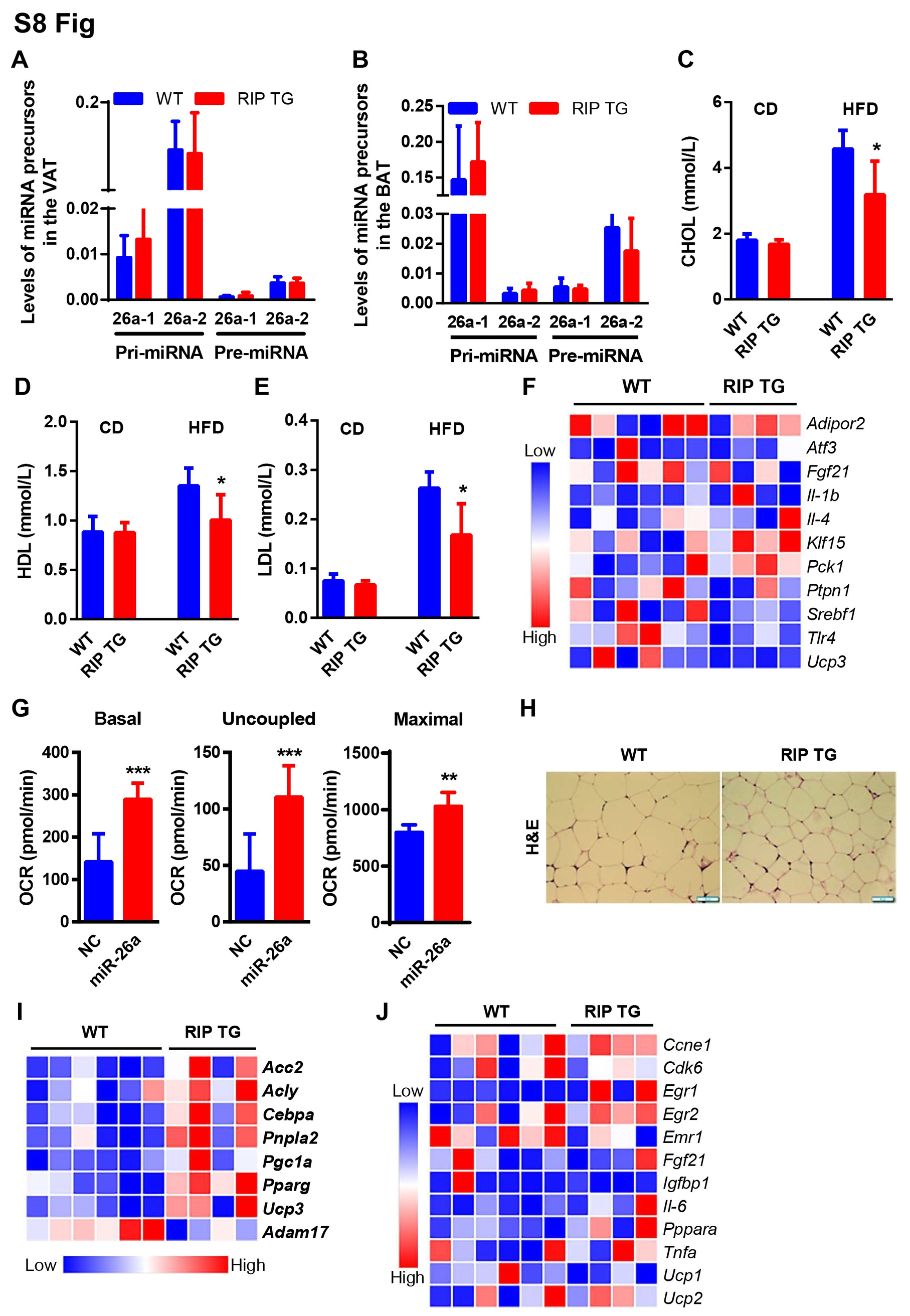

Supplement: S8 Fig — (A–E and H–J) 6- to 8-week–old RIP TG and WT littermate controls were fed an HFD for 16 weeks. (A and B) Expression of pri- and pre-miR-26a in the VAT (A) or BAT (B) (n = 4–6). (C–E) Plasma cholesterol (C), HDL (D), and LDL (E) levels in RIP TG and WT littermate controls fed a CD or an HFD (n = 5–6). (F) Heat map of mRNA levels of hepatic genes involved in liver metabolism and function. Red and blue depict higher and lower gene expression, respectively. Color intensity indicates magnitude of expression differences. Expression of all listed genes was comparable in two mouse groups (n = 4–6). (G) OCR for primary brown adipocytes isolated from WT and transfected with NCs or miR-26a mimics. Basal OCR, uncoupled OCR, and maximal OCR are presented (n = 6–9). (H) Representative HE-stained WAT. (I and J) Heat map of mRNA levels of genes involved in WAT function and metabolism. Differentially (I) or nondifferentially (J) expressed genes were shown separately (n = 4–6). The data underlying this figure may be found in S2 Data. Data are shown as mean ± SD. *P < 0.05, **P < 0.01, ***P < 0.005, Student t test. BAT, brown adipose tissue; CD, chow diet; HDL, high-density lipoprotein; HE, hematoxylin–eosin; HFD, high-fat diet; LDL, low-density lipoprotein; NC, negative control; OCR, oxygen consumption rate; pre-miR-26a, precursor miR-26a; pri-miR-26a, primary miR-26a RIP, rat insulin promoter; TG, transgenic; VAT, visceral adipose tissue; WAT, white adipose tissue; WT, wild type. (TIF) [file pbio.3000603.s008.tif]

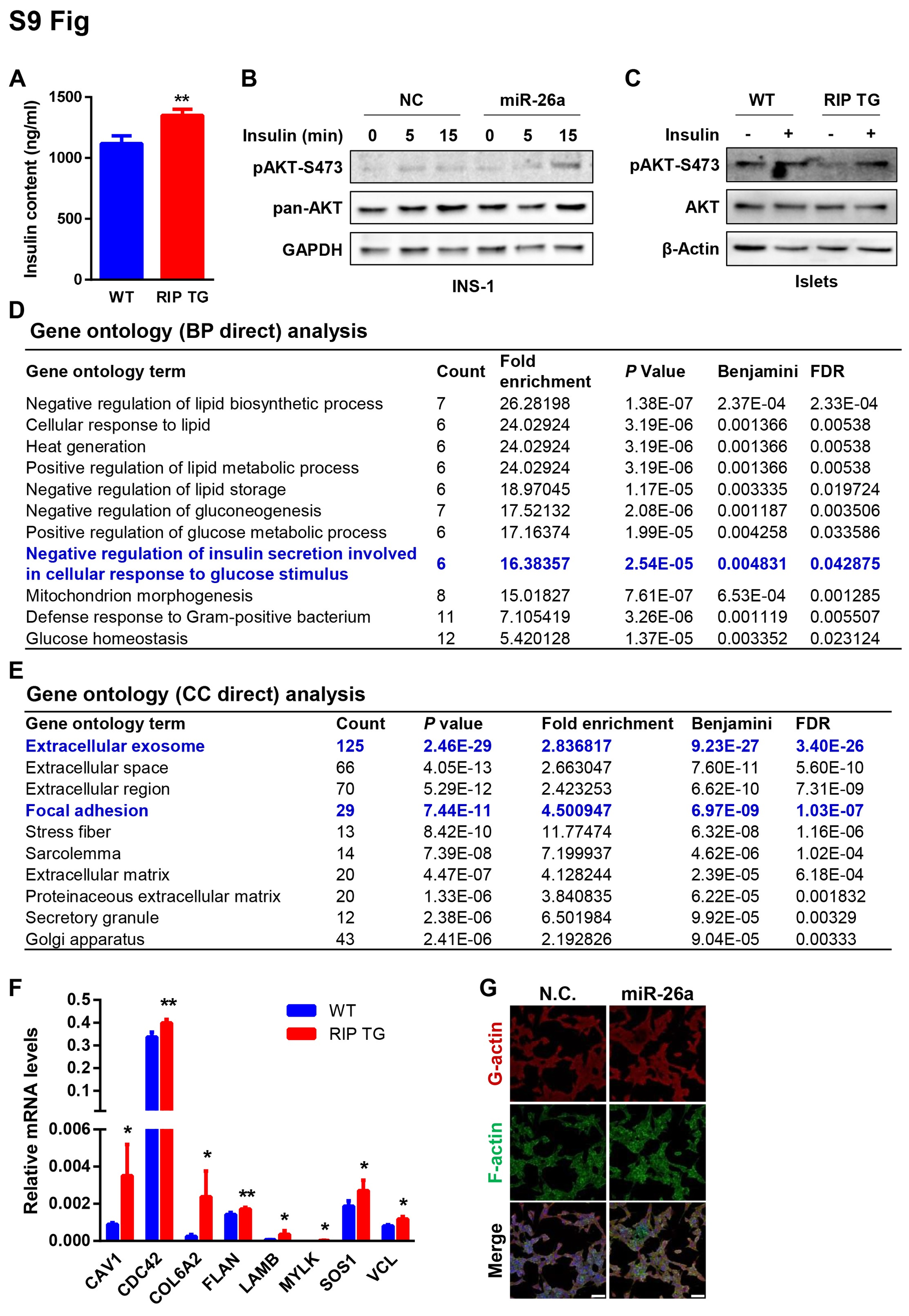

Supplement: S9 Fig — (A) Insulin contents extracted from islets (n = 3). (B) AKT phosphorylation in INS-1 cells that transfected with NCs or miR-26a mimics and treated with insulin (100 nM) for indicated times. (C) Insulin-stimulated AKT phosphorylation in islets isolated from WT and RIP TG mice fed an HFD for 15 weeks. Islets in each group were isolated from 4 mice and pooled together for western blot assay. (D and E) Proteomic analysis on islets of either RIP TG or WT littermate controls fed an HFD for 2 days. Gene ontology analysis of differentially expressed islet proteins between WT littermates and RIP TG mice. (D) Gene ontology (BP direct) analysis. (E) Gene ontology (CC direct) analysis. (F) Certain genes associated with focal adhesin in Fig 6E were verified by QRT-PCR. (G) Representative IF imaging of G-actin and F-actin in Min6 cells (scale bar, 50 μm) (n = 3). The data underlying this figure may be found in S2 Data and S1 Raw Images. Data are shown as mean ± SD. *P < 0.05, **P < 0.01, Student t test. BP, biological process; CC, cellular component; F-actin, filamentous actin; G-actin, globular actin; HFD, high-fat diet; IF, immunofluorescence; INS-1 cells, rat β cells; Min6 cells, murine β cells; NC, negative control; QRT-PCR, quantitative reverse transcriptase PCR; RIP, rat insulin promoter; TG, transgenic; WT, wild type. (TIF) [file pbio.3000603.s009.tif]

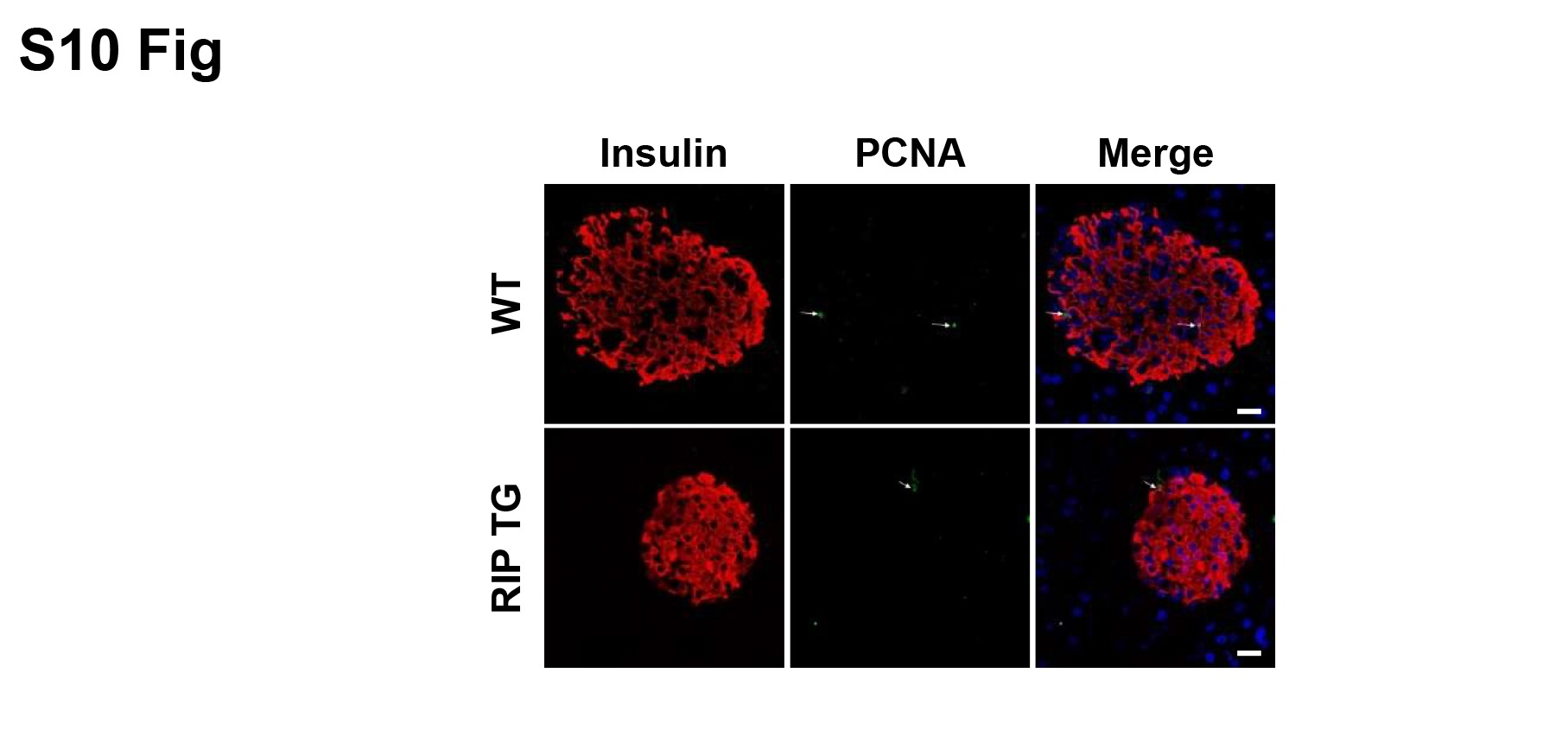

Supplement: S10 Fig — Representative IF staining for insulin and PCNA in pancreas from RIP TG and WT controls fed an HFD for 16 weeks (scale bar, 20 μm) (n = 4). HFD, high-fat diet; IF, immunofluorescence; PCNA, proliferative cell nuclear antigen; RIP, rat insulin promoter; TG, transgenic; WT, wild type. (TIF) [file pbio.3000603.s010.tif]

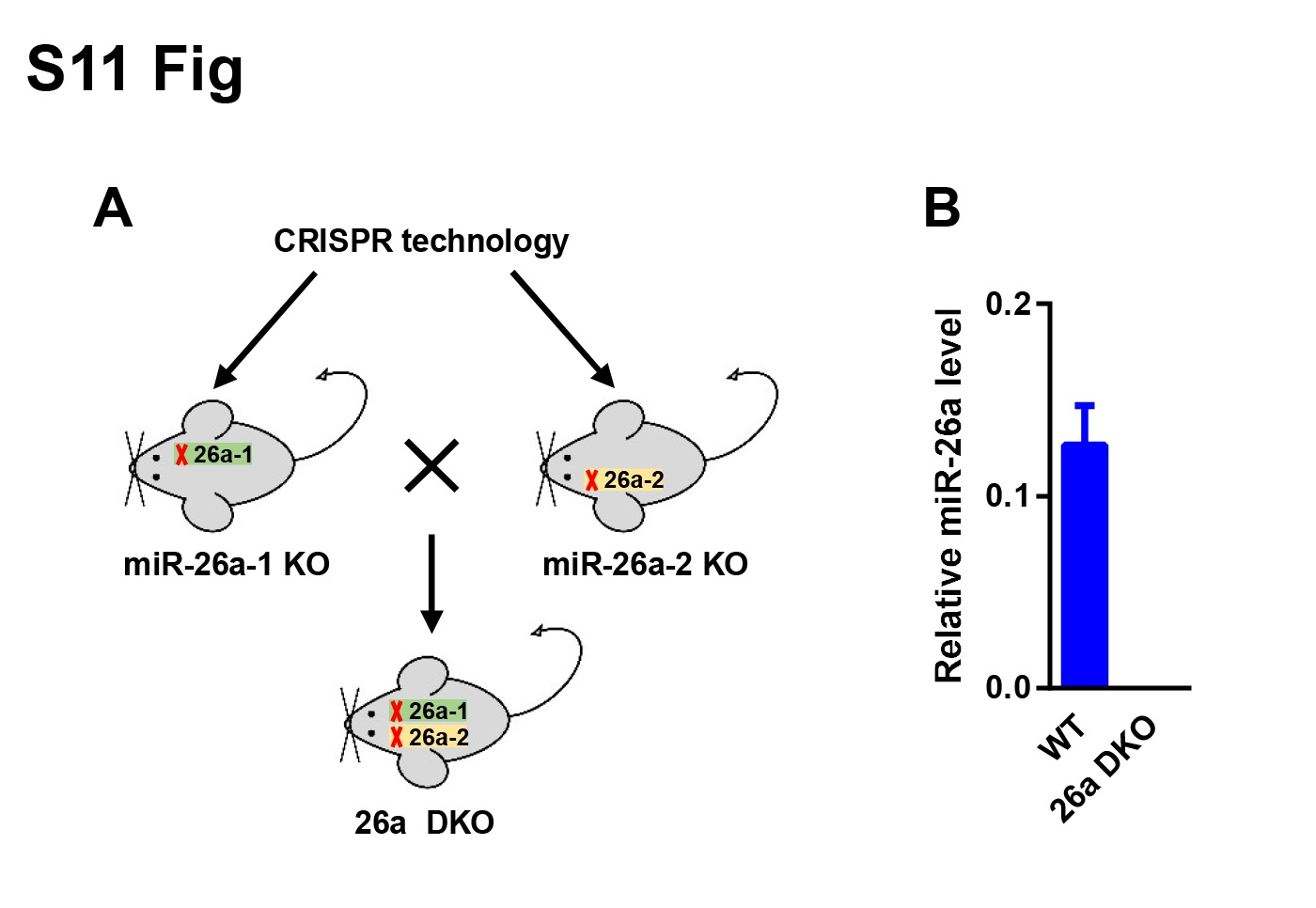

Supplement: S11 Fig — (A) Scheme for generating 26a DKO mice. Knockout mouse lines for miR-26a-1 and miR-26a-2 were separately established by CRISPR technology, and then these two mouse lines were intercrossed to obtain 26a DKO mice. (B) Expression of miR-26a in islets of 26a DKO mice and WT controls (n = 3). The data underlying this figure may be found in S2 Data. Data are shown as mean ± SD. Student t test. WT, wild type; 26a DKO mice, miR-26a double knockout mice. (TIF) [file pbio.3000603.s011.tif]

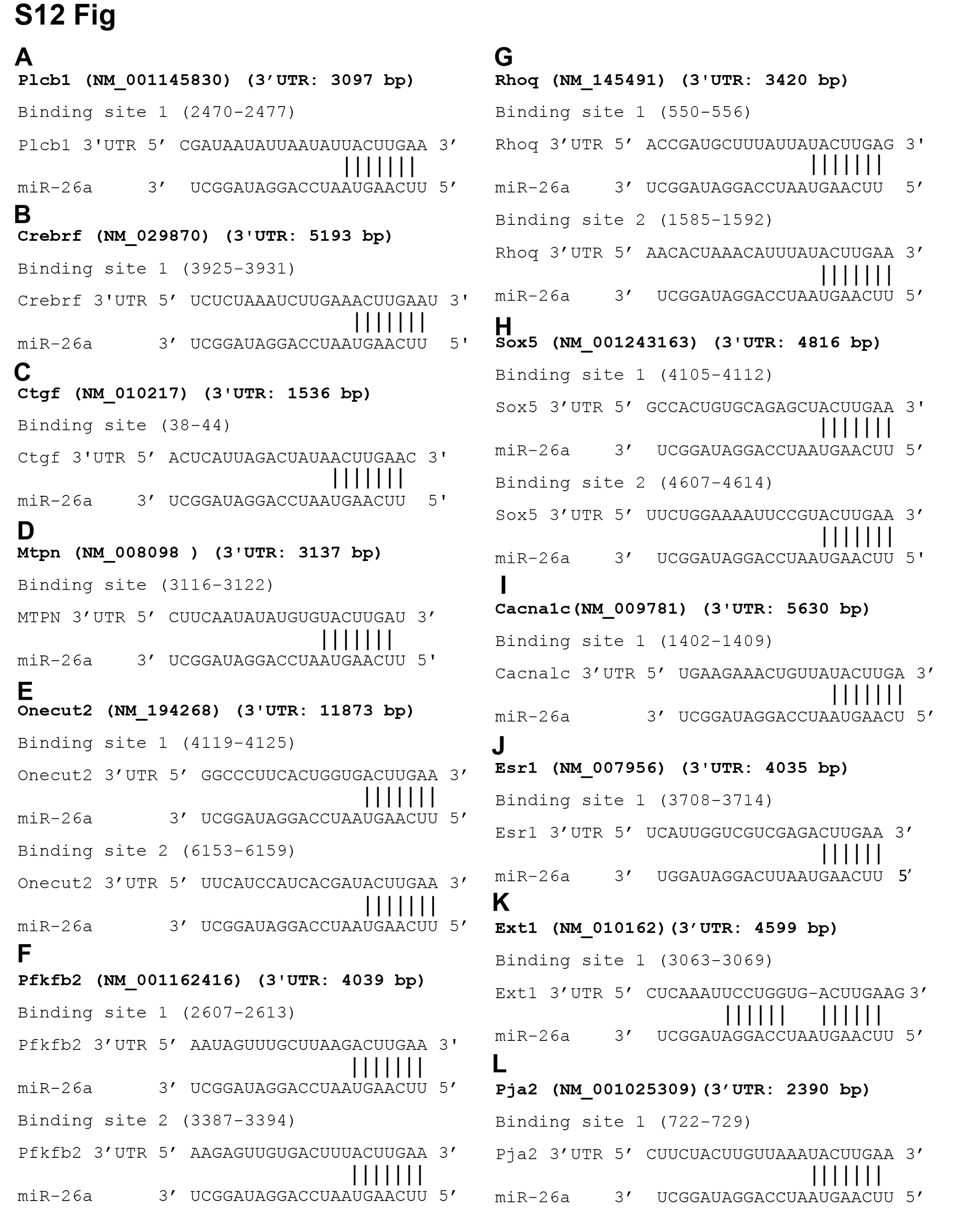

Supplement: S12 Fig — (A–L) Predicted consequential pairing of target region and miR-26a is shown. (TIF) [file pbio.3000603.s012.tif]

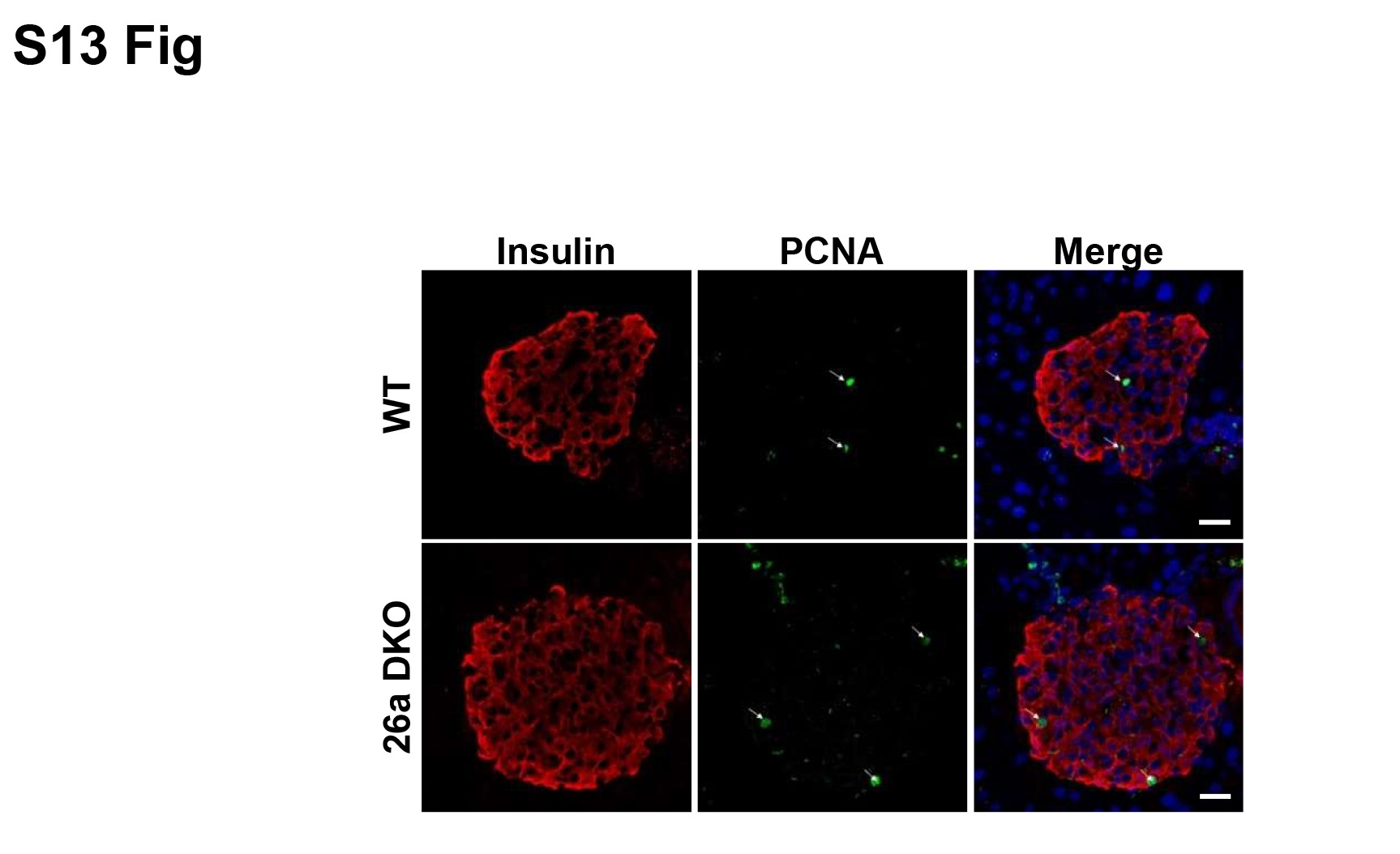

Supplement: S13 Fig — Representative IF staining for insulin and PCNA in pancreas from 26a DKO and WT controls fed an HFD for 8 weeks (scale bar, 20 μm) (n = 4). HFD, high-fat diet; IF, immunofluorescence; PCNA, proliferative cell nuclear antigen; WT, wild type; 26a DKO, miR-26a double knockout (TIF) [file pbio.3000603.s013.tif]

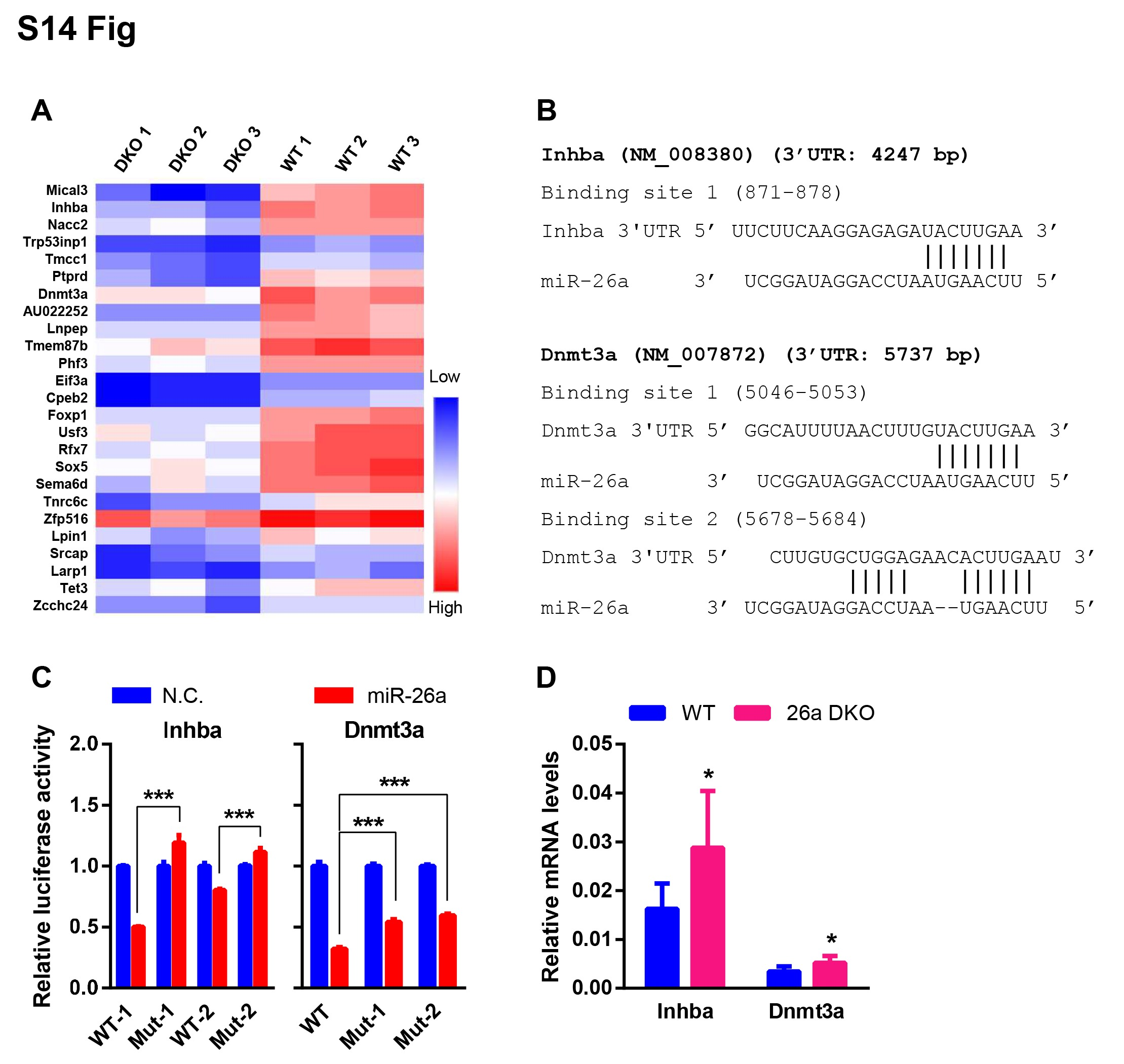

Supplement: S14 Fig — (A) The top 25 enriched hepatic genes in WT mouse identified by RNA immunoprecipitation sequencing are presented in a heat map (n = 3). Red and blue depict higher and lower gene enrichment, respectively. Color intensity indicates magnitude of enrichment differences. (B) Predicted consequential pairing of target region and miR-26a is shown. (C) Relative luciferase activity in 293T cells transfected with reporter constructs containing the 3′ UTR of target genes and co-transfected with either miR-26a mimics or NCs. (D) Expression of miR-26a target genes in livers of WT and 26a DKO mice fed an HFD (n = 5–8). The data underlying this figure may be found in S2 Data. Data are shown as mean ± SD. *P < 0.05, ***P < 0.005, Student t test. HFD, high-fat diet; NC, negative control; WT, wild type; 26a DKO mice, miR-26a double knockout mice. (TIF) [file pbio.3000603.s014.tif]
