# Supplementary material for: Pancreatic β cell microRNA-26a alleviates type 2 diabetes by improving peripheral insulin sensitivity and preserving β cell function
Source: PLoS Biol. 2020 Feb 24;18(2):e3000603. doi: 10.1371/journal.pbio.3000603 (PMC7058362; doi:10.1371/journal.pbio.3000603)
Supplement: S1 Table — miRNA, microRNA. (DOCX) [file pbio.3000603.s015.docx]

**S1 Table. 12 miRNAs examined in this study**

| **miRNA** | **Biological Function** | **Target organ** | **Target genes** | **Species** |
| --- | --- | --- | --- | --- |
| miR-26a | Improve insulin sensitivity | Liver | Gsk3β, Pkcδ, Pkcθ, Pten | Mouse, Human |
| miR-103 | Inhibit insulin sensitivity | Liver | Cav1 | Mouse, Human |
| miR-107 | Inhibit insulin sensitivity | Liver | Cav1 | Mouse, Human |
| miR-143 | Inhibit insulin signaling | Liver | Orp8 | Mouse |
| miR-206 | Increase insulin signaling | Liver | Ptpn1 | Mouse, Human |
| miR-423 | Inhibit insulin sensitivity | Liver | Fam3A | Human |
| miR-802 | Inhibit insulin sensitivity | Liver | Hnf1b | Mouse, Human |
| let-7 | Impair insulin signaling | Muscle | Insr, Irs2, Igf1r | Mouse, Human |
| miR-29a family | Impair insulin signaling | Muscle | Mct1, Irs1, Col3a1 | Mouse, Human |
| miR-126 | Inhibit insulin resistance | Adipose | Ccl2 | Mouse |
| miR-221 | Impair insulin sensitivity | Adipose | Adipor1, Ets1 | Mouse |
| miR-328 | Improve insulin sensitivity | Adipose | Bace1 | Mouse |
