# Supplementary material for: Pancreatic β cell microRNA-26a alleviates type 2 diabetes by improving peripheral insulin sensitivity and preserving β cell function
Source: PLoS Biol. 2020 Feb 24;18(2):e3000603. doi: 10.1371/journal.pbio.3000603 (PMC7058362; doi:10.1371/journal.pbio.3000603)
Supplement: S3 Table — The proteins with differential expressions (P < 0.05) in islets of RIP TG mice compared with their WT littermates are listed below. RIP, rat insulin promoter; TG, transgenic; WT, wild type. (DOCX) [file pbio.3000603.s017.docx]

**S3 Table. Proteomics analysis on islets**

| **Upregulated in RIP TG** | Aspn; Prelp; Tagln; Matn2; Tpm2; Bgn; Col6a5; Pgm5; Cnn1; Fbn1; Cdh13; Myl9; Myl12a; Enpp2; Myh11; Tpm1; Col1a1; Gbp9; Mpa2l; Gbp10; Gbp6; Dcn; Chgb; Ybx1; Trappc2; Fhl1; Magp1;Mfap2; App; Glipr2; Ern1; Ano6; Apom; Tpm1; Adipoq; Actn4; Mup1; Mup11; Mup14; Mup13; Mup15; Mup6; Mup10; Mup4; Mup8; Mup9; Mup5; Mup2; Mup17; Prkcdbp; Cnn2; Flna; Colgalt1; Ap3m2; Krt10; Slco2a1; Col6a3; Palld; Slc2a13; Dnajc10; Col6a1; Ckb; Mgat3; Hspg2; Pdlim7; Alb; Plin2; Asxl2; Cald1; Ndufaf4; Srbd1; Ptrf; Anxa1; Mgst1; Golga1; Pdlim3; Cav1; Strc; Chga; Des; Tgm2; Fmo1; Hapln4; Rras; Slc12a9; Susd2; F13a1; Eps15; Cps1; Myof; Rbp1; Anxa2; Arl2; Pcbp3; Col6a2; Nrbp1; Csrp1; Serpina3h;Serpina3n; Banf1; Bcam; C530008M17Rik; Kiaa1211; Slmap; Col6a6; St7; Cald1; Ly6e; Mylk; Sorbs2; Ptgs1; Pdp2; Sh3gl2; Ahnak; Aoc3; Fmo2; Dpy19l1; Sdpr; Enpp1; Hist1h1c; Pcsk1; Serpina1b; Serpina1a; Serpina1c; Serpina1e; Serpina1d; Apba3; Prg4; Lamb2; Emc3; Gaa; Lamc1; Lpp; Ppp1r1b; Ptms; Hist1h1d; Ehd2; Cr1l; Anxa3; Gbp2; Gbp2b; Gbp1; Plvap; Ceacam1; Ceacam2; Ifrd1; Arglu1; Vim; Crip1; Reg3b; Amph; Tceal3; Tceal6; Tceal5; Ddc; Sos1; Sos2; Ppap2b; Rqcd1; Atp8a1; Timm21; Arrb1; Zfr; Gclc; H2-D; H2-D1; Armcx3; Fabp3; Fabp7; Plat; Ptgr1; Vcl; Cnn3; Stam; Gsta4; Rabep1; Rtn4; Sars2; Ppap2a; Pnkp; Nbeal1; Ddah2; Nccrp1; Ccdc97; Ctsl; Inmt; 2410002F23Rik; Ppl; Acyp2; Rtca; RtcA; Cd36; Serpinb9; Aqp1; Dnah12; Ecm1; Hist1h1d; Cpped1; Tppp3; Sntb2; Chst11; Chst13; Pecam1; Capg; Commd9; Pfkp; Krt76; Ezr; Pdcd5; Sart1; S100a11; Brwd3; Sorbs1; H1f0; Lyz1; Lyz2; Nucks1; Jmy; Pclo; Pck2; Sucla2; Mff; Rbm47; Qrich1; Dtx3l; Ager; Derl3; Clasp1; C1rb;C1ra; Mcf2l; Cyp7b1; Fam171a2; Mrpl53; Hist1h1b; Lmcd1; Aga; Stk38 |
| --- | --- |
| **Downregulated in RIP TG** | Sult1c2; Gatm; Nt5dc2; Tsc1; Pex6; Sys1; Agr2; Ptpmt1; 2010107G23Rik; Fip1l1; Slc30a5; Ttr; Mfsd10; Coq6; St3gal-1; St3gal1; Bet1; Fkbp2; Reep3; Lrrc1; Slc35c1; Cltb; Epb4.1; Isca2; Khk; Gc; Alyref; Folr1; Rbmxl1; Clic5; Hbbt1; Hbb-bs; Txndc17; Spink3; Cdc42; Eif2s3y; Syt7; Grwd1; Ndufa1; Wdr3; Ero1lb; Ctsc; Pdyn; Uaca; Abhd10; Rps29; Gm10126; Ndufab1; Rsrc2; Abcb8; Stx3; Zcchc11; Gde1; Syt1; Athl1; Calb1; Tmem206; Pex11b; Slc39a11; Rcn1; Akap1; Clpp; Arhgef18; Slc37a4; Clic6; Ppapdc1b; Slc35b4; Ppp3ca; Narfl; Atpaf2; Coa6; Wdr18; Lss; Ucn3; Cox20; mKIAA1589; Zfyve1; Cnot6l; haemaglobin alpha 2; Hbat1; Hba; Slc2a2; Gfap; Nucb1; Sst; A1cf; Ugt2b34; Mark3; Mark1; Clta; Tmem238; Elovl1; Stk16; Plp2; Calm1; Calml3; Pcsk1n; Yipf5; Golm1; Pafah2; Saraf; Mcfd2; Ndufs6; Prkdc; Nupr1; Gcg; Atp6v0a2; Chp1; Elp5; Pappa2; Srrm1; Cpne9; Cpne5 |
