# Supplementary material for: Pancreatic β cell microRNA-26a alleviates type 2 diabetes by improving peripheral insulin sensitivity and preserving β cell function
Source: PLoS Biol. 2020 Feb 24;18(2):e3000603. doi: 10.1371/journal.pbio.3000603 (PMC7058362; doi:10.1371/journal.pbio.3000603)
Supplement: S7 Table — (DOCX) [file pbio.3000603.s021.docx]

**S7 Table. Primers for** **genotyping**

| **Primer** | **Forward (5’–3’)** | **Reverse (5’–3’)** |
| --- | --- | --- |
| **Genotype Identification of RIP TG mice** | | |
| miR-26a | GCAGAAACTCCAGAGAGAAGGA | CCAAAAGACGGCAATATGGTGGAA |
| Cre | CAGCATTGCTGTCACTTG GTC | ATTTGCCTGCATTACCGGTCG |
| **Genotype Identification of 26a DKO Mice** | | |
| miR-26a-1 | TCAGGAGGACTGCCCAAGAA | GCTACAGGCAAAGGGTTGGA |
| miR-26a-2 | ACTGGGTGGCGAGTTAGTTG | CGAGACACTGAGACCTCTGC |
