# Supplementary material for: Pancreatic β cell microRNA-26a alleviates type 2 diabetes by improving peripheral insulin sensitivity and preserving β cell function
Source: PLoS Biol. 2020 Feb 24;18(2):e3000603. doi: 10.1371/journal.pbio.3000603 (PMC7058362; doi:10.1371/journal.pbio.3000603)
Supplement: S8 Table — QRT-PCR, quantitative reverse transcriptase PCR. (DOCX) [file pbio.3000603.s022.docx]

**S8 Table. Primers for QRT-PCR**

| **Gene** | **Forward (5’ – 3’)** | **Reverse (5’ – 3’)** |
| --- | --- | --- |
| Pri-26a-1 | TCAGGAGGACTGCCCAAGAA | CAAAGGGTTGGAGATGGACG |
| Pri-26a-2 | GACCTACACAAGCCGTCCT | TCCCAGGAGCCATCTTCATT |
| Pre-26a-1 | AAGGCCGTGGCCTCGTTCAAGT | CAGGCCCGCGTCCCCGTGCAAG |
| Pre-26a-2 | GGCTGCGGCTGGATTCAAGTAA | CGCTGCCTCCAGAAACAAGTAA |
| Acc1 | ATGGGCGGAATGGTCTCTTTC | TGGGGACCTTGTCTTCATCAT |
| Acc2 | ACAGAGATTTCACCGTTGCGT | CGCAGCGATGCCATTGT |
| Acly | GAATGCTGGACAACATCCTG | GTTCATTAGACATGCCTCCTG |
| Acsl3 | GCTCTGAGGAGCAGCCAG | TGGTGTTGAAGATACGTGGTT |
| Adam17 | AGGACGTAATTGAGCGATTTTGG | TGTTATCTGCCAGAAACTTCCC |
| Adioq | GCACTGGCAAGTTCTACTGCAA | GTAGGTGAAGAGAACGGCCTTGT |
| Adipor1 | AATGGGGCTCCTTCTGGTAAC | GCAGACCTTATACACGAACTCC |
| Adipoq | TGTTCCTCTTAATCCTGCCCA | CCAACCTGCACAAGTTCCCTT |
| Atf3 | GAGGATTTTGCTAACCTGACACC | TTGACGGTAACTGACTCCAG |
| Cav1 | GCGACCCCAAGCATCTCAA | ATGCCGTCGAAACTGTGTGT |
| Ccne1 | GTGGCTCCGACCTTTCAGTC | CACAGTCTTGTCAATCTTGGCA |
| Cd36 | ATGGGCTGTGATCGGAACTG | GTCTTCCCAATAAGCATGTCTCC |
| Cdk6 | GGCGTACCCACAGAAACCATA | AGGTAAGGGCCATCTGAAAACT |
| Cebpa | CAAGAACAGCAACGAGTACCG | GTCACTGGTCAACTCCAGCAC |
| Cidea | TGCTCTTCTGTATCGCCCAGT | GCCGTGTTAAGGAATCTGCTG |
| Cox5b | GGAAGACCCTAATCTAGTCCCG | GTTGGGGCATCGCTGACTC |
| Cox7a | CAGCGTCATGGTCAGTCTGT | AGAAAACCGTGTGGCAGAGA |
| Cox8b | GAACCATGAAGCCAACGACT | GCGAAGTTCACAGTGGTTCC |
| Cpt1a | CTCCGCCTGAGCCATGAAG | CACCAGTGATGATGCCATTCT |
| Cpt1b | GCACACCAGGCAGTAGCTTT | CAGGAGTTGATTCCAGACAGGTA |
| Cpt2 | CAGCACAGCATCGTACCCA | TCCCAATGCCGTTCTCAAAAT |
| Crebrf | AGCGTAAGCGGAATGGACC | CAGGACATCTGTGAAAGTCTCC |
| Crem | CGGCCACCTTCTACAGCTC | GGAGGATCACCTGTCTATTCTCA |
| Ctgf | GGGCCTCTTCTGCGATTTC | ATCCAGGCAAGTGCATTGGTA |
| Dio2 | AATTATGCCTCGGAGAAGACCG | GGCAGTTGCCTAGTGAAAGGT |
| Dyrk1a | TGCACCGTCGTTCTCATTCC | CTGGTCACTTATGCTCGGCT |
| Emr1 | TGACTCACCTTGTGGTCCTAA | CTTCCCAGAATCCAGTCTTTCC |
| Egr1 | TCGGCTCCTTTCCTCACTCA | CTCATAGGGTTGTTCGCTCGG |
| Egr2 | GCCAAGGCCGTAGACAAAATC | CCACTCCGTTCATCTGGTCA |
| Fabp4 | AAGGTGAAGAGCATCATAACCCT | TCACGCCTTTCATAACACATTCC |
| Fasn | GGCATCATTGGGCACTCCTT | GGGTGTGGCTCAGGAGGAT |
| Fgf21 | CTGCTGGGGGTCTACCAAG | CTGCGCCTACCACTGTTCC |
| Foxo1 | CCCAGGCCGGAGTTTAACC | GTTGCTCATAAAGTCGGTGCT |
| Foxm1 | ATCGCTACTTGACATTGGACCA | GATTGGGTCGTTTCTGCTGTG |
| Gsk3β | GACCGAGAACCACCTCCTTT | GTGGTTACCTTGCTGCCATC |
| Gys2 | GCTCTCCAGACGATTCTTGCA | GTGCGGTTCCTCTGAATGATC |
| Hes1 | CCAGCCAGTGTCAACACGA | AATGCCGGGAGCTATCTTTCT |
| Igfbp1 | ATCAGCCCATCCTGTGGAAC | TGCAGCTAATCTCTCTAGCACTT |
| Il1b | GCAACTGTTCCTGAACTCAACT | ATCTTTTGGGGTCCGTCAACT |
| Ins1 | CCTGTTGGTGCACTTCCTAC | TGCAGTAGTTCTCCAGCTGG |
| Ins2 | CGTGGCTTCTTCTACACACCC | AGCTCCAGTTGTGCCACTTGT |
| ki67 | ATCATTGACCGCTCCTTTAGGT | GCTCGCCTTGATGGTTCCT |
| Leptin | GAGACCCCTGTGTCGGTTC | CTGCGTGTGTGAAATGTCATTG |
| Ldlr | GCATCAGCTTGGACAAGGTGT | GGGAACAGCCACCATTGTTG |
| Neourod1 | ATGACCAAATCATACAGCGAGAG | TCTGCCTCGTGTTCCTCGT |
| Nkx2.2 | CCGGGCGGAGAAAGGTATG | CTGTAGGCGGAAAAGGGGA |
| Nkx6.1 | TCAGTCAAGGTCTGGTTCC | CGATTTGTGCTTTTTCAGCA |
| MafA | AGGAGGAGGTCATCCGACTG | CTTCTCGCTCTCCAGAATGTG |
| Mtpn | CCCTGAAAAACGGAGACTTGG | GAAACATGACCCTCATAGACAGC |
| Onecut2 | ACACCACGCCATGAGTATGTC | GCGTCAGCGTAGTGTAGGT |
| Pck1 | CTGGATGAAGTTTGATGCCC | TGTCTTCACTGAGGTGCCAG |
| Pdx1 | TTCCCGAATGGAACCGAGC | GTAGGCAGTACGGGTCCTCT |
| Pfkfb2 | CGCCTCAAATCTGCGAAGG | ACAATGAGTGTTGGGGAGTTG |
| Pgc1a | TGAGGACCGCTAGCAAGTTT | TGAAGTGGTGTAGCGACCAA |
| Pnpla2 | ATGTTCCCGAGGGAGACCAA | GAGGCTCCGTAGATGTGAGTG |
| Ppara | TATTCGGCTGAAGCTGGTGTAC | CTGGCATTTGTTCCGGTTCT |
| Pparg | TGAGGACCGCTAGCAAGTTT | TGAAGTGGTGTAGCGACCAA |
| Prdm16 | ACGGATGTACTTGAGCCAGC | CAGAGGTGTCATCCCAGGAG |
| Ptpn1 | GGAACTGGGCGGCTATTTACC | CAAAAGGGCTGACATCTCGGT |
| Pygl | GAAGGAGGCAAACGGATCAAC | TCACGATGTCCGAGTGGATCT |
| Retn | AAGAACCTTTCATTTCCCCTCCT | GTCCAGCAATTTAAGCCAATGTT |
| Rhoq | GTACGTGCCCACTGTCTTCG | GGCCTCAGACGATCATAGTCTT |
| Sox5 | AGCCGCAATGCAGGTTTCT | TTGTGCTCTTGTCTGTGTGAAT |
| Srebf1 | TGACCCGGCTATTCCGTGA | CTGGGCTGAGCAATACAGTTC |
| Stx3 | GAAGGCACGGGATGAAACTAA | GGACAGTCCAATAATCAACGCTA |
| Scd1 | TTCTTGCGATACACTCTGGTGC | CTTCACCTTCTCTCGTTCATTTCC |
| Slc2a4 | GTGACTGGAACACTGGTCCTA | CGGGATTGAATGTTCTTGTCGT |
| Tcf7l2 | AACGAACACAGCGAATGTTTCC | CTCGGCATTTCTTAGGAGCG |
| Tle4 | TTTACAGGCTCAATACCACAGTC | TGCACAGATAGCATTTAGTCGTT |
| Tlr4 | ATGGCATGGCTTACACCACC | GAGGCCAATTTTGTCTCCACA |
| Tnf | CCCTCACACTCAGATCATCTTCT | GCTACGACGTGGGCTACAG |
| Trim14 | GTGCGTGTGCAGAAGCTAATC | CTGCGTAAACCTTGAGCCTTT |
| Ucp1 | TCAGCTGTTCAAAGCACACA | GTACCAAGCTGTGCGATGTC |
| Ucp3 | CTGCACCGCCAGATGAGTTT | ATCATGGCTTGAAATCGGACC |
| Cdc42 | TTTCTTGCTTGTTGGGACCC | TAGGCCTTTCTGTGTGAGGG |
| Vcl | GCAAAGCCATTCCTGACCTC | CAGTCTCTTTTCCAACCCGG |
| Lamb2 | CGGAAGGCGAGGTCATCTAT | GCAAGTTGTCTCCCAGTGTG |
| Col6a2 | TGGACAGAAAGGACGACAGG | TTCCGACCATCCGATCCAAA |
| Cav1 | CGTAGACTCCGAGGGACATC | TTGCTTCTCAGTCACCTCGT |
| Flna | GCTTGAATTCCTGGACCGTG | GCCTCCTCATCCTCTTCCTC |
| Mylk | CGGCTATCATCTCCTGCTCT | TTGTCCCTCCTGCTTTGACT |
| Sos1 | TCAGATGTGGAGGAACGTGT | GCTGGCAGCGATAAAGGATT |
| Plcb1 | TGTCCGACAGCCTCAAGAAG | AGCTCCGTCTCCTTATTCTGG |
| Pja2 | GTGATAGTAGTGGCCCCGAG | TGCTGCTCTCATTGACCTCA |
| Ext1 | GACTGGCAAAAGCACAAGGA | CTGTGTCTGCTGTCTAAGTGC |
| Esr1 | TGCACCATTGACAAGAACCG | CATCTCTCTGACGCTTGTGC |
| Cacna1c | GGAGGGAAAGGAGCTGGATT | GACGAAGAGCACAAGAAGGG |
| Dnmt3a | GAGAATGGCTGCTGTGTGAC | TGGAAGGTGAGTCTTGGCAT |
| Inhba | CATCACCTTTGCCGAGTCAG | GGTCCTGGTTCTGTTAGCCT |
| m36b4 | AGATGCAGCAGATCCGCAT | GTTCTTGCCCATCAGCACC |
| Cyclophilin A | CAGGTCCTGGCATCTTGTCC | TGGCTTCCACAATGTTCATGC |
| Gapdh | TGGCAAAGTGGAGATTGTTGCC | AAGATGGTGATGGGCTTCCCG |
| U6 | CGCTTCGGCAGCACATATAC | AGGGGCCATGCTAATCTTCT |
