# Supplementary material for: Pancreatic β cell microRNA-26a alleviates type 2 diabetes by improving peripheral insulin sensitivity and preserving β cell function
Source: PLoS Biol. 2020 Feb 24;18(2):e3000603. doi: 10.1371/journal.pbio.3000603 (PMC7058362; doi:10.1371/journal.pbio.3000603)
Supplement: S9 Table — (DOCX) [file pbio.3000603.s023.docx]

**S9 Table. Antibodies used in this study**

| **Antibodies Source Cat. No** | | |
| --- | --- | --- |
| TSG101 Rabbit Polyclonal antibody | Proteintech | 14497-1-AP |
| CD63 Rabbit Polyclonal antibody | Proteintech | 25682-1-AP |
| Phospho-Insulin/IGF Receptor Antibody Sampler Kit | Cell Signaling | 8338 |
| PhosphoPlus® Akt (Ser473) Antibody | Cell Signaling | 8200 |
| Pan-Akt Antibody | Cell Signaling | 4691 |
| Phospho-PTK2-Y397 pAb | ABclonal | AP0302 |
| PTK2 Polyclonal Antibody | ABclonal | A11195 |
| Antiphospho-ERK1/2 (Thr202/Tyr204) | Cell Signaling | 4377 |
| ERK1/2 Rabbit Polyclonal Antibody | Proteintech | 16443-1-AP |
| Caveolin-1 Rabbit Polyclonal antibody | Proteintech | 16447-1-AP |
| FLNA Polyclonal Antibody | ABclonal | A16376 |
| Anti-pan Ago, clone 2A8 | Millipore | MABE56 |
| GAPDH Monoclonal Antibody | ThermoFisher | 31430 |
| Actin Monoclonal Antibody | ThermoFisher | 31460 |
| Beta Tubulin Rabbit Polyclonal Antibody | Proteintech | 10068-1-AP |
| Insulin Rabbit Monoclonal Antibody | Abcam | 181547 |
| Insulin Mouse Monoclonal Antibody | HUABIO | EM80714 |
| Glucagon Mouse Monoclonal Antibody | Abcam | 10988 |
| PCNA Recombinant Rabbit Monoclonal Antibody | HUBIO | ET1605-38 |
| Alexa Fluor® 488 Anti-Mouse IgG (H+L) | Jackson ImmunoResearch | 120901 |
| Alexa Fluor® 594 Anti-Rabbit IgG (H+L) | Jackson ImmunoResearch | 120330 |
| Phalloidin-iFluor 488-Cyto Painter | Abcam | 176753 |
| Deoxyribonuclease I, Alexa Fluor™ 594 Conjugate | Invitrogen | D12372 |
