# Supplementary material for: Pancreatic β cell microRNA-26a alleviates type 2 diabetes by improving peripheral insulin sensitivity and preserving β cell function
Source: PLoS Biol. 2020 Feb 24;18(2):e3000603. doi: 10.1371/journal.pbio.3000603 (PMC7058362; doi:10.1371/journal.pbio.3000603)
Supplement: S10 Table — (DOCX) [file pbio.3000603.s024.docx]

**S10 Table. Primers for plasmid construction**

| **Primer** | **Forward (5’–3’)** | **Reverse (5’–3’)** |
| --- | --- | --- |
| Crebrf | TCCCATTCCCAAACCTTAACTC | AAACGAAACCACCATCCCTACT |
| Ctgf | GACCTGTGCCTGCCATTACAAC | AATCGGACCTTACCCTGAGCC |
| Mtpn | GTTTCCACAATGTCATTCCCTT | AGCAGCATCTAGTATCGTTAGCTCTT |
| Onecut2 | GCGTTGCTTCCAACAGGGTTATT | GTCTTTGGATTTCGCTTTAGTTATG |
| Pfkfb2 | GTAGGGAGACACGAGTAGAAA | GAAGGGTAAGGATACAGAAGG |
| Rhoq | AGATCCAGAGTGGCGAGATAACC | GAAGACATACTGTGCCCACTTCC |
| Sox5 | GAAGGGACGAGAAGAAAGGAG | TGCCCATCTGTCTTTCTACCG |
| Crebrf-mut | ATCTTGAAACAACAATTTTGAGAATGCATTG | TTAGAGAGATGCTAACATTC |
| Ctgf-mut | AGACTATAACAACAACTGAGTTGCATCTC | AATGAGTTCGTGTCCCTTAC |
| Mtpn-mut | TGGAATAAACGAATATTGTGCTATAGATAACATGTTAG | TTTGTTCTTGCAACACGAAAC |
| Onecut2-mut1 | CCATTTGGACAACAATATAGCCTACAGTC | TTATTTTCTCTGAGACTGTTATTTAAAATAAAAAATTTAAAG |
| Onecut2-mut2 | AGGTGGTTACAACAACTGTGGATGTC | GGGGAAACTGCTCTTCTTAAG |
| Pfkfb2-mut1 | AGGGAATGGAGAATAAAGAGCAACTGAAAG | GACTTTCTCATTCAGTGTTG |
| Pfkfb2-mut2 | TGTGACTTTAGAATAAGTCATTTCATCCAGTCTC | ACTCTTCTGGCATTACCTTAG |
| Rhoq-mut | ACATTTATACAACAAAATGACAGCCTTAAATG | TTAGTGTTCCATTACCCAATC |
| Sox5-mut | GAGCCGAGGAAGGCAAGAAGCCCTGTCC | ACCACAGTCTGTTGGCCC |
| Plcb1 | cgctcgaggagctcgaattcCCAAACACACTGGTGGTTATGG | cgcgtcgacactagtcccggACTGATTTACACGGCCCAACC |
| Pja2 | cgctcgaggagctcgaattcCTCTTCAGTGAGCACTGGATGTG | cgcgtcgacactagtcccggCTGGTTTCACTCGTGAGCGAC |
| Ext1 | cgctcgaggagctcgaattcCTTGCCACAGAACTCATTCCAA | cgcgtcgacactagtcccggACATTCCTTTCTCTTCCGGTGT |
| Esr1 | cgctcgaggagctcgaattcGCACTGTGCTTCGGAGACTTAAG | cgcgtcgacactagtcccggGGTCACATCAATAGCAGTGTGCA |
| Cacna1c | cgctcgaggagctcgaattcCAAGAACAAACCACTGTCTCTGCT | cgcgtcgacactagtcccggCAACCACGTACAGAAGAAGCACTT |
| Dnmt3a | cgctcgaggagctcgaattcAGTCTGCTGTCTGGACAATGATGT | cgcgtcgacactagtcccggACAAAAGGACATTATGACGGTGC |
| Inhba-1 | cgctcgaggagctcgaattcCCACACACACAAACACAGAGGTG | cgcgtcgacactagtcccggGAAGTGCAGCTCTTAAACCCCA |
| Inhba-2 | cgctcgaggagctcgaattcTTGGGAGCTATGCCAGACTGA | cgcgtcgacactagtcccggTCAACCATTGTCAATGCACTGTT |
| Plcb1-mut | TAATATTATGGACAAATAGACAAAGGTAAAGAAAAGGGGT | TCTATTTGTCCATAATATTAATATTATCGATGGATAGTATCATGG |
| Pja2-mut | GTTAAATATGGACAGGTCCAGGACACTCTTGACATGTT | TGGACCTGTCCATATTTAACAAGTAGAAGCAGACTGGATCG |
| Ext1- mut | TGCTTCTAATGACAGGAACCACAAAAAGGGTGAGTTATAG | GGTTCCTGTCATTAGAAGCAGACATTTAATATCAGAAGCC |
| Esr1-mut | GTCGAGATGGACATTAATAAGTGACATCACGCCAGTTTAT | TTATTAATGTCCATCTCGACGACCAATGACCTCTCTGTGA |
| Cacnca1c-mut | CTGTTATATGGACATTCAGGTCAGTTTCAGTATTTTTCAA | CCTGAATGTCCATATAACAGTTTCTTCAACAGAAACATTATC |
| Dnmt3a-mut1 | ACTTTGTATGGACAAACTAAGTGAGCGATTTCACGTGTTT | TTAGTTTGTCCATACAAAGTTAAAATGCCTTTTTAATAATAATA |
| Dnmt3a-mut2 | GGAGAACATGGACATAAATGGACTGTTTTTGTGCAAAAAG | CATTTATGTCCATGTTCTCCAGCACAAGTACATTAGAAAG |
| Inbba-1-mut | GAGAGATATGGACAAGGAATATGTTTGTCCATCTGTTGGA | ATTCCTTGTCCATATCTCTCCTTGAAGAAAATAAAAATTAATC |
| Inbba-2-mut | GTGTCATGGACAAAATATGATTTCCTGTGGGCCTTTTGG | TCATATTTTGTCCATGACACAATCATTTGAATGCATGTTA |
