# Supplementary material for: Pancreatic β cell microRNA-26a alleviates type 2 diabetes by improving peripheral insulin sensitivity and preserving β cell function
Source: PLoS Biol. 2020 Feb 24;18(2):e3000603. doi: 10.1371/journal.pbio.3000603 (PMC7058362; doi:10.1371/journal.pbio.3000603)

Original WB image in Figure 3B

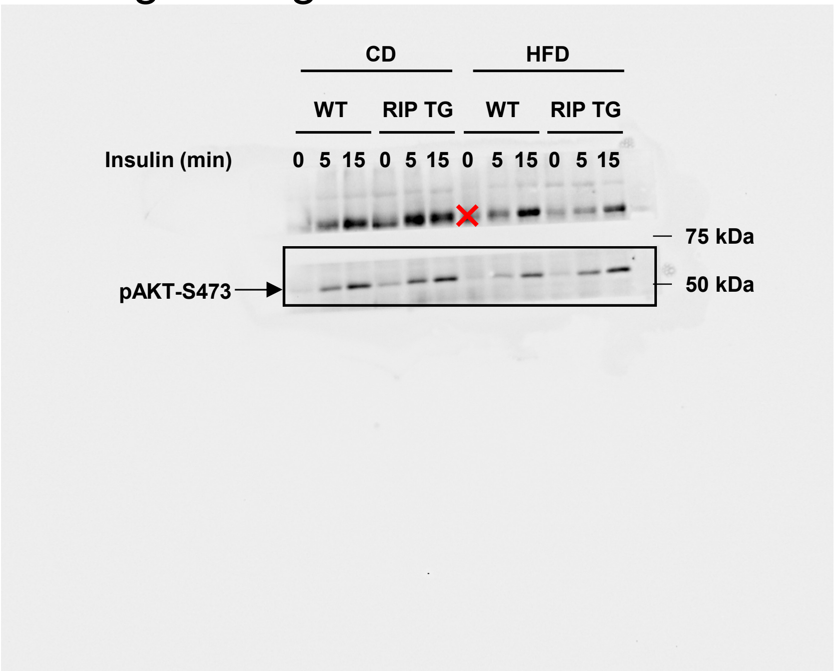

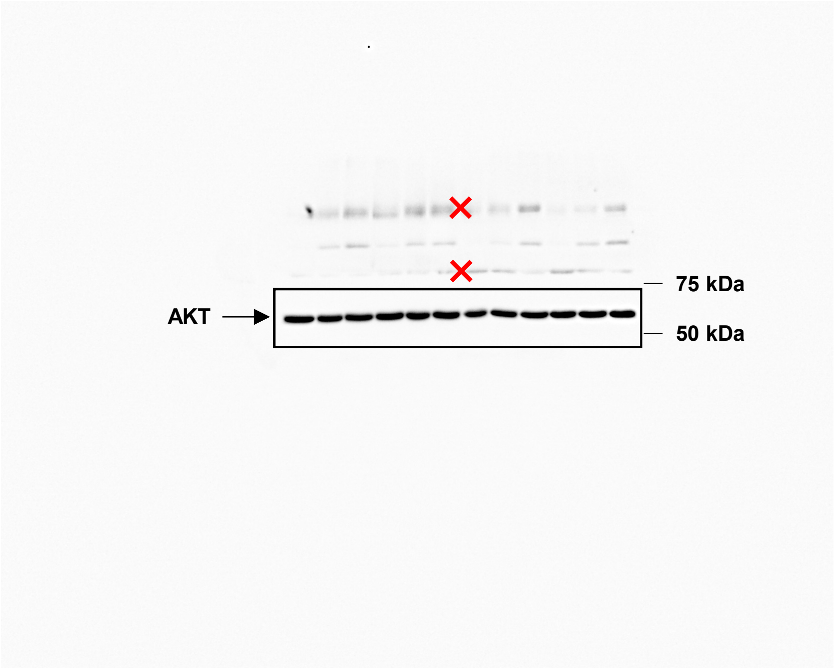

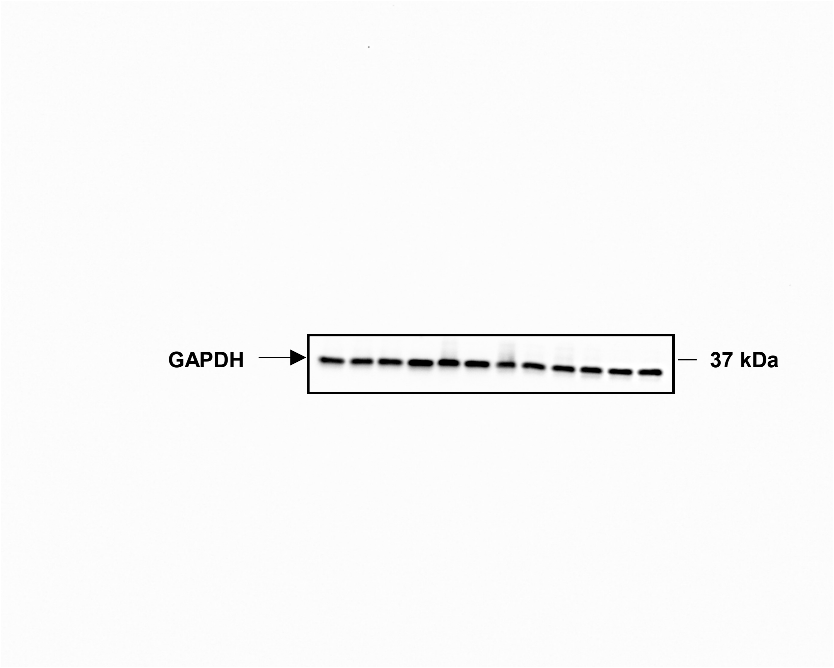

Original WB image in Figure 3C

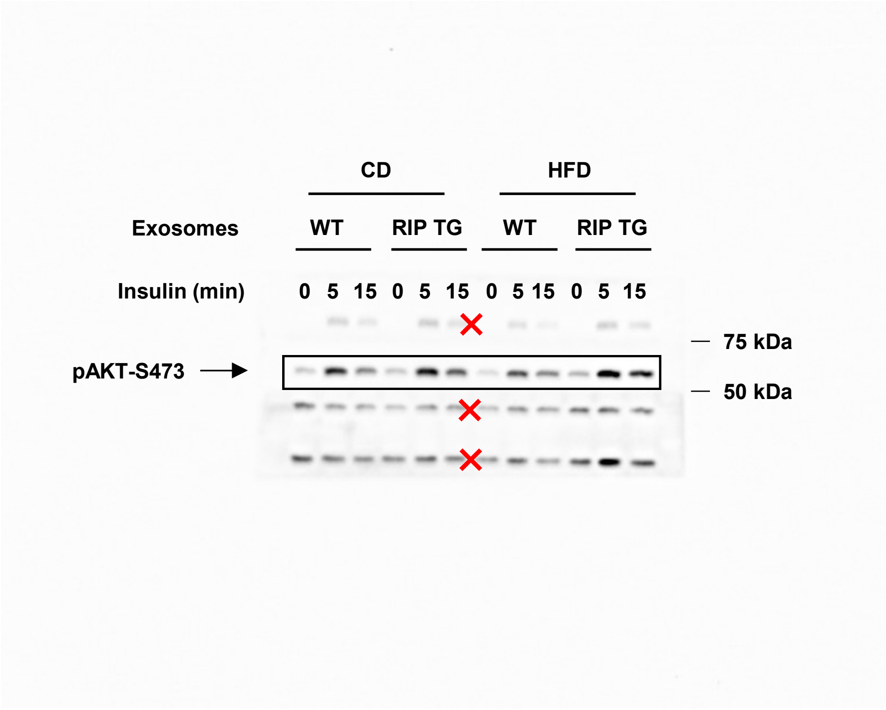

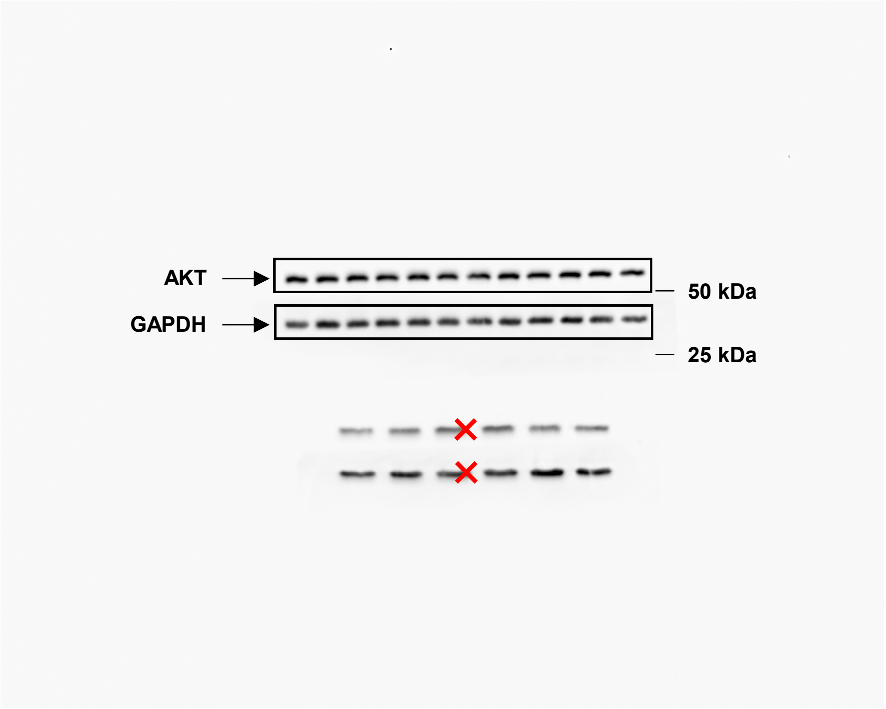

Original WB image in Figure 4B

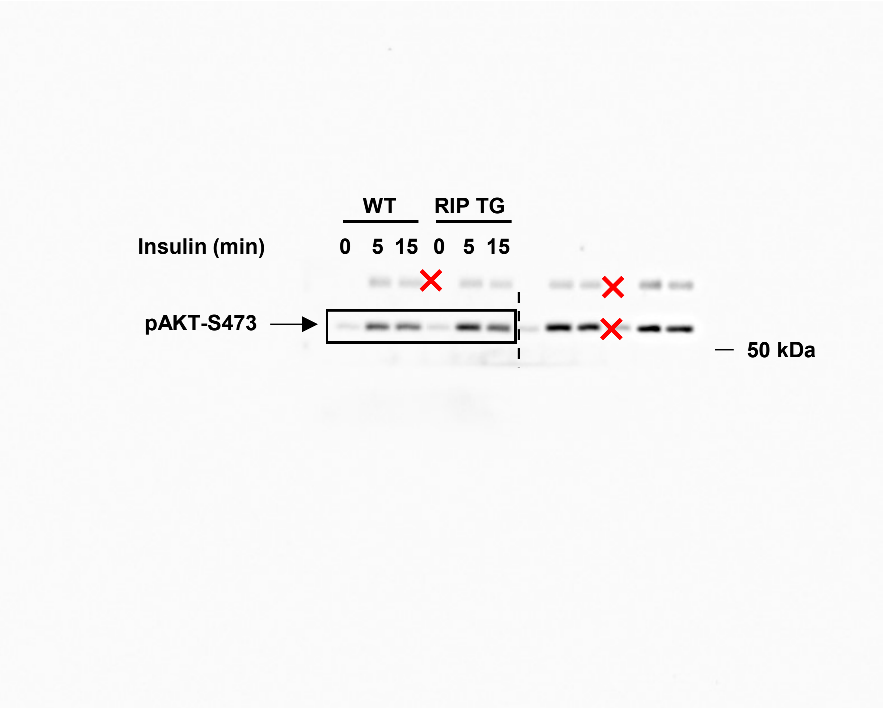

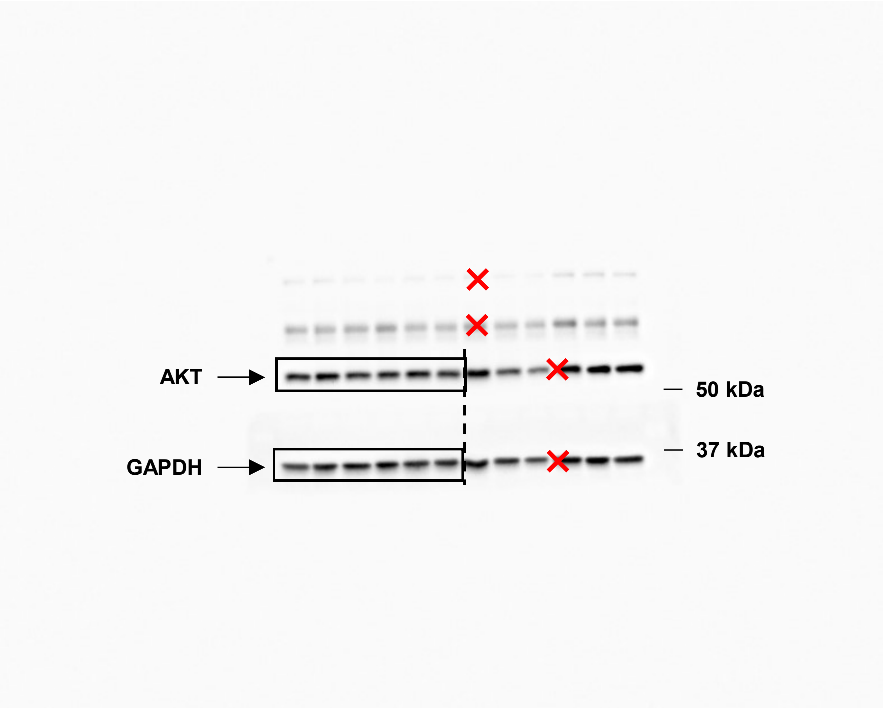

Original WB image in Figure 4D

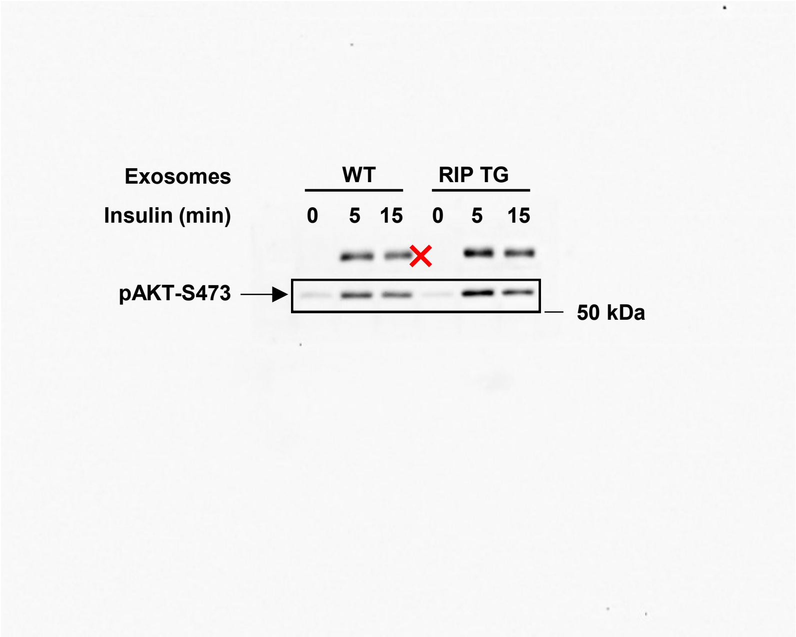

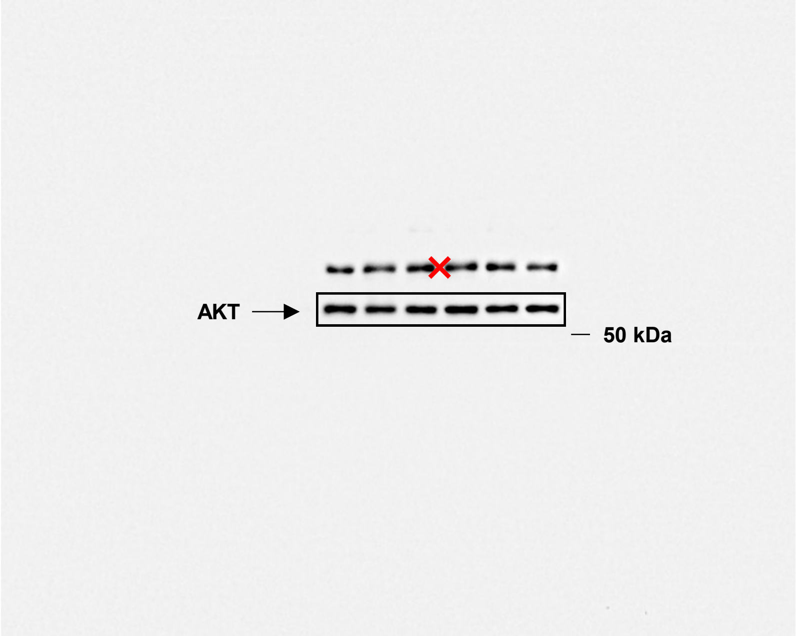

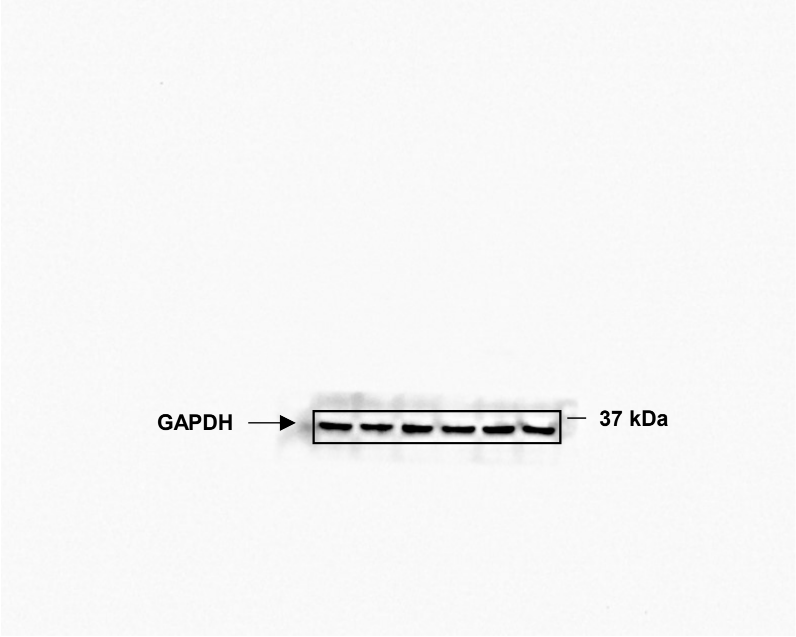

Original WB image in Figure 4H

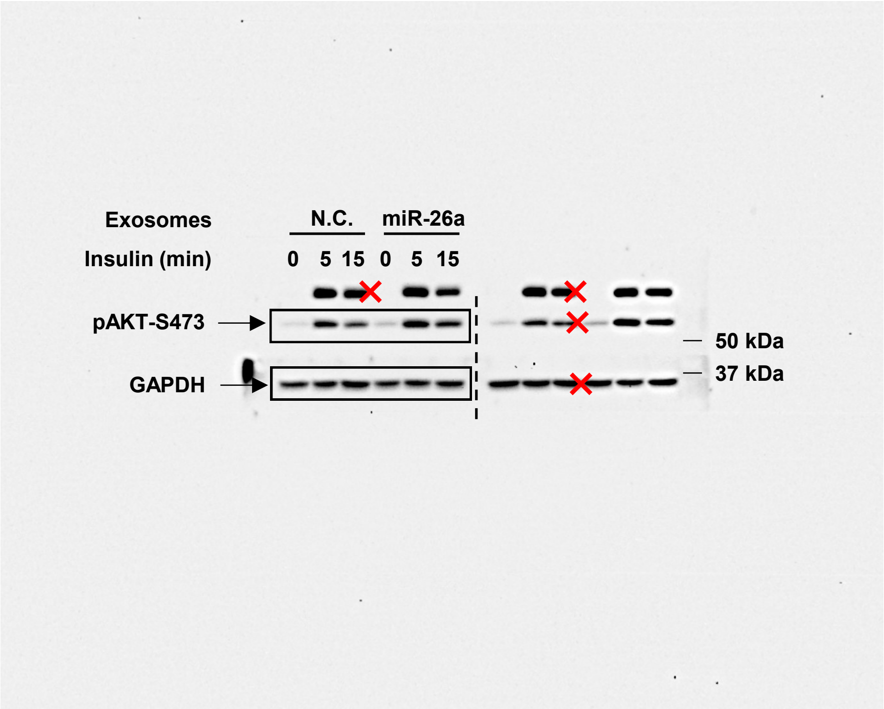

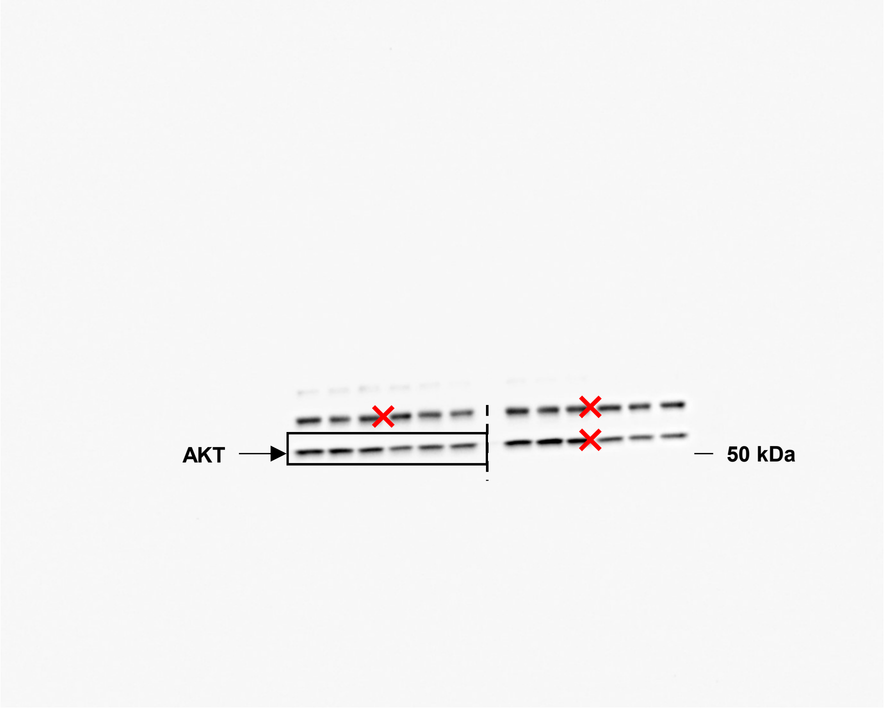

Original WB image in Figure 5C

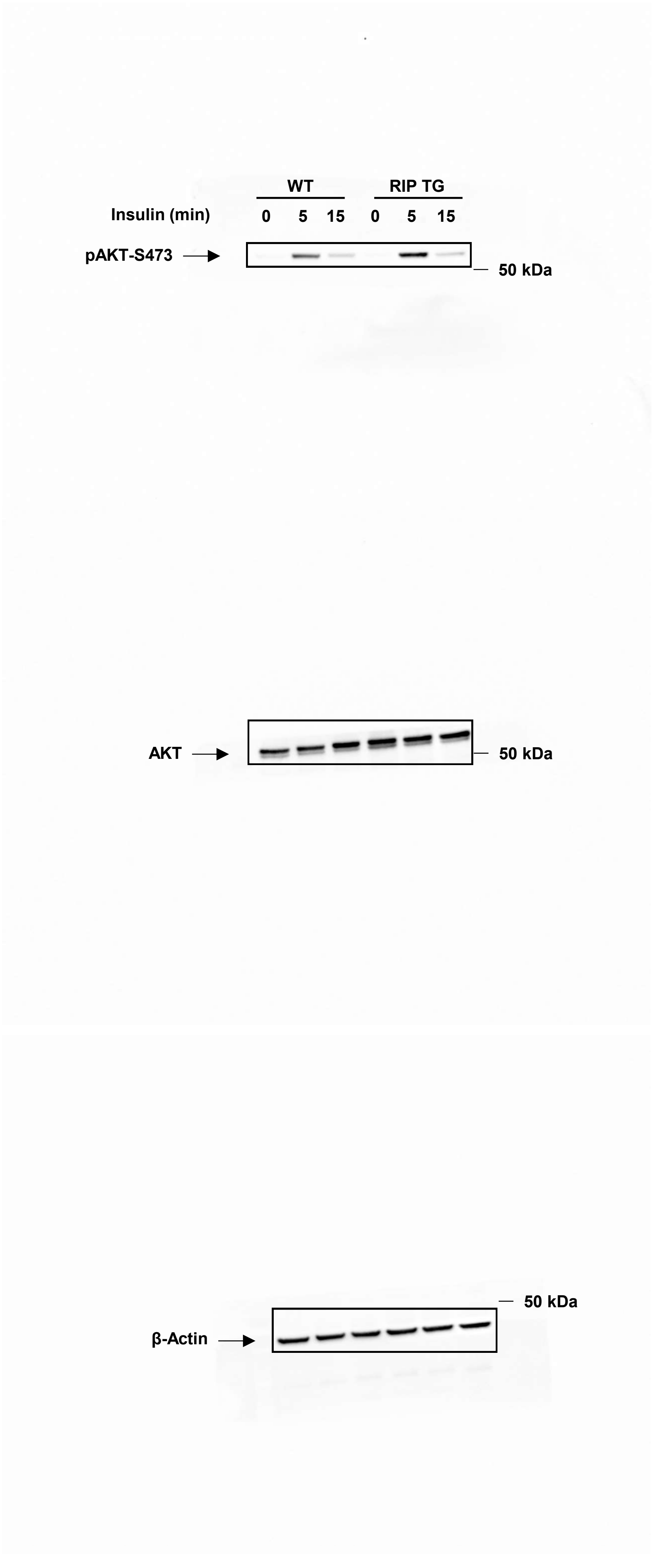

Original WB image in Figure 6F

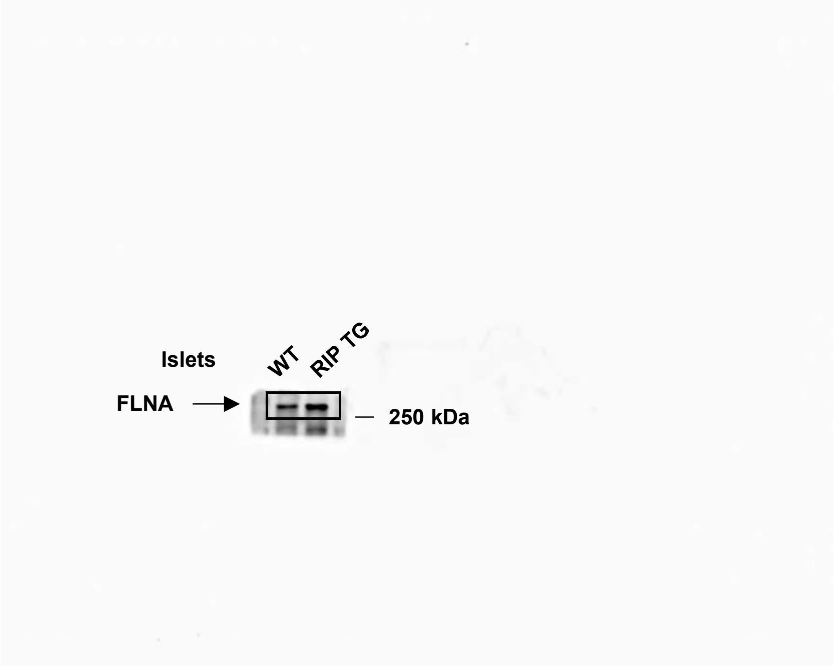

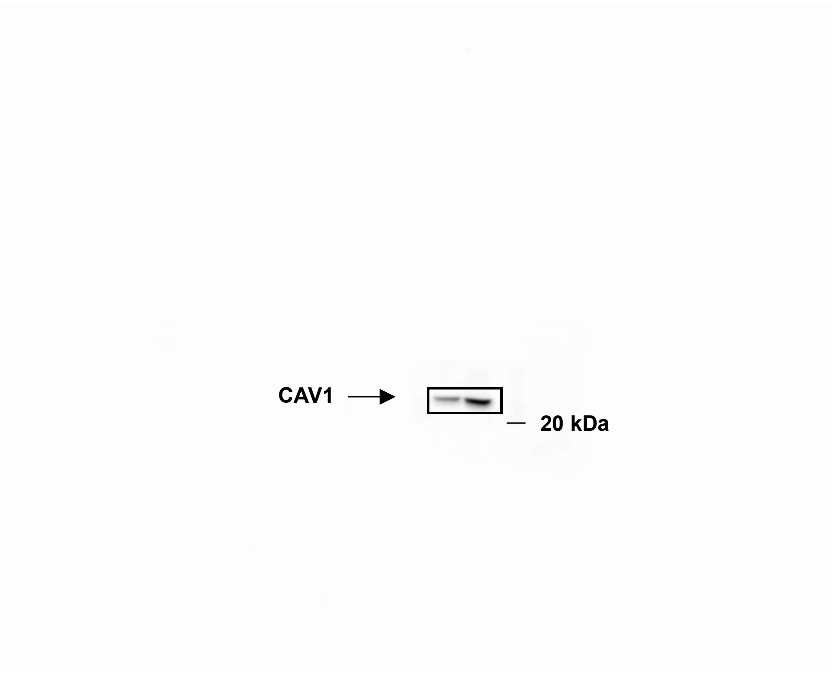

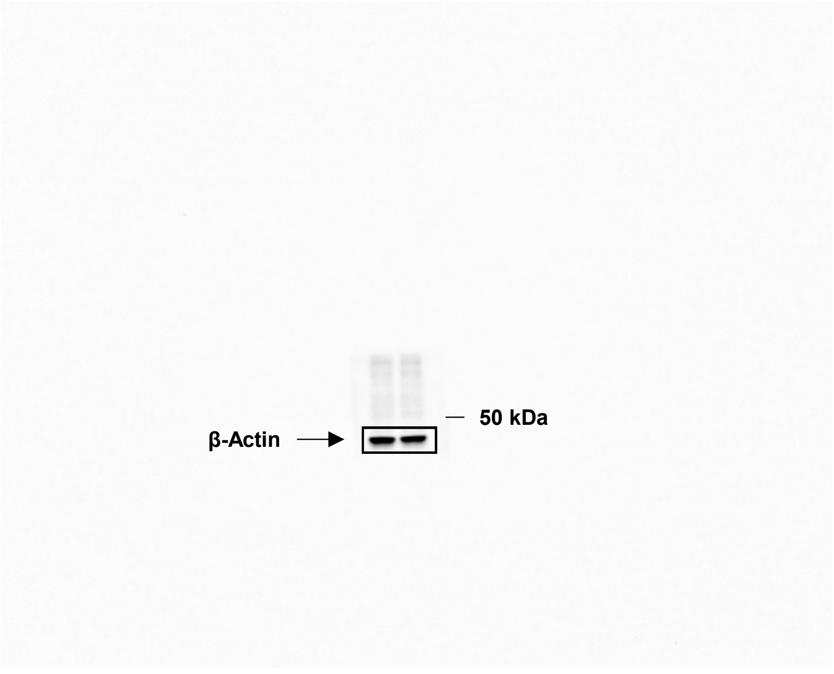

Original WB image in Figure 6K

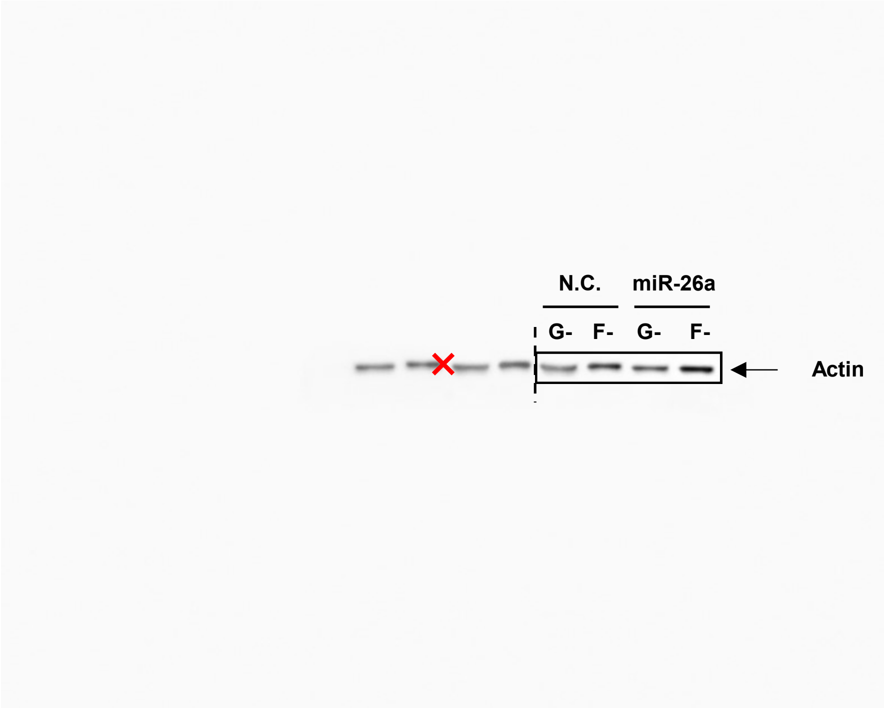

Original WB image in Figure 6L

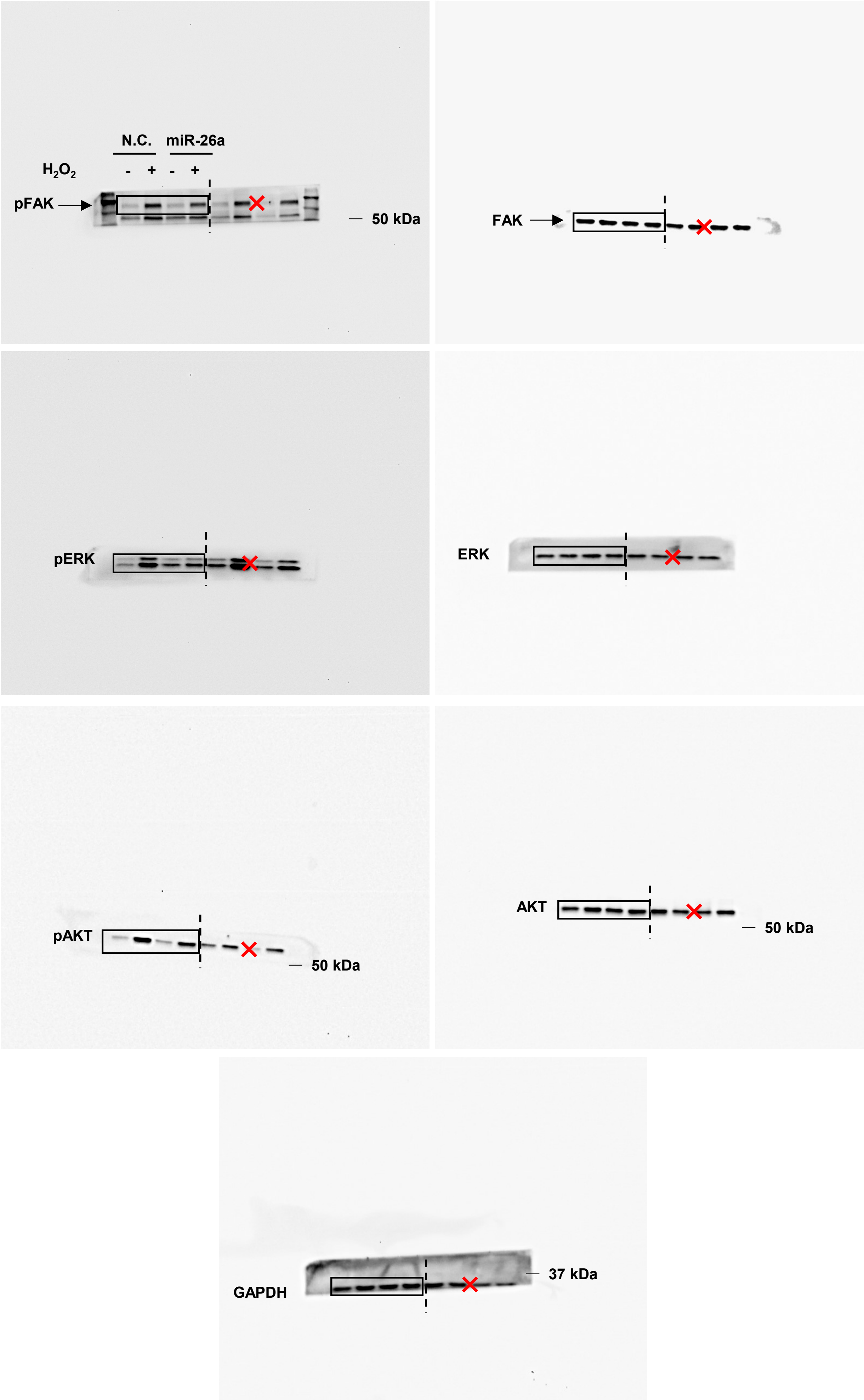

Original WB image in Figure 8L

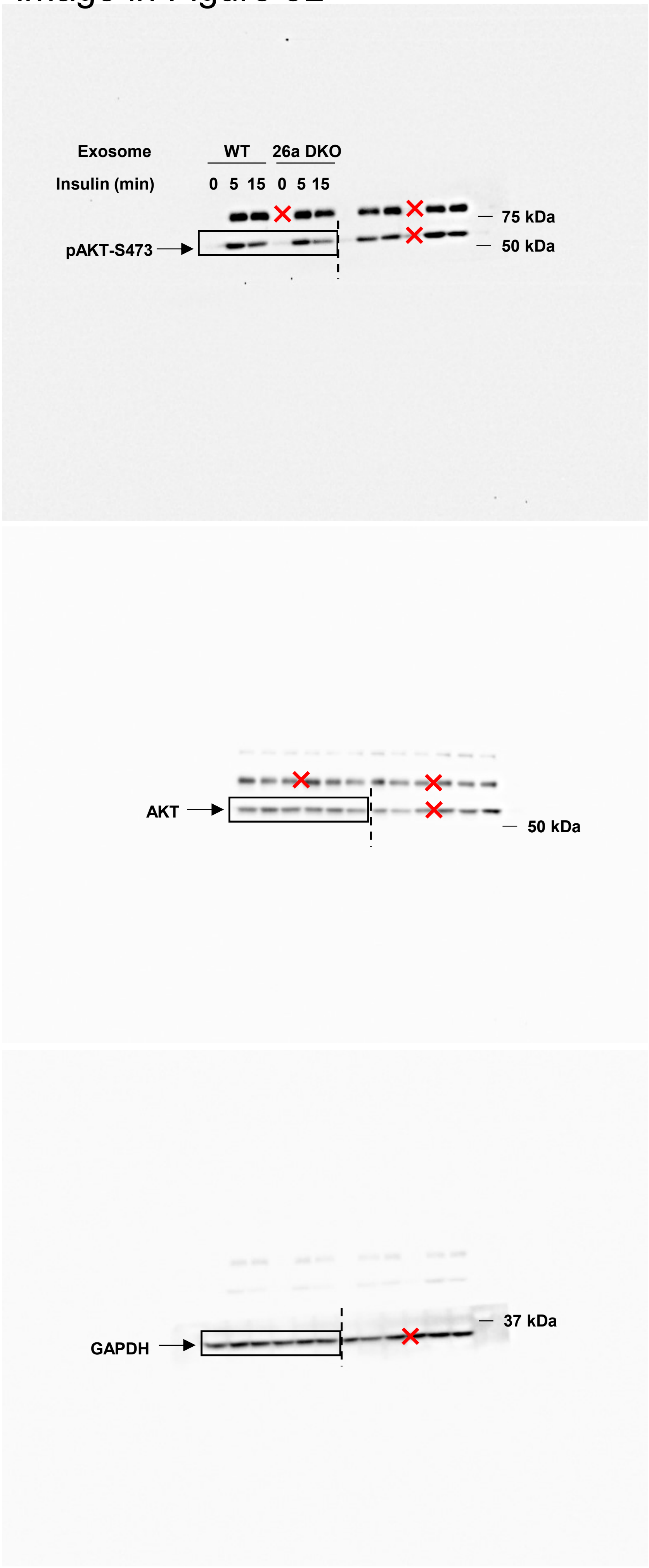

Original WB image in S1C Fig

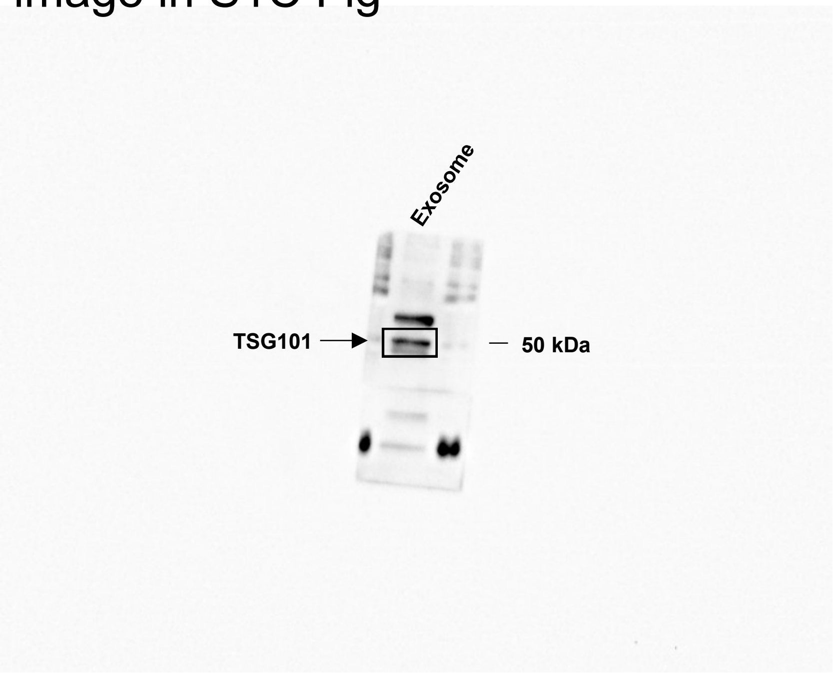

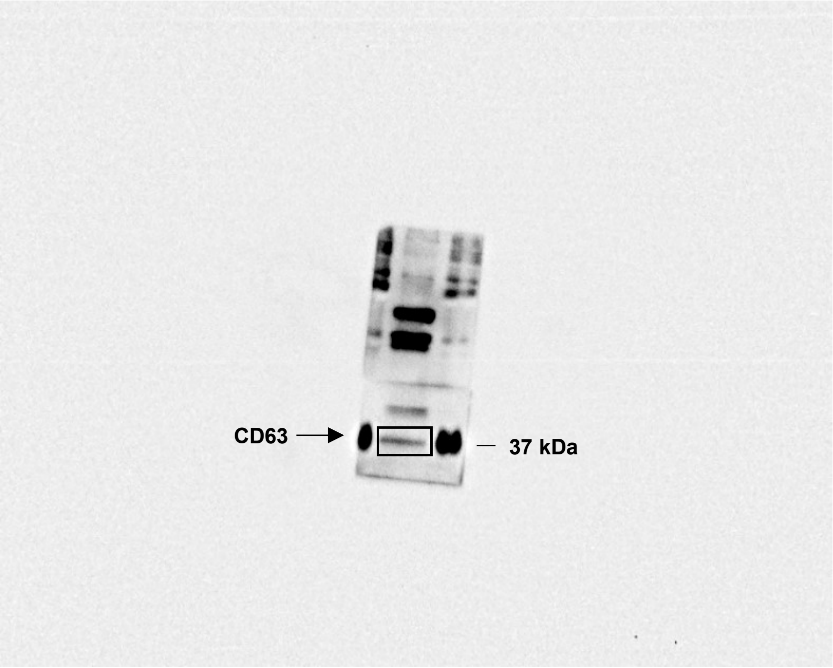

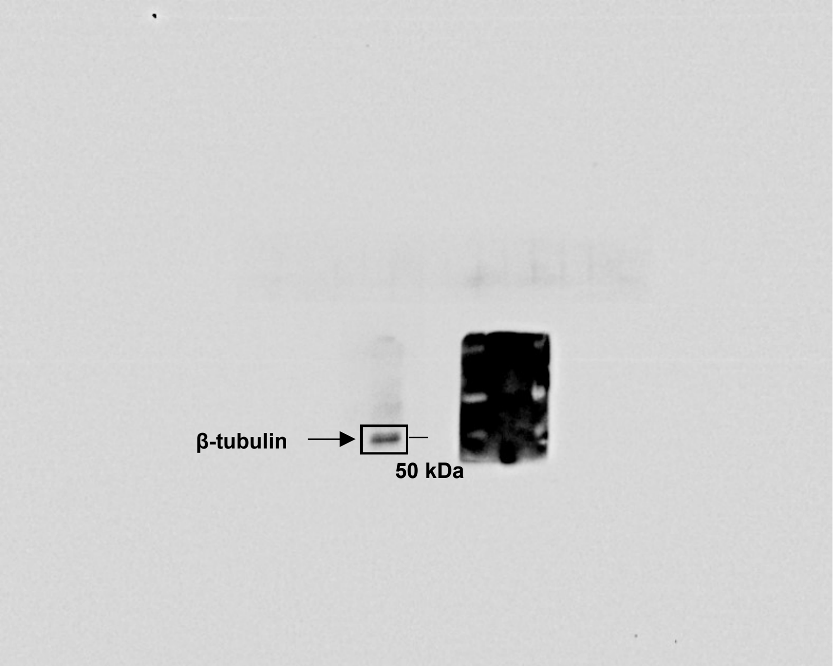

Original WB image in S9B Fig

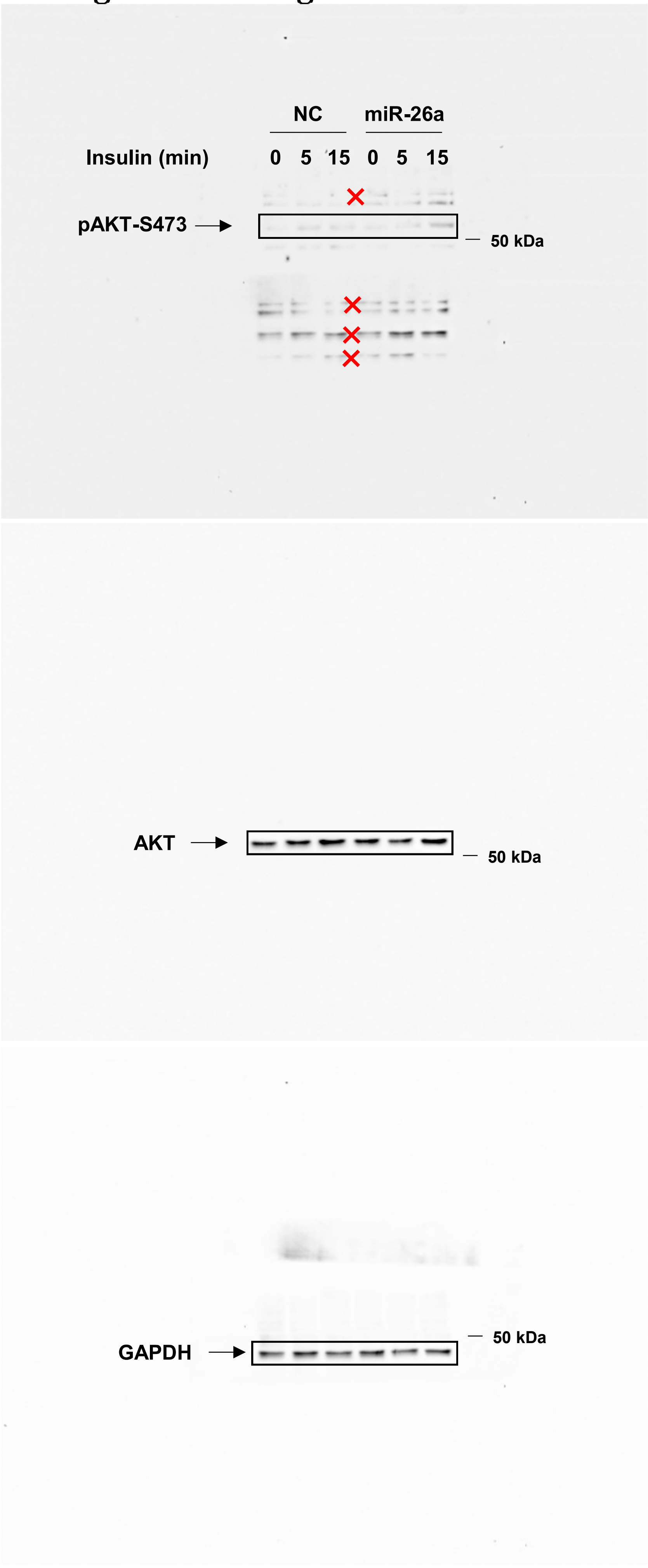

Original WB image in S9C Fig

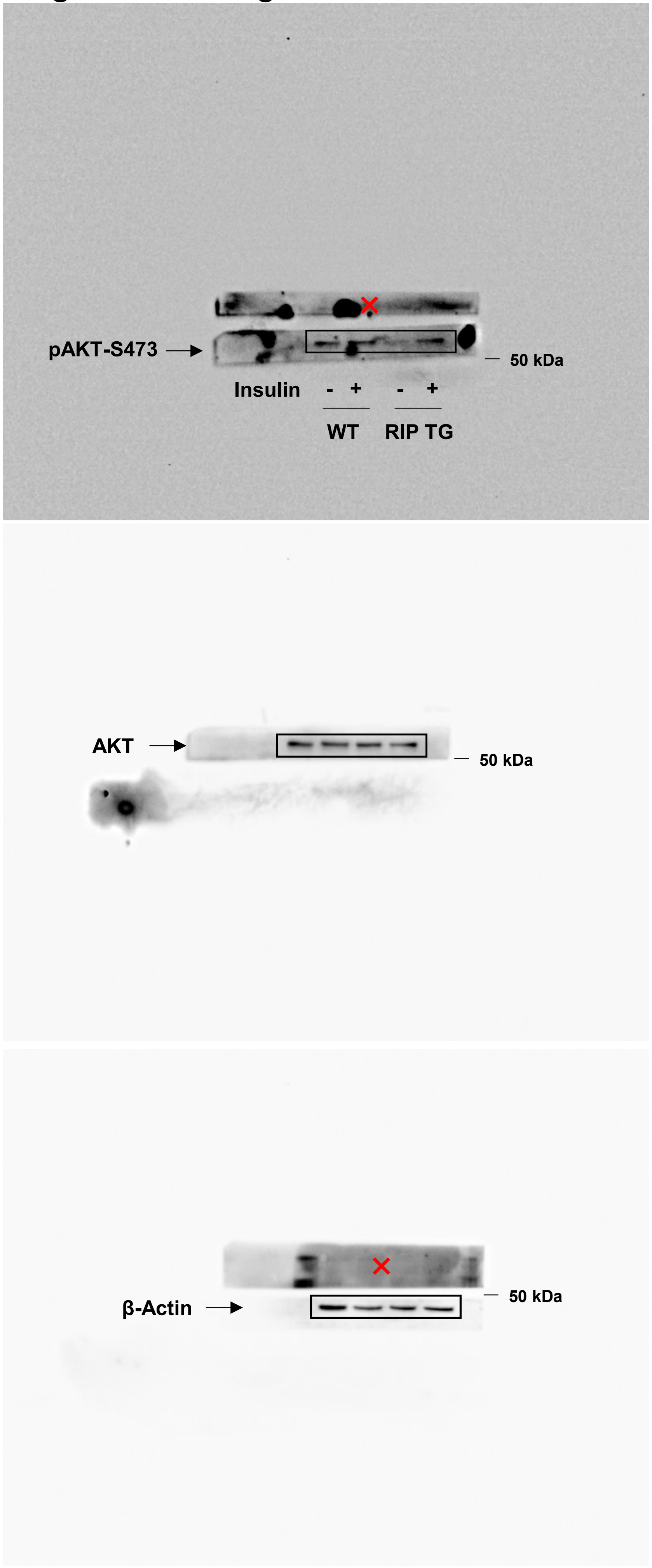

Supplement: S1 Raw Images — WB, western blotting. (PDF) [file pbio.3000603.s027.pdf]
